# Eyes Toward Tomorrow Program Enhancing Collaboration, Connections, and Community Using Bioinspired Design

**DOI:** 10.1093/icb/icab187

**Published:** 2021-08-30

**Authors:** Robert J Full, H A Bhatti, P Jennings, R Ruopp, T Jafar, J Matsui, L A Flores, M Estrada

**Affiliations:** Department of Integrative Biology, University of California at Berkeley, Berkeley, CA 94720, USA; Graduate Group in Science and Mathematics Education (SESAME), University of California at Berkeley, Berkeley, CA 94720, USA; Department of Integrative Biology, University of California at Berkeley, Berkeley, CA 94720, USA; Department of Integrative Biology, University of California at Berkeley, Berkeley, CA 94720, USA; Department of Integrative Biology, University of California at Berkeley, Berkeley, CA 94720, USA; Department of Integrative Biology, University of California at Berkeley, Berkeley, CA 94720, USA; Department of Social and Behavioral Sciences, University of California, San Francisco, CA 94118, USA; Department of Social and Behavioral Sciences, University of California, San Francisco, CA 94118, USA

## Abstract

The goal of our Eyes Toward Tomorrow Program is to enrich the future workforce with STEM by providing students with an early, inspirational, interdisciplinary experience fostering inclusive excellence. We attempt to open the eyes of students who never realized how much their voice is urgently needed by providing an opportunity for **i**nvolvement, **i**magination, **i**nvention, and **i**nnovation. Students see how what they are learning, designing, and building matters to their own life, community, and society. Our program embodies convergence by obliterating artificially created, disciplinary boundaries to go far beyond STEM or even STEAM by including artists, designers, social scientists, and entrepreneurs collaborating in diverse teams using scientific discoveries to create inventions that could shape our future. Our program connects two recent revolutions by amplifying Bioinspired Design with the Maker Movement and its democratizing effects empowering anyone to innovate and change the world. Our course is founded in original discovery. We explain the process of biological discovery and the importance of scaling, constraints, and complexity in selecting systems for bioinspired design. By spotlighting scientific writing and publishing, students become more science literate, learn how to decompose a biology research paper, extract the principles, and then propose a novel design by analogy. Using careful, early scaffolding of individual design efforts, students build the confidence to interact in teams. Team building exercises increase self-efficacy and reveal the advantages of a diverse set of minds. Final team video and poster project designs are presented in a public showcase. Our program forms a **student*-*centered creative action community** comprised of a large-scale course, student-led classes, and a student-created university organization. The program structure facilitates a community of learners that shifts the students' role from passive knowledge recipients to active co-constructors of knowledge being responsible for their own learning, discovery, and inventions. Students build their own shared database of discoveries, classes, organizations, research openings, internships, and public service options. Students find next step opportunities so they can see future careers. Description of our program here provides the necessary context for our future publications on assessment that examine 21^st^ century skills, persistence in STEM, and creativity.

## Introduction

"Workers of the future will learn to deeply cultivate and exploit creativity, collaborative activity, abstract and systems thinking, complex communication and the ability to thrive in diverse environments” (The Pew Research Center; Rainie and Anderson 2017). Learning these skills demands the reimagining of an undergraduate curriculum. In 2013, an AAAS report warned that research is at a tipping point transitioning from ultra-specialization and highly prescribed problems to one in which integrative and collaborative approaches are required to solve complex challenges. The Committee on Facilitating Interdisciplinary Research recommended that “undergraduate students should seek interdisciplinary experiences, such as courses at the interfaces of traditional disciplines that address basic research problems, interdisciplinary courses that address societal problems, and research experiences that span more than one traditional discipline. . .” (NAS 2004). The report on Enhancing the Effectiveness of Team Science (NRC 2015) adds that, “There are few opportunities to learn to collaborate effectively or understand science as a social and intellectual process of shared knowledge creation. . . At the undergraduate level, students majoring in science and the related STEM disciplines take courses dominated by lectures and short laboratory activities that often leave them with major misconceptions about important disciplinary concepts and relationships”. NAS (2015, 2017) reports strongly encourage integrating interdisciplinary course-based discovery experiences into the undergraduate curriculum. Institutional change is needed to “expand education paradigms to model transdisciplinary approaches for convergence” (NRC 2014). “Convergence is an approach to problem solving that cuts across disciplinary boundaries. It integrates knowledge, tools, and ways of thinking from life and health sciences, physical, mathematical, and computational sciences, engineering disciplines, and beyond to form a comprehensive synthetic framework for tackling scientific and societal challenges that exist at the interfaces of multiple fields”. More recently, a convergence workshop (NAS 2019) suggested that “early student exposure to convergence experiences, especially as undergraduates, could productively influence later interests and interactions with the academic system”.

### Bioinspired design meets the maker movement

An NRC report (2008) entitled "Inspired by Biology: From Molecules to Materials to Machines” concluded that bioinspired design is a strategy that has the potential to improve citizens wellbeing and the nations’ economic competitiveness. The approach uses principles discovered from biological systems to develop innovative materials, devices, structures, and systems. In President Obama's 2009 address to the NAS, he urged scientists to move out of the laboratory and into society to start a national movement to inspire and enable young people “to be makers of things”. Hatch (2014) stated that “The real power of this revolution is its democratizing effects. Now, almost anyone can innovate. Now almost anyone can make. Now, with the tools available at a makerspace, anyone can change the world”. Researchers of “makification” in education (Cohen et al. 2016; Hansen et al. 2020) “point out that the promise of the maker movement rests in its uniquely diverse communities with the encouragement of divergent mindsets that engage in multidisciplinary approaches to solve problems that are personally meaningful with potential to enrich meaning to those around them once they are shared”. Yet, studies note “trouble with the notion of makerspaces as an implicit panacea to equity and access issues in STEM” (Barton et al. 2017) unless we include “a broader range of identities, practices and environments” that represents “a bold step toward equity in education” (Vossoughi et al. 2016). Therefore, we must explore areas of connected, interest-powered, and communal utility value learning (Brown et al. 2015a, 2015b) in the context of culturally sustaining pedagogy (Paris 2012).

### Eyes Toward Tomorrow Program

**Fig. 1:**
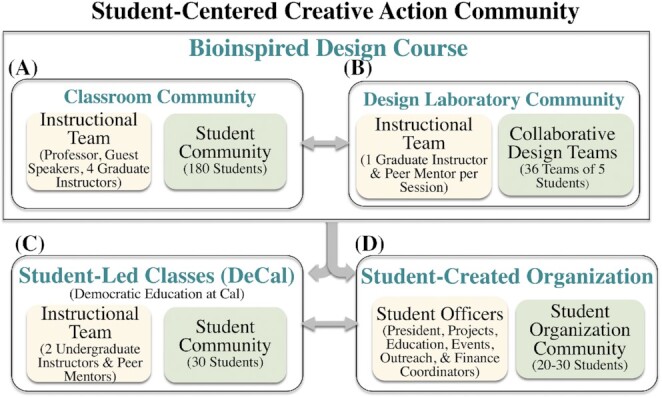
Eyes Toward Tomorrow Program communities. **Bioinspired Design course**includes (**A**) Classroom community (two 1-h lectures per week, demonstrations using personal response system where teams share connections) and (**B**) Design laboratory community (one 1-h design session per week with access and training to all makerspace equipment). (**C**) **Student*-*led classes**(DeCal; 1-h lecture per week with project-based designs, demonstrations, and makerspace training), and (**D**) **Student-created organization** (weekly meetings, projects, guest speakers, design workshops, design-a-thons, field trips, collaborative activities, and competitions.)

We combine the power of interdisciplinary approaches and the maker revolution in our Eyes Toward Tomorrow Program, grounded in scientific discovery and bioinspired design taking place in our educational makerspace (i.e., The Jacobs Institute of Design Innovation). We attempt to open the eyes of those who never realized how much their voice is urgently needed by providing an opportunity for **i**nvolvement, **i**magination, **i**nvention, and **i**nnovation in a student-centered creative action community. The center-piece of the community is our **Bioinspired Design** course that is part of U.C. Berkeley's College of Letters and Science's breadth requirement and serves 180 students per semester (Fig. 1A, B). Demographics show we have 60% female students with nearly 50% of the class in their first or second year. The course has no prerequisites and receives institutional matching funds for graduate student instructors and supplies. The course supports students from over forty different majors, including molecular and cell biology, public health, integrative biology, data science, bioengineering, mechanical engineering, electrical engineering and computer science, art, architecture, chemistry, sociology, psychology, political science, and business. Students who take our large-scale course are encouraged to teach their own course (DeCal class; Fig. 1C) and join a student-created, student-led, and university recognized organization—Berkeley's BioDesign Community—Bio-D (Fig. 1D). In the DeCal class, students teach their own class with a different emphasis such as enhanced industry networking, K-12 outreach, longer-term design projects, or team participation in national competitions. Student instructors have access to all the resources offered by the university to faculty-taught courses (Fig. 1C). Regardless of whether students have taken either of our courses, they can join our Bio-D organization as participating members or elected officers (Fig. 1D). The Bio-D community enables an even greater diversity of students to benefit by inviting interactions with other campus organizations in engineering, art, architecture, and business. These synergies lead students to explore previously unimagined paths by then taking our large-scale course or student-led classes.

Our program is the pedagogical realization of the PolyPEDAL Laboratory's interdisciplinary research approach to bioinspired design. It represents a natural next step in sharing our vision with a large, diverse undergraduate population after founding our interdisciplinary Center for Bioinspiration **i**n Education and Research at Berkeley (C**i**BER). C**i**BER previously offered a 20-student, upper division, discovery-based learning laboratory that resulted in authentic research (Full et al. 2015). We contend that research and teaching are inseparable communities. Our program strives to inspire the next generation to expand the sphere of interdisciplinary creativity far beyond biology and engineering.

The NAS (2021) report declared that “Scientific thinking and understanding are essential for all people navigating the world, not just for scientists and other STEM professionals”. To develop a more creative and inclusive **STEM-enhanced** workforce, and to leverage diverse representation as a distinct competitive advantage in today's age of innovation (NAE 2015; AlShebli 2018; Hofstra et al. 2020), we propose seven aims where students:

Aim 1: Understand scientific discovery using bioinspired design;
Aim 2: Create inventions with societal relevance through design assignments and laboratories;
Aim 3: Learn teaming where each voice is valued using a series of team-building exercises;
Aim 4: Connect to communities to see career paths by building a shared database of discoveries, classes, organizations, role models, and next step programs, while teaching their own course, and leading or joining a student-created organization;
Aim 5: Participate beyond our campus by adapting our curriculum and materials using outreach and broad dissemination venues;
Aim 6: Own the program through feedback using surveys and formal assessments to which we respond;
Aim 7: Develop 21^st^ century skills that include critical thinking, creativity and innovation, communication and collaboration, and interdisciplinary thinking (Trilling and Fadel 2009).

## Aim 1: Understand scientific discovery using bioinspired design

**Fig. 2:**
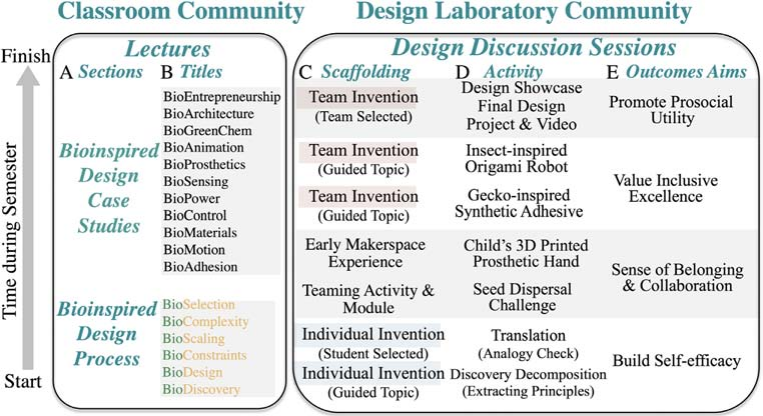
Bioinspired Design course. Time period over the semester shown from start (bottom) to finish (top). Lectures. (**A**) Two sections of lectures are delivered. (**B**) Lecture titles. (**C**) Scaffolded assignments that progress from individual (gray boxes) to team (tan boxes) and from guided topic to student or team selected. (**D**) Specific individual or team activity. (**E**) Outcome aims for each set of activities.

We aim to share the process of discovering nature's principles by designing course activities that advance students’ science literacy. NAS (2021) reports “scientific literacy elevates the quality of decision making in almost every aspect of daily life” and “understanding science and the practice of scientific thinking are essential components of a fully functioning democracy”. We aim to ensure that all students are scientifically literate citizens who know how and where reliable information originates. Therefore, the foundation of our program resides in original discovery. We use the researcher's frame to situate student learning to best understand the scientific process (Lave and Wenger 1991). We begin our lectures with those focusing on the Bioinspired Design Process (Fig. 2A, B).

## Bioinspired design process

To provide the biological foundation necessary to effectively extract and translate principles, we give a series of lectures that focus on biological discovery, biodesign, bioconstraints, bioscaling, biocomplexity, and bioselection (Fig. 2A, B; see supplement S1 for the concepts highlighted in each lecture).

### BioDiscovery

We want students to experience bioinspired design through the lens of original discovery where they “become” the researcher and sense the excitement of discovering something no human has ever known before. We use a solution-driven approach where inspiration begins with a biological discovery, extracts the fundamental principle, and then creates an analogy to use this principle to solve a human problem (Helms et al. 2009).

We complement our BioDiscovery lecture with design laboratory discussions focusing on scientific journals, the differences between primary, secondary, and tertiary sources, unreliable sources, search engines and strategies, the anatomy of a scientific publication, and the manuscript and grant review process. This analysis and simplification of the scientific communication landscape is often the first time many students are exposed to the foundations of the scientific process. Even more critical, it may be the last time, especially for non-STEM majors.

We dispel the myths and stereotypes that science is a boring career, primarily conducted in isolation and often on seemingly obscure topics. Science is a dynamic process involving curiosity, creativity, exploration, and discovery, gathering and interpreting data, with energizing communities offering feedback and new ideas for collaboration, and maintaining the possibility of contributing to revolutionary societal benefits and outcomes (Understanding Science 2021). We share narratives about the discovery process, what it is really like to tackle grand challenges, collaborate with colleagues, enjoy social interactions, fail at times, but truly realize one's dreams of discovery. Part of the excitement is that one never knows where curiosity-based research will lead. The most significant breakthroughs come from studies one least expects. To inspire, motivate, and connect with students, we use stories of real scientists and their discoveries from diverse fields (Avraamidou and Osborne 2009; Padian 2018).

In our **BioDesign** lecture, we provide students the tools to use a solution-driven, bottom-up approach to extract biological principles for translation from original biological discoveries. Our approach directly addresses previously stated obstacles most often observed in engineering and design courses (Graeff et al. 2020, 2021; Lenau et al. 2018; Nagel et al. 2019). “In practice, it has been acknowledged that challenges exist for designers and engineers in accessing and interpreting principles in life sciences through academic literature” (Rovalo et al. 2020). We developed a simple method, *Discovery Decomposition*, to understand a scientific publication (see Supplement S2). Using a flow chart format, we see students with no science background navigating the maze of jargon and data in scientific papers by mapping what was known, done, measured, and discovered, to come away with the biological principle they can use for design. Our simple *Analogy Check* process ensures students identify the similarities and differences present in the organism compared to their proposed design related to structure, size, operating environment, mechanism, specification, performance, and constraints (see Supplement S2).

The remaining foundational lectures of the bioinspired design process (Fig. 2A, B, bottom) are further developed in Supplement S1. In the **BioConstraints** lecture, we show students the concepts needed to navigate the results of evolution. Many students, engineers, and bioinspired design courses assume nature's creatures are optimally designed by evolution and should be copied or mimicked. We shatter these myths through lectures detailing how evolution is not engineering and directly debunk intelligent design. In **BioScaling**, we reveal why students must consider size to develop the most effective analogies. In **BioComplexity**, we demonstrate the value of mathematical and physical models that collapse biological dimensions. In **BioSelection**, we advise on how to select a system or organism for inspiration.

## Case studies of bioinspired design

After the foundational lectures, students are eager to see the process applied to specific case studies of interest (Fig. 2A, B, top). Students demand connections meaningful to them beyond those typically discussed in biology or biomimetics (Chamany et al. 2008; Canning et al. 2018; Priniski et al. 2018). Students attracted to engineering see new career opportunities for environmental monitoring, hazard detection, and search-and-rescue from our **BioMotion** lectures on running, flying, and swimming animals that lead to the next generation of mobile robots. Those passionate about health and medicine see never imagined career trajectories in our **BioProsthetics** lecture, which features cochlear and retinal implants, exo-suits allowing people with paraplegia to walk again, and brain-machine interfaces permitting those with quadriplegia to move robot arms to feed themselves. Environmentally conscious students are inspired by the biodegradable and sustainable materials shown in our guest lectures on **BioGreenChem** and **BioArchitecture**. Students who love art but did not envision how a STEM-enriched career path could allow them to realize their dreams see examples in our **BioAnimation** lecture. We tell stories of how we helped Pixar and DreamWorks make children's movies using biological principles of motion applied to computer visualization through big data science and artificial intelligence.

## Aim 2: Create inventions with societal relevance through design assignments and laboratories

Students best learn through a constructivist approach that promotes active learning (Papert 1999; Freeman et al. 2014; Theobald et al. 2020). In our course, this is accomplished by making conceptual designs and prototypes inspired by biology. We provide an opportunity for self-generated, utility-value tasks (Canning and Harackiewicz 2015; Priniski et al. 2018) to help students see how what they are learning, designing, and building matters to their own life, community, and society. These tasks challenge students to reflect upon the course material through the development and invention of their own bioinspired designs. Underscoring each design are connections to widespread societal benefits, leading to societally-relevant designs. By ensuring that learning truly matters, our program fosters a sense of science activism amongst students, including nonmajors who, along with their STEM major counterparts, will make up a future citizenry that must be able to apply scientific knowledge and practices to make informed decisions as a part of their duties in a democratic society. Thus, we view learning within our program as not being confined to STEM, but “STEM-enriched”, applicable to areas within and outside of STEM.

To accomplish this aim, we offer a series of scaffolded discussions with design laboratory sessions each week to directly complement our lectures (Fig. 2D). We begin with individual assignments to improve mastery of the approach and foster connection by encouraging students to select a personally-meaningful discovery for inspiration (Fig. 2C, bottom). We follow individual assignments with team building activities (Fig. 2C, middle) and exercises where we assign published discoveries to guide group designs. After inspiriting collaboration, teams select discoveries to inspire a novel invention that they present in brief video and poster formats at our public, end-of-semester Design Showcase (Fig. 2C, D, top).

## Scaffolding individual strengths to aid in the discovery to design path

Early in the course, we rely on scaffolding strategies using low point value formative assessment (Maksic and Smiljana 2021). We provide individual assignments using the bioinspired design process to increase student creativity and confidence, while convincing them that they have an essential voice in the upcoming team design assignments. Students build upon lecture examples of how to extract a principle from a scientific paper (i.e., Discovery Decomposition) and propose a novel design (i.e., Analogy Check) by using our second assigned publication on the discovery of gecko adhesion (Autumn et al. 2002) as an individual homework assignment (Supplement S2). All students submit their paper breakdown but are free to create a novel design by completing the Analogy Check. In the next individual homework assignment, we reduce the scaffolding by allowing students to complete a primary literature search for a scientific paper that most interests them, extract the principle discovered, and propose a novel design. Discussion section time is allotted to support these assignments by first dedicating a session to searching scientific literature. The next two sessions focus on the scientific process of discovery, including research question origination, experimental design, scientific publication, the peer review process, and creating and giving scientific meeting presentations. Graduate student instructors directly mentor students, respond to questions, and grade each individual design with encouraging written responses to build self-efficacy before the undergraduates undertake team design projects.

## Guided topic, team design projects

After individual design projects, we form teams and lead students through teaming activities and collaboration training (see Aim 3). We offer two guided topic, design laboratories—Gecko Synthetic Adhesives and Insect Inspired Robots (Fig. 2D). First, we extend the scaffolding in lecture and homework assignments using gecko adhesion publications by asking teams to create a synthetic gecko-inspired adhesive using a polymer mold and then test its properties in a series of experiments (Supplement S3). In the next laboratory period, teams are given the opportunity to create a novel conceptual design using their adhesive. To ensure that biologists, engineers, architects, artists, business majors and more all can contribute to a design, we encourage combinations of three media for design representation. Teams can select from: (1) mock-up construction material provided (i.e., construction toys, Styrofoam, cardboard, construction/craft material with tools), (2) computer simulation, blueprint, or artistic sketches (e.g., Photoshop, SketchUp, Maya, Blender, 2D/3D CAD tools, Solidworks, Autocad, or Adobe Illustrator); and/or (3) prototype building using our Makerspace facilities (e.g., laser cutters, 3D printers, CNC routers for woodworking, sewing, or sculpting). These activities are made possible through a partnership with a campus makerspace. Each student receives a maker pass, hands-on safety training, and free hands-on training on all makerspace equipment in our institute (i.e., Jacobs Institute of Design Innovation). All teams use a combination of approaches and submit both a report and their design for assessment. Examples of gecko adhesion-related trial designs included a stroke rehabilitation device with easy grip, a puncture-proof organ gripper for surgical transplants, shower sandals for the elderly, a diabetes pump attachment with easy on and off, and an attachable grab bar for the disabled. For the second guided team design project, students use lectures on terrestrial BioMotion, BioControl, and BioMaterials to aid in the construction of an insect exoskeleton-inspired origami legged robot called DASH (Supplement S4). The robot comes in a laser-cut flat sheet that teams punch out and assemble with a provided electronics board, allowing students to drive the robot using a bluetooth-connected mobile app. Students then characterize the robot's performance across a series of testing experiments. Again, teams use all construction approaches to modify their robot for a novel trial design. Examples of trial designs include a mobile agricultural soil monitoring robot, DEToxES (Dynamic Environmental Toxins Elimination Sensing) robot, a humanitarian demining robot, a disinfecting robot for hospitals, and a “St. Bernard” robot for search-and-rescue and disaster relief.

## Team selected design projects

After students have completed individual and team design projects, they are sufficiently prepared to complete the final course project (Fig. 2D, top; Supplement S5). Teams have three weeks to select a benchmark biology publication, gain approval from instructors, extract the biological principle discovered (i.e., do a Discovery Decomposition), translate the principle to a conceptual design (i.e., do an Analogy Check), create a feasibility prototype, and produce a 5-min video and poster explaining the scientific publication, the challenges of translation, and a commercial pitch for their proposed product possessing societal benefit. Final design projects have included a flexible cast to reduce muscle atrophy based on the skeleton of seahorses, fresh-water capture devices based on a camel's nose or a lizard's skin, a voice restoration system for throat cancer patients based on a songbird's syrinx, and a compliant novel suturing device derived from porcupine spines. Final designs projects are presented to the public in a Design Showcase hosted at our Jacobs institute and online after the last week of classes. We offer support for teams interested in competing in national and international bioinspired design contests the next semester. Follow-up efforts are formalized in our student-led classes and/or community (Fig. 1C, D; see Aim 6).

## Aim 3: Learn teaming where each voice is valued

Effective teaming is a critical skill for creativity in science and innovation in our future workforce (NRC 2015). We place students into teams and provide team training in design sessions. We use our online module (https://www.teamingxdesign.com) to engage in team building and feedback activities, and teach how to develop a collaborative plan to coordinate final design projects.

## Team composition

We compose teams of five students based on their responses to a survey that captures their disciplinary backgrounds and expertise. From the survey data, we make an effort to balance area of interest (biology, engineering, design, architecture, and business), level (freshmen to seniors), and design experience (skills, tools, and classes) in every team (Supplement S6). We do not use students’ ethnicity, gender, economic background, or other sociodemographics when composing a team, but plan to explore how these demographics may relate to team dynamics and performance. The teams remain the same for the entire semester.

## Team training

We invite each student to create a shared Social Mixer Slide (analogous to an “About Me” slide) to facilitate team dynamics. These slides include opportunities to express personal creativity through items like selfies, a 1-min video introduction, and a 3-word/image description, along with technical data like major, year of graduation, future goals, experience with design/presentation, and which BioSystem and design challenge most interests them. We then make a compiled PowerPoint file gallery available to the team or discussion section. A major advantage is that both the course and discussion section instructors get to know the student sooner and more effectively than through typical first-day introductions.

Fortunately, we have a campus group that has researched (Lau et al. 2012) and created a “Teaming with Diversity” toolkit. The module includes an eight-step program supplemented by three brief and easy-to-follow, videos that include “Why learn teaming”, “How can our team succeed”, and “How can our team excel”. We review teaming advice in a design laboratory and then make teaming videos available online. A critical component of effective teamwork is reflection, defined as a group's ability to share team objectives, strategies, and processes collectively, and then adapt accordingly. We hope to devote more structured development of team dialogue and inclusive feedback in the future, including activities where team members listen actively to learn. For instance, we encourage team members to avoid binary listening and judgment (i.e., yes/no feedback) and instead learn to ask, “how might this be possible?” Rather than disagreeing outright, students try to imagine, “what could that allow us to do?” Instead of judging whether something is a perfect fit, they ask, “how can that contribute?” Rather than see something as good or bad, students ask, “what avenues could that idea advance?” Moreover, instead of judging things as realistic or unrealistic, students try to imagine, “what can you see that I do not see?” These critical aspects of active listening are seldom taught or promoted through dedicated teaming.

## Teaming activities encourage collaboration and self-efficacy

After teams are formed, their first activity is a cooperative challenge task to promote teaming, particularly within the context of a biological phenomenon. In this first teaming activity, students consider how dispersal of seeds is crucial for the survival of plant species (Supplement S7). Plants that rely on seed dispersal need to maximize their ability to disperse seeds relative to their anatomy and size constraints. Greater height of the seedhead increases the probability of dispersal, but also increases the probability of stem collapse—an example of the trade-off's organisms face. With this biological framing in mind, we challenge teams to build the tallest freestanding plant measured from the tabletop surface to the top of the “seedhead” (i.e., a marshmallow) using as many or as few of the stem structures (spaghetti sticks) and as much or as little of the string/tape as they prefer in only 18 min (Wujec 2010). We follow-up the challenge with team discussions regarding strategies, emergent roles, individual workload, and how to increase team effectiveness in the future. We end the exercise by asking students to reflect on what they did well as a team and what they could improve upon to communicate more effectively.

As teams become more familiar with one another, we task them with a 3D printing activity meant to promote students’ self-efficacy (Supplement S8). We developed this activity based on student feedback recommending an early makerspace experience during pilot versions of the course. Inspired by these requests and utilizing an open-source design, each student 3D prints a finger of a prosthetic hand designed for children affected by symbrachydactyly. Each student submits a selfie holding their printed finger in front of the 3D printer. Each printed finger is then used in a collaborative, team-based assembly of all parts of the 3D printed prosthetic hand, resulting in a fully functional final product. Student survey data showed a widespread unfamiliarity with both makerspace activities and 3D printing before engaging in the course activity. After completing the activity, survey data showed students were more comfortable using makerspace equipment, more interested in learning about other makerspace equipment, and demonstrated a general sentiment that 3D printing a prosthetic finger was *not* a technically difficult exercise. Students felt a sense of inclusion and belonging to a technological community (Bhatti et al. 2021). Both the seed dispersal activity and the 3D printing activity are critical to establishing effective teaming early in the course, ensuring that the subsequent team design projects are not the first time teammates engage with one another.

## Teaming process for design

Each team creates a Collaborative Plan (Supplement S9) for their final design project that leverages diversity to best define their individual and group goals, roles, processes, and relationships. Teams submit a scientific publication for inspiration, get approval from the graduate student instructors, get feedback on their original collaborative plan, and then submit a revised Collaborative Plan because effective collaboration must be iterative (Lau et al. 2012). To facilitate communication among team members and the class, we use our course management system (i.e., Canvas), which includes an online discussion forum. We give each team additional online space for sharing design ideas (i.e., collaborative Box folders). All 36 teams present their poster and 5-min video publicly in a Design Showcase at Jacobs Institute and online (i.e., Adobe Behance—the world's largest creative network for showcasing and discovering creative work).

## Aim 4: Connect to communities to see career paths

We have developed a structure fostering a community of learners which shifts each student's role from passive knowledge recipient to active co-constructor of knowledge responsible for their own learning (Fig. 1C, D; Dingyloudi and Strijbos 2020). To this end, students build a shared database of discoveries, classes, organizations, role models, next step programs, teach a course by designing their own curriculum, and join a student-created organization. Our program is best described as a **student-centered creative action community**. Our aim is to have students see new career paths outside of academia, participate in research, public service, community organizations, internships, entrepreneurship, and industry to become members of communities of practice (Wenger 1998).

## Connections by a student-created, custom database

**Fig. 3:**
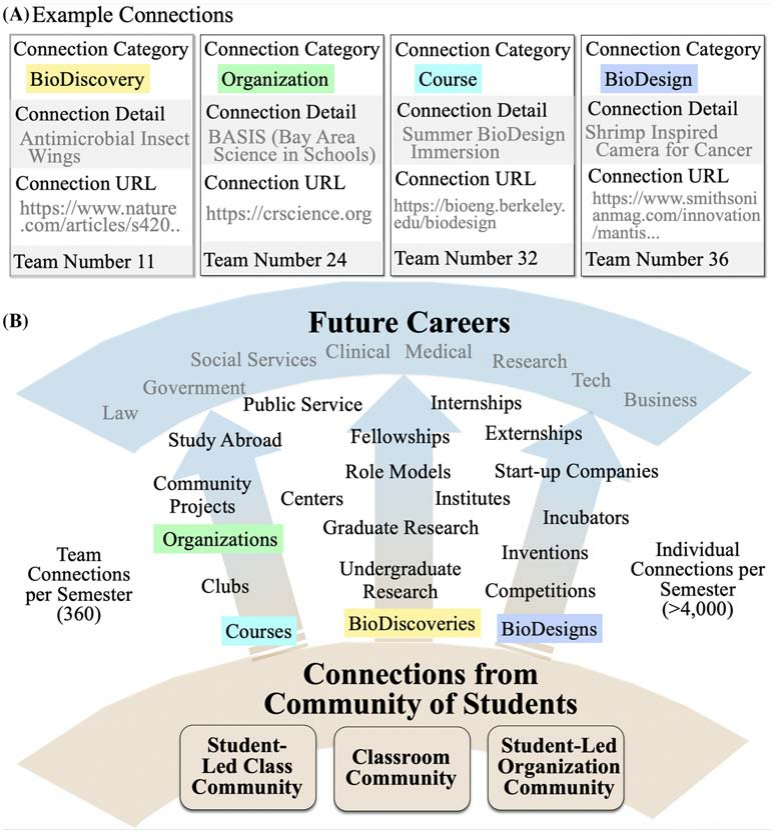
Student-created shared database of Connections (URLs). (**A**) Example connections include the Category, detail description, the URL, and the Team number submitting. Example Format: Team #24: Organization, Local: https://crscience.org. (**B**) Categories of Connections shared from our communities where students see paths to future careers. Colored boxes represent different Connection categories.

To build a student course community who sees new paths to future careers, student teams create a shared database of Connections. A Connection is a URL that points to a website (Fig. 3A; Supplement S10). URL categories range from local to global and can include: (1) publications of biological discoveries and bioinspired designs; (2) research laboratories, centers, institutes, foundations, and companies where discoveries are made and translated; (3) investigators demonstrating diverse paths to success; (4) organizations to join such as professional and URM research societies, clubs, support groups, and competitions; (5) relevant courses to take on campus and online; and (6) labs to conduct undergraduate research, potential graduate and medical degree programs, and business internships. Bioinspired Design is a field so well-suited to generate a connections database because it is one of the world's most rapidly changing interdisciplinary fields. Novel principles are discovered daily in concert with new designs. The rate of doubling for bioinspired design journal publications and conference papers is every 2–3 years compared to 13 years for most scientific fields (Lepora et al. 2013). Students submit Connections stimulated by the field of bioinspired design that allow them to see both immediate and long-term opportunities toward career paths far beyond the field (Fig. 3B). Students become energized by sharing general categories of engagement, participation, and career paths meaningful to them. Teams submit a Connection before each lecture using an online form that requires a URL, the Connection category selected, and team number (Fig. 3A). After reviewing all submitted Connections, we encourage the public and class-wide dissemination of ideas. At the beginning of a lecture, three design teams enthusiastically share their Connections. Additionally, individual students have one week to scan the team Connections and select those of personal interest through a “Like” button embedded into each Connection entry. This simple exercise allows students to find inspiring, crowd-sourced career directions based on submissions by the classroom community-at-large (Freeman 2012; Fig. 3B).

## Connections by a student-led class

Students today demand a voice in the direction of STEM to avoid being confined to paths set by previous generations. Given exponential rates of societal, technological, and environmental change, it is no longer sufficient to provide students with only foundational content knowledge to address current global problems. We must prepare students to embrace change as an opportunity to define their futures (Dorph et al. 2016). We must showcase the advantages that arise from engaging diverse minds. We have a program (i.e., Democratic Education at Cal or DeCal) of student-run courses allowing students to create and facilitate their own small classes (Fig. 1C). DeCal classes are recognized university classes run by students with a faculty sponsor. Students offer two versions of the student-led bioinspired design DeCal course. The first version focuses on more foundational material for those who were unable to take the large-scale Bioinspired Design course. Student instructors who have taken the large-scale class set their own syllabus, emphasis, lectures, such as bioethics, design projects, and events. Additionally, student instructors use the class to pilot new team and design exercises for which they see value (e.g., 3D printed child's prosthetic hand). The second version enables students to build upon their design work from previous classes. They move their design to protype stage, approach needed researchers, and then consider incubators and start-ups. Amazingly, through this student-led structure, one team reached the finals in the International BioDesign Challenge with a project on a hornet's silk to develop a biodegradable, thermoelectric, and thermoregulatory textile that can be used to power emergency shelters. The team used the NSF I-corps program and applied it to accelerator programs (e.g., YCombinator and IndieBio).

## Connections by a student-led, community organization

To provide a sense of community and belonging to our extended campus community, students founded a registered student organization, Berkeley Biodesign Community—BioD, (Fig. 1D) in association with our Leadership, Engagement, Advising, and Development Center (LEAD Center). The community can access campus resources that include funding opportunities, and event, facilities, and insurance support. Students wrote a constitution and selected a President, Projects Coordinator, Secretary, and Education, Events, Outreach, and Finance Coordinators. Members join from the Bioinspired Design class, the DeCal classes, other campus organizations, and from the diverse campus community. The community organization creates novel design projects, provides makerspace training, holds design workshops, orchestrates design-a-thons, and schedules tours, field trips, collaborates with other student organizations, and recruits underrepresented students. Students have new opportunities to connect with U.C. Berkeley's public service office, undergraduate research, career center, internships, and our entrepreneurship and innovation centers. We want to emphasize that our program is not limited to the foundational, large-scale course. After our foundational course ends, the engagement opportunities do not. Students can continue involvement by participating in our student-led classes and organizations, all of which lead to a sense of belonging in a creative action community.

## Aim 5: Participation beyond our own campus

Through dissemination efforts, students beyond U.C. Berkeley's campus are participating by way of other universities, symposia and professional development sessions, and K-12 Summer Camps.

## Sharing bioinspired design lectures, laboratories, assignments, and assessments

We plan to continue to share our program materials with other universities and colleges. We have developed a complete online master manual containing all aspects of the syllabus, announcements, assignments, lecture slides, personal response system questions, demonstrations, laboratory handouts and materials, discussion presentations, teaming materials, surveys, and all assessment exams, rubrics, and self-reports. Thus far, colleagues have developed similar bioinspired design programs utilizing our dissemination materials at the University of Nebraska, Omaha, the University of Michigan, and the University of Colorado, Boulder.

## Sharing by symposia and professional development sessions

We share our program through symposia presentations associated with biological professional societies (e.g., the Society of Integrative and Comparative Biology), education and social science societies (e.g., Understanding Interventions; Flores et al. 2021), and professional development sessions at underrepresented student research societies (e.g., Annual Biomedical Research Conference for Minority Students; Full 2019, and the Society for Advancement of Chicanos/Hispanics and Native Americans in Science).

## Outreach — K-12 summer camps

Partnering with our public education research center (i.e., Berkeley's Lawrence Hall of Science), we offer a week-long Bioinspired Design summer camp for local and international high school students. Using the science learning activation approach (Dorph et al. 2016), students receive an introduction to bioinspired design while participating in teaming exercises (e.g., seed dispersal activity, 3D printing child's prosthetic hand), team design activities (e.g., gecko adhesive and origami robot), tours of U.C. Berkeley's research museums, botanical garden, and makerspace design institute, and complete each day with a reflection/discussion. On the last day, student teams create their own bioinspired design and present them to the class.

## Aim 6: Own the program through feedback

**Fig. 4:**
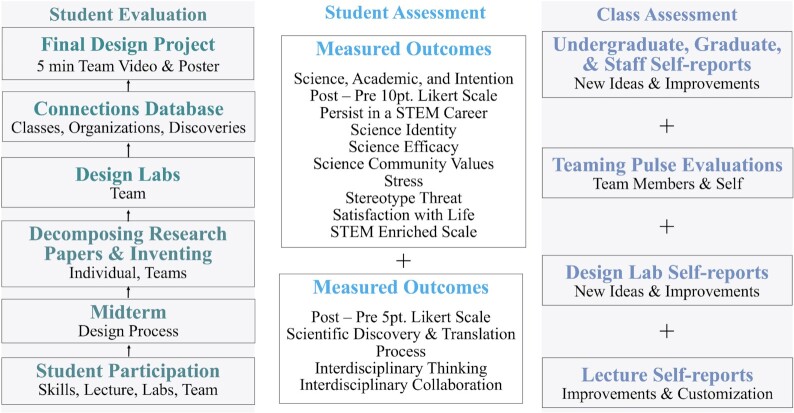
Bioinspired design class evaluations (left-hand column), student assessments (middle column), and class assessments (right-hand column). Assessment data are collected through a series of surveys (self-reports).

We act on the valuable feedback we gain from using class evaluations, class assessments of activities, and student skills and outcomes assessments. This feedback is critical to informing our instruction and advances our pedagogy (Fig. 4).

## Student evaluations

### Lecture — every student raises their hand for every lecture

We imagined a large-scale STEM classroom in which every student raises their hand for every lecture, a classroom in which students’ voices matter and are heard. To this end, we implemented an activity in which students view the lecture in-person or online and answer two included questions. Students completed a “Reflection Question” in which they record a single concept and/or principle that was new to them and caught their interest. They explain it as a text submission assignment. From this, we learn about students’ interests and whether they understood key concepts. Such an approach facilitates formative assessment and the opportunity for instructional adjustments. More importantly, every student has a voice that is valuable and heard. Students also submitted a “Lecture Question" in which they ask one question they have about the lecture. Their question can range from a clarification or re-explanation to one that goes beyond the material to explore its ramifications. We track questions from every student for each lecture in a database. The questions are then sorted by discussion section so that the most common questions can be used to drive team activities and be discussed amongst all students in the section. Moreover, the responses give the instructor unprecedented insight into the thinking of a student. This personal connection is especially useful prior to any one-on-one meeting during office hours or if an instructor wants to learn more about a student who may be struggling.

### Midterm “Exam” — show what you know

Students’ exams cover material from the lectures, readings, and discussions (Supplement S11). Exams are not the typical timed, proctored evaluations. To reduce stress and promote more equitable evaluation practices exams have a 14-day window for completion. We ask every student to select a course-relevant scientific publication from a range of biology journal suggestions and then decompose the paper's discovery, use an analogy check to suggest an invention, and name a start-up company. We use situated learning (Lave and Wenger 1991) by telling students that they are part of a design team that includes a biologist, engineer, designer, and a start-up company with entrepreneurs and business managers. We ask them to take the role of the author of the biology paper. We have them answer the team's questions based on the publication selected, proposed design invention, and bioinspired design process lectures (Fig. 2A, B, bottom). We ask them to explain one relevant concept or principle from each lecture to their design team. This is truly a “show what you know” exam, not what you forgot. Our method encourages students to apply class principles to an authentic discovery in a creative context that interests them most.

### Individual and team design assignments

Students and teams are evaluated with rubrics for assignment completion. Supportive qualitative feedback (Sadler 1989; Mulliner and Tucker 2017) is given by graduate student instructors to increase self-efficacy, especially for the initial individual assignments where students are learning the bioinspired design process. Students receive detailed comments on each submitted assignment, rather than just a quantitative score. In addition, we assess each project using a creativity rubric which we developed and plan to present in a future publication.

### Connections submissions

Students submit both team and individual submissions once a week (Fig. 3A). We have three teams present their Connection at the beginning of class to encourage others to look at and “Like” it. We see how often connections lead to project ideas. We use student feedback of their interests to improve all aspects of the program. In the future, we plan to better organize and offer avenues to follow-up on connection opportunities by developing a Connections Hub.

### Final design projects

Student teams submit a final project invention, poster, and 5-min video. One month before final submissions, teams select a research paper containing a benchmark biological discovery and upload it for approval from the instructional staff. Teams do a Discovery Decomposition and Analogy Check (See Supplement S2). Teams propose and then create a novel bioinspired design using photographs, diagrams/blue-prints, CAD drawings, simulations and/or videos, craft supplies, and 3D-printed and laser cut pieces (Supplement S5). Teams are asked to describe the next steps if they follow up on their design, who they would collaborate with, what critical pieces of information are needed, and the likely major roadblocks. Teams include possible societal impact (e.g., health, fitness, sports and entertainment, environment, safety, security, education, connections to others or communities, assisting underserved, disabled populations, or underdeveloped countries). Teams conclude their poster and video with a pitch to a government organization, venture capitalists, an established company, or propose steps to creating their own start-up. Teams present their poster and video at a Design Showcase open to the public and online. We evaluate the projects using rubrics for completion, effort, quality, and include a comprehensive assessment of creativity which has been presented at a meeting (Flores et al. 2021) and is being prepared for publication.

## Assessment of class activities

We ask graduate instructors and staff for self-reports of improvements and new ideas at the semester's end. We provide students the opportunity to assess the lectures, the design laboratories, and their teaming experience and teammates (Supplements S12–15). We ask students what was most and least effective. We value their voice and request specific ideas for improvement. We make every effort to have students own the course through our actions taken based on their self-reports. Perhaps our most notable example thus far was addressing a major challenge where students did not feel that they belonged to a technological community because the course had no early makerspace activities. In our introductory student-led class (DeCal), we piloted each student 3D printing a finger of a prosthetic hand designed for children (see Teaming Activities, Aim 3). After gathering feedback from the pilot activity, we made modifications and introduced the exercise into the second week of the large-scale class. After this requested change, assessment showed a profound increase in belonging and connection (Bhatti et al. 2021).

## Assessment of student skills and outcomes

One large-scale course goal focuses on the development of 21^st^ century skills (see Aim 7). We have conducted a pre-, post-survey for 21^st^ century skills for the last five years (Supplement S16). We present a preliminary analysis of the 21^st^ century skills self-reports here (Supplement S17). The second major course goal focuses on assessing persistence in the scientific community. We have completed pre-, post-surveys for persistence (integration) over the last three years (Supplement S16). To assess intentions to persist in a STEM career, we examined science identity, science self-efficacy, science community values, stress, stereotype threat, well-being (based on Estrada et al. 2011), and a new STEM enriched scale created for this course. We share the psychosocial variables survey here (Supplement S16), but will publish the complete analysis in a future paper. Our third goal is to assess the effect of diversity on team project creativity. Thus far, we have presented an abstract with preliminary data (Flores et al. 2021) and will follow with a publication.

## Programmatic assessment of student-led classes and organizations

We are developing programmatic assessment of all the Creative Action Community components. We started by giving surveys in the student-led class and organization. Unfortunately, the disruptions associated with the pandemic slowed this process immeasurably.

## Aim 7: Develop 21^st^ century skills

Numerous 21^st^ century skills have been proposed as critical education outcomes (Care 2018; Trilling and Fadel 2009; NRC 2012). These include critical thinking and problem solving, creativity and innovation, collaboration, teamwork, and leadership, cross-cultural understanding, communications, information, and media literacy, computing and technology literacy, and career learning of self-reliance (Trilling and Fadel 2009). Our program attempts to address many of these skills, but here we assess the skills we consider critical to succeeding in our course and remaining applicable to all transformative STEM courses; scientific discovery and the translation process, interdisciplinary thinking, and interdisciplinary collaboration.

## Assessing 21^st^ century skills

The foundation for our formal assessment tools is the BEAR Assessment System (BAS) created by Wilson (2005). BAS is an integrated approach to developing assessments that provides meaningful interpretations of student work relative to the cognitive and developmental goals of a curriculum (Wilson and Sloan 2000). The BAS uses four building blocks of assessment (Wilson 2005) that include a construct map, items design, outcome space, and measurement model (Supplement S16; Fig. 1S). The first building block (Construct Map) concretely identifies variables, described as capabilities, approaches, attitudes, and skills that can be observed to assess whether students are meeting goals. We assign five levels of development or success to a given Construct—from Novice to Expert. We specify the data necessary to demonstrate each level of success for three main areas of our 21^st^ century skills construct. We created the second building block (Item Response) in the form of a survey directly aligned to the Construct Map areas. For the third building block, we generated a scoring guide (Outcome Space). These guides are rubrics translating responses from our surveys (ratings on Likert-type items) into quantitative data or scores. Our fourth building block relates the survey scores to our development levels (measurement or interpretational model). We analyzed the responses to the survey items using item response theory (IRT) guided by the Construct Map (Hambleton et al. 1991). An IRT partial credit model was applied to the survey data to give statistical evidence that the assessment was reliable and the steps in the response scale (e.g., strongly disagree to strongly agree) were ordered (Wright and Masters 1982).

## Results from 21^st^ century skill assessment

From 2016 to 2020, we administered a 26-item Likert-type pre/post course survey as a self-reported measure of students’ 21^st^ century skills (Supplement S17), resulting in 514 pre- and 432 post-survey responses. Our preliminary analysis of pre/post changes in raw Likert scores showed increases in “agreeability” for all items, including those mapped to the highest levels of the construct map (i.e., items that we considered “hardest” to agree with). Thus, the mean score for each item on the survey increased from pre to post, resulting in a positive delta value for each item. Students showed growth in all skills and subdimensions of our 21^st^ century skills construct every year after completing the course. Considering the limitations of analyzing raw scores on self-reported Likert instruments (Chimi and Russell 2009), we plan to present a more in-depth IRT analysis of these data in a future publication.

## Author's contributions

R.J.F., H.A.B, and P.J. contributed to the writing of the manuscript. R.J.F,  J.M., and P.J. developed the course/discussion approaches and materials. R.J.F,  H.A.B,  R.R., P.J., and T.J. implemented and facilitated the student-led class and community. H.A.B, R.R., and T.J. developed team activities. R.J.F,  H.A.B, L.F., J.M., and M.E. developed the assessment.

## Supplementary Material

icab187_Supplemental_Files

## Data Availability

The data underlying this article are available in the article and Supplementary Appendix.

## Notes

From the symposium “Biology Beyond the Classroom: Experiential Learning through Authentic Research, Design and Community Engagement” presented at the annual meeting of the Society of Integrative and Comparative Biology Annual Meeting and Exhibition Final Program and Abstracts. Washington, DC. January 3–February 28.
