# Supplementary material for: Eyes Toward Tomorrow Program Enhancing Collaboration, Connections, and Community Using Bioinspired Design
Source: Integr Comp Biol. 2021 Aug 30;61(5):1966–80. doi: 10.1093/icb/icab187 (PMC8699102; doi:10.1093/icb/icab187)
Supplement: icab187_Supplemental_Files [file icab187_supplemental_files.zip › icb-2021-0200-File006.pdf]

Supplement S1 – Biological Foundation Lecture Descriptions

Bioinspired Design Process

BioDiscovery. We begin by exciting students with the most cited examples of biomimicry (Mueller, 2008). Some are excellent representatives of the process, whereas others are not. We explain this by presenting the two approaches by which bioinspired design is accomplished (Helms et al., 2009). Because we focus on discovery, we use solution-driven biological inspiration that begins with a biological discovery, extracts the fundamental principle, and then creates an analogy to use this principle to solve a human problem. In practice, the solution-driven approach is complemented by the problem-driven approach where one begins with a human problem, searches the literature for strategies where biology has "solved" the problem, abstracts the principle, and generates a new design. Many of the successful approaches and programs originating from engineering or design focus on a problem-driven approach (Fayemi et al., 2017; Lenau et al., 2018; Qureshi, 2020; Stevens et al., 2020; Nagel et al., 2019), but more shared frameworks with biology are emerging (Yen et al., 2014; Wanieck et al., 2020; Graeff et al., 2020, 2021; Hashemi, 2020). In reality, it is the interplay between these two approaches where biologists are included that leads to both exciting discoveries and novel designs.

We explain the value of *direct experiments* which have treatments and controls. Direct experiments can establish cause and effect but disrupt the natural system. *Natural or comparative experiments* have treatments by way of evolution. Natural experiments lack some control because other features could have evolved, but do not disrupt the natural system. Discovery often balances experimentation with theory, so teams of investigators can enhance progress. We explain that significant advancements are often incrementally made over the years requiring many discoveries and investigators. Biological discoveries involve experts in biology making rigorous measurements, not just in laboratories, but also in the field. Biological discoveries and their translation are often conducted by investigators from different disciplines working together. Discoveries in one discipline can lead to advances in a collaborator's field. The collective discoveries of interdisciplinary collaborations can be beyond any single discipline. Engagement remains high because the collaboration solves the team's common problem through directly benefiting your discipline. We term this *mutualistic teaming*.

BioDesign. To present our approach to bioinspired design, we begin by using our first short publication on hairy-toed gecko adhesion (Autumn et al., 2000). Original research papers are how a disciplinary expert communicates with other experts in their field (Hubbard, 2021). We should not expect undergraduates, especially from diverse disciplines, to understand every word of a research paper. There are a host of pedagogical strategies available to assist novice readers with disciplinary texts and research papers, such as writing abstracts, using key sentences, and learning the CREATE strategy (Hoskins et al., 2011; Hubbard, 2021). We developed a simple method, *Discovery Decomposition*, to understand a scientific publication (See Supplement S2). Using a flow chart format, we see students with no science background navigating the maze of jargon and data in scientific papers by mapping what was known, done, measured and discovered, to come away with the biological principle they can use for design.

Once students grasp a scientific principle that excites them, they learn how translating it into a design is actually an analogy derived from informal logic. Many abstraction tools and methods have been developed depending on the discipline of the instructors and students. These include abstracting design principles' of a biomimicry template, checklist of key questions, CK modelling, DANE structured descriptions, functional decomposition, function-means trees, SaPPhlRE model of causality, structure-behavior-function, structured representation using the four box method, TRIZ/ BioTRIZ, and what-why-how templates (See Table 2 in Rovalo et al., 2020). Our simple *Analogy Check* process ensures students identify the similarities and differences present in the organism compared to their proposed design related to structure, size, operating environment, mechanism, specification, performance, and constraints (See Supplement S2). Initially, mismatches lead to uncertainty and ambiguity with respect to translation. Instead, we explain that these are the very differences that lead to exciting, new research publications and

projects. Eventually, differences lead students to propose hypotheses generating future research projects to see how they could take the next step in the discovery process. “Better Designs” from Nature can be cutting-edge, novel designs that outperform all current human technological advancements. They can lead to new disruptive industries with a societal impact by being sustainable, with low-cost materials, less waste, energy savings, and restoration of function. This promise of bioinspired designs demands conservation, because if we do not preserve organisms and our environment, their secrets will be lost forever. Contrary to expectation, students see that the design process is not a one-way street but represents a synergy. The discovery of principles and analogies from biology that are advantageous are also useful to engineering, mathematics, computer science, and art/architecture. Nature’s inspiration can then be integrated with best human efforts to design something potentially even better than Nature. Using bioinspired principles combined with human technology allows us to go where Nature has not. Biologists can use ideas, approaches, and devices from interdisciplinary collaborations to accelerate biological hypothesis generation and realize the next novel measurement and discovery.

BioConstraints. Many students, engineers, and bioinspired design courses assume Nature’s creatures are optimally designed by evolution and should be copied or mimicked. We shatter these myths through lectures detailing how evolution is not engineering and directly debunk intelligent design. Evolution often fails to keep pace with the rate of environmental change. Today's adaptation is tomorrow's constraint. Specialization is often an evolutionary dead end. Biological evolution works more as a tinkerer than an engineer. Tinkerers never really know what they will produce and use everything at their disposal to make something workable (Jacob, 1997). Organisms are severely constrained by their development, evolutionary history, multifunctionality, and sexual selection (Dudley and Gans, 1991; Fish and Beneski, 2014). Analogies that effectively translate biological principles to designs, must recognize and remove these confounding constraints. In 1856, pondering the brutal inefficiency of natural selection, Darwin wrote to his friend Joseph Hooker, "What a book a devil's chaplain might write on the clumsy, wasteful, blundering, low, and horribly cruel works of nature!" (Coyne, 2003). After 3.8 billion years of evolution, organisms are designed to be just good enough. Organisms carry the baggage of their past and are blind to future changes. Yet, Vogel (2000) reminds us that Nature's technology represents the only known alternative to our own. Looking at Nature's designs liberates us from the constraints of our own habits, history, and outlook. Given the richness of evolution, biologists are needed to check analogies for constraints, engineers and architects to construct the design, and designers, artists, and social scientists to ensure consideration of human factors and social needs.

BioScaling. The sizes of both natural and human technologies vary across twelve orders of magnitude, making the translation of a bioinspired principle difficult (McMahon, 1983). Students must consider size to develop the most effective analogies. One of the most common reasons translations of a biological principles fail are due to significant differences in size. Fortunately, students learn that the geometry they learned in high school gives a foundation for comparing organisms and their parts that differ in size (e.g., geometric similarity). We provide them with the mathematics for size correction. Relative to the size of other organisms, humans are enormous, so our understanding of biological size is skewed. Humans tend to start with large technologies that they scale down, whereas nature tends to start with small, stand-alone components that it assembles into larger structures. Historically, the smallest natural technologies have been much smaller than the smallest human technologies, and the largest human technologies have been much larger than the largest natural technologies. However, micro- and nanotechnology have enabled human technologies to approach the sizes of the smallest natural technologies. As human technologies take on more of the characteristics of nature, nature becomes a more useful teacher (Vogel, 2000).

BioComplexity. In comparison to most human technology, organisms are incredibly complex. Even with complete knowledge of all lower-level components, one cannot predict organ, organismal, or group behavior. Complexity makes extracting and translating biological principles to devices challenging.

Simple models help biologists explore complex systems. Models can be used to test hypotheses, lead to the development of conceptual frameworks, make accurate predictions, and generate explanations. As George Box (1976) said, "Every model is wrong; some models are useful." Useful models lead to unexpected outcomes that can be verified, are often created in hindsight to explain diverse observations, are adapted to design materials or processes, are used to express data, reveal patterns, or for implementation in control algorithms. Both mathematical and physical models serve as hypotheses for biological discovery. We encourage simple mathematical models that we call templates anchored in a sufficiently representative model to test a hypothesis (Full and Koditschek, 1999). Physical models uncover unknown and important parameters missed in mathematical models because physical models meet the real environment (Koehl, 2003). Physical models provide starting points when no mathematical models exist, allow novel experiments not easily modeled mathematically, allow for the study of extinct or endangered species, and can be the first step in producing prototypes for applications. By encouraging students to consider how their prototype might also serve as a hypothesis to test ideas in biology, we begin to show the synergy in action.

BioSelection. Selecting an organism for inspiration is a challenge. Students realize that literature searches or database use, even with artificial intelligence, that is disconnected from the biological experts who make the original discoveries will be insufficient. Selection best exemplifies our program mantra **Diversity Enables Discovery**. Students discover that they can select by exceptional performance, learn from convergent evolution, examine trends from evolutionary history, take advantage of model organisms, be guided by general relationships, and/or use extreme or unique solutions.

Additional Supplement References

Autumn, K. et al., 2000. Adhesive force of a single gecko foot-hair. Nature, 405(6787), pp.681.

Box GE. Science and statistics. Journal of the American Statistical Association. 1976 Dec 1;71(356):791-9.

Coyne, Jerry A. "Gould and God." Nature 422.6934 (2003): 813-814.

Dudley, Robert, and Carl Gans. "A critique of symmorphosis and optimality models in physiology." Physiological Zoology64.3 (1991): 627-637.

Fayemi PE, Wanieck K, Zollfrank C, Maranzana N, Aoussat A. Biomimetics: process, tools and practice. Bioinspiration & biomimetics. 2017 Jan 23;12(1):011002.

Fish FE, Beneski JT. Evolution and bio-inspired design: natural limitations. In Biologically inspired design 2014 (pp. 287-312). Springer, London.

Full R.J., Koditschek D.E. Templates and anchors: neuromechanical hypotheses of legged locomotion on land. Journal of experimental biology. 1999 Dec 1;202(23):3325-32.

Hashemi Farzaneh H. Bio-inspired design: the impact of collaboration between engineers and biologists on analogical transfer and ideation. Research in Engineering Design. 2020 Jul 1;31(3).

Hoskins SG, Lopatto D, Stevens LM. The CREATE approach to primary literature shifts undergraduates' self-assessed ability to read and analyze journal articles, attitudes about science, and epistemological beliefs. CBE—Life Sciences Education. 2011 Dec;10(4):368-78.

Hubbard K. Disciplinary literacies in STEM: what do undergraduates read, how do they read it, and can we teach scientific reading more effectively? Higher Education Pedagogies. 2021 Jan 1;6(1):41-65.

Jacob, F. 1997. Evolution and tinkering. Science. 196(4295): 1161–1166.

Koehl MA. Physical modelling in biomechanics. Philosophical Transactions of the Royal Society of London. Series B: Biological Sciences. 2003 Sep 29;358(1437):1589-96.

McMahon TA. On Size and Life (Scientific American Library, New York, 1983); JT Bonner, The Evolution of Complexity by Means of Natural Selection.

Mueller T. Biomimetics: Design by nature. National Geographic. 2008 Apr;213(4):68-91.

Qureshi S. How students engage in biomimicry. Journal of Biological Education. 2020 Dec 2:1-5.

Stevens L, Kopnina H, Mulder K, De Vries M. Biomimicry design thinking education: a base-line exercise in preconceptions of biological analogies. International Journal of Technology and Design Education. 2020 Mar 9:1-8.

Vogel, Steven. Cats' paws and catapults: Mechanical worlds of nature and people. WW Norton & Company, 2000.

Wanieck K, Ritzinger D, Zollfrank C, Jacobs S. Biomimetics: teaching the tools of the trade. FEBS Open bio. 2020 Nov;10(11):2250-67.

Yen J, Helms M, Goel A, Tovey C, Weissburg M. Adaptive evolution of teaching practices in biologically inspired design. In Biologically inspired design 2014 (pp. 153-199). Springer, London.

Supplement S2 – Discovery Decomposition and Analogy Check- Individual Assignment

Assigned: January 31

Due: February 6, 6 PM. Please upload as a pdf file

Discovering the Mechanism of How Hairy-footed Geckos Stick and Checking the Analogy of Your Proposed Design

Read the research publication: Geckos#2_PNAS.pdf

Autumn, Kellar, Metin Sitti, Yiching A. Liang, Anne M. Peattie, Wendy R. Hansen, Simon Sponberg, Thomas W. Kenny, Ronald Fearing, Jacob N. Israelachvili, and Robert J. Full. "Evidence for van der Waals adhesion in gecko setae." *Proceedings of the National Academy of Sciences* 99, no. 19 (2002): 12252-12256.

Each student will create and hand-in:

1. **Discovery Decomposition**. Break down the second gecko publication listed above using the Discovery Decomposition approach discussed in class and lecture (examples below). Produce your own flow diagram using the structure below as we did for the first gecko publication. Use the following file as a template to get you started (edit the number of boxes and arrows as you see fit.) Use Discovery_decomp_v1.pptx as template.

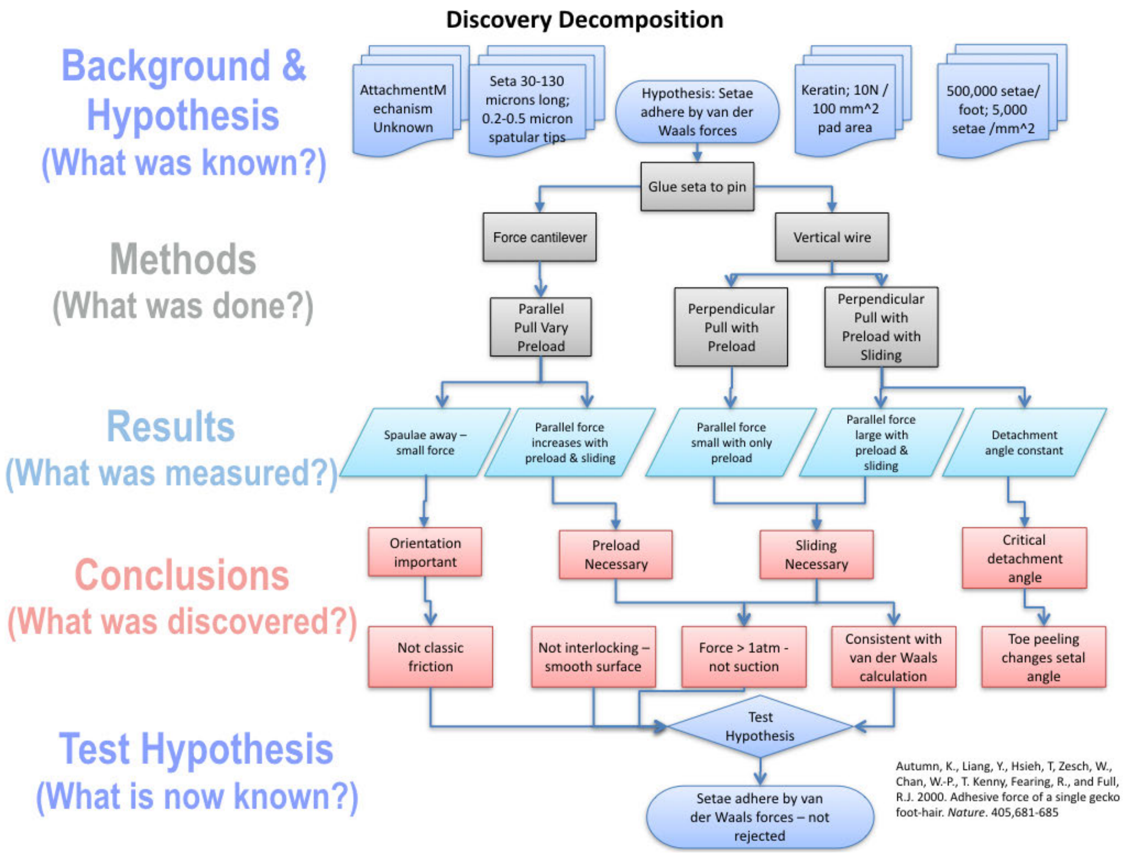

2. **Analogy Check.** Create an Analogy Check Table for the second gecko publication listed above using the approach discussed in class and lecture. Produce your table using the structure below as we did for the first gecko publication and suggest a novel design and invention name. In the first column, fill in as much as possible from what you have learned from the second gecko publication Discovery Decomposition. In the second column, Design Problem, make sure you fill out your best guess about your new design. In the middle column indicate whether the parameters are Similar (translation possible, green for go), Different (need more experimentation, red for stop), Uncertain (need more information, gray) or N/A (not applicable). Use the following file as a template Analogy_check_v1.xlsx.

Design Solution	Analogy Check	Design Problem
**Gecko (*Gekko gecko*)**		**Invention Name**
**Function**<br>(What does system or organism do?)		**Function**<br>(What do you want system to do?)
**Structural Components**<br>(What is structure or organization of system?)		**Structural Components**<br>(What can the structure be?)
**Operating Environment**<br>(Where?)		**Operating Environment**<br>(Where?)
**Size**<br>(What is size?)		**Size**<br>(What size needed?)
**Mechanisms**<br>(How does system work?)		**Mechanisms**<br>(How do you want the system to work?)
**Characteristics/Specification**<br>(Which are distinguishing?)		**Characteristics/Specification**<br>(What are your specifications?)
**Performance Criteria**<br>(How well does system work?)		**Performance Criteria**<br>(How well must the system work?)
**Constraints**<br>(What compromises system?)		**Constraints**<br>(Can compromises be removed?)

Example for class. Jayaram K, Full RJ. Cockroaches traverse crevices, crawl rapidly in confined spaces, and inspire a soft, legged robot. Proceedings of the National Academy of Sciences. 2016 Feb 23;113(8):E950-7.

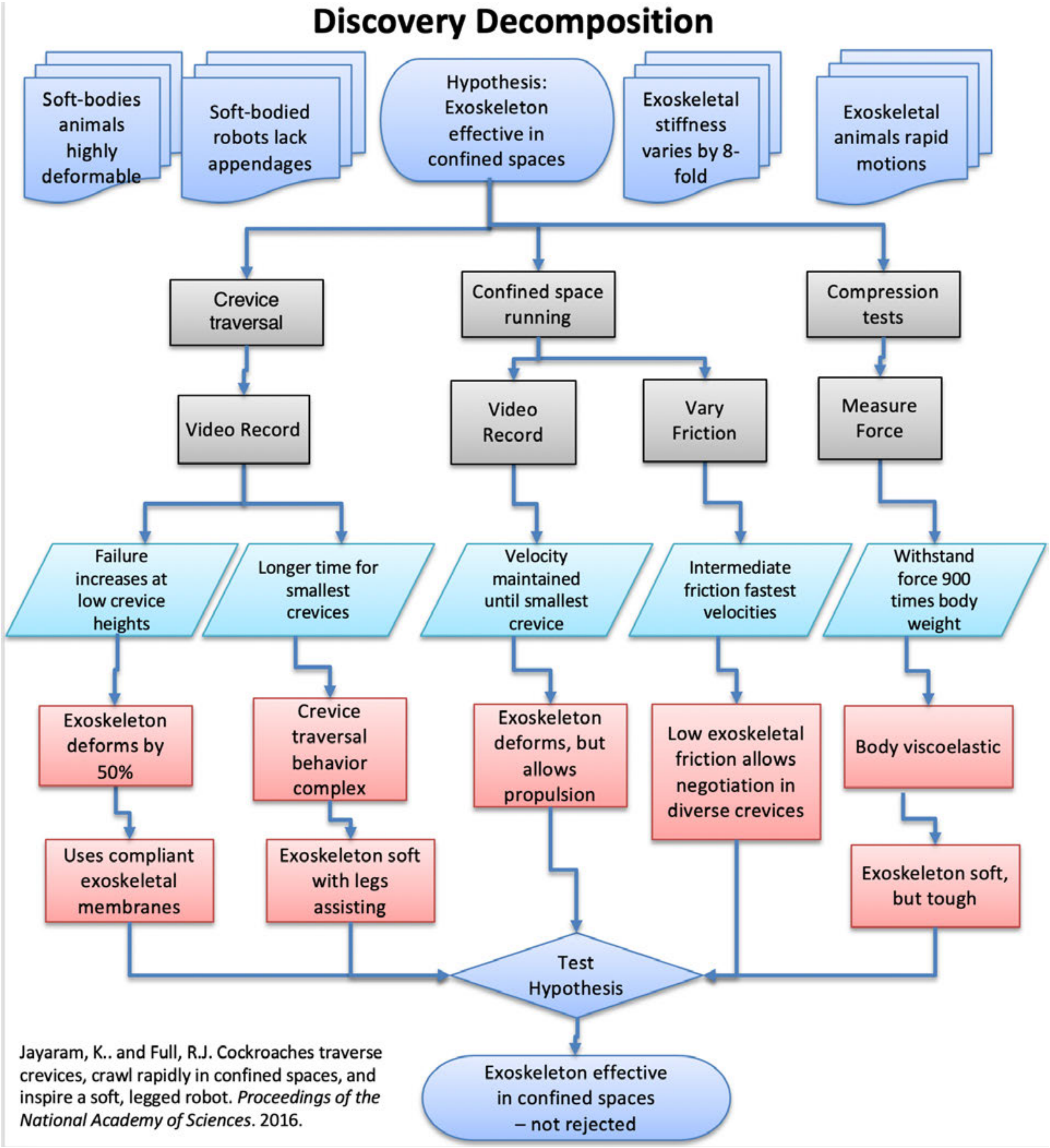

Design Solution	Analogy Check	Design Problem
**Cockroach** (*Periplaneta americana)*		**Search-and-Rescue Robot**
**Behaviors** (What does system or organism do?)		**Behaviors** (What do you want system to do?)
Crevice traversal	Similar	Crevice traversal
Rapid confined space locomotion	Similar	Rapid confined space locomotion
**Structural Components** (What is structure or organization of system?)		**Structural Components** (What can the structure be?)
Chitin	Different	Cardboard
Compliant membranes (arthrodial)	Uncertain	Polyester
Stiff plates and tubes	Uncertain	Cardboard
**Operating Environment** (Where?)		**Operating Environment** (Where?)
Crevice - Plexiglas	Uncertain	Crevice - rubble
Confined space (tunnel) - Plexiglas	Uncertain	Confined space (tunnel) - rubble
**Size** (What is size?)		**Size** (What size needed?)
Standing height (6mm)	Different	Standing height (75mm)
Compressed height (3mm)	Different	Compressed height (35mm)
**Functional Mechanisms** (How does system work?)		**Functional Mechanisms** (How do you want the system to work?)
Compresses by soft membranes	Uncertain	Compresses by polyester membranes
Propulsors (legs) assist	Uncertain	Propulsors (legs) assist
Body friction low	Uncertain	Body friction low with polyester
Viscoelastic, but tough	Uncertain	Viscoelastic, but tough
**Characteristics/Specification** (Which are distinguishing?)		**Characteristics/Specification** (What are your specifications?)
High compression	Similar	High compression
Robust to compression	Different	Far less robust to compression
**Performance Criteria** (How well does system work?)		**Performance Criteria** (How well must the system work?)
50% body compression	Similar	50% body compression
20 body lengths/s, confined space	Different	1 body lengths/s, confined space
800X body weight compression	Different	20X body weight compression
**Constraints** (What compromises system?)		**Constraints** (Can compromises be removed?)
Developmental - moulting	Uncertain	Fixed material
Multi-functional – many other behaviors	Uncertain	Single behavior
Evolution – chitin inherited from ancestors	Uncertain	Material can be varied
