# Supplementary material for: Eyes Toward Tomorrow Program Enhancing Collaboration, Connections, and Community Using Bioinspired Design
Source: Integr Comp Biol. 2021 Aug 30;61(5):1966–80. doi: 10.1093/icb/icab187 (PMC8699102; doi:10.1093/icb/icab187)
Supplement: icab187_Supplemental_Files [file icab187_supplemental_files.zip › icb-2021-0200-File007.pdf]

Supplement S3 - Gecko Inspired Adhesive Design Project

GECKO INSPIRED ADHESIVE DESIGN PROJECT

BACKGROUND

Designing with Soft Polymer Lamellar-like Arrays

If geckos had not evolved, it is possible that humans would never have invented adhesive nanostructures (Autumn and Gravish, 2008). Geckos use millions of adhesive setae on their toes to climb vertical surfaces at speeds of over 1 m s^{-1}. Climbing presents a significant challenge for an adhesive in requiring both strong attachment and easy rapid removal. Conventional pressure-sensitive adhesives (PSAs) are either strong and difficult to remove (e.g. duct tape) or weak and easy to remove (e.g. sticky notes). The gecko adhesive differs dramatically from conventional adhesives. Conventional PSAs are soft viscoelastic polymers that degrade, foul, self-adhere and attach accidentally to inappropriate surfaces. In contrast, gecko toes bear angled arrays of branched, hair-like setae formed from stiff, hydrophobic keratin (like your hair or finger nails) that act as a bed of angled springs with similar relative stiffness to that of PSAs. Setae are self-cleaning and maintain function for months during repeated use in dirty conditions. Setae function as an anisotropic (direction dependent) **'frictional adhesive'** (Autumn et al., 2006a,b) where adhesion force (perpendicular to the surface) requires a proximally directed (pulled to body) shear force (parallel to the surface).

The actual structure of the adhesive is hierarchical in nature and extends across the macro-, meso-, micro-, and even nanoscale. The whole hierarchical structure of a gecko, such as the Tokay, includes four feet, each foot with five toes, each toe with approximately 20 rows of lamellae (scansors), each lamella with many setal arrays consisting of thousands of setae, which amounts to approximately 200,000 setae per toe, and each seta consisting of hundreds to 1,000 spatulae at its end with sizes near 200 nm. Over the years since we discovered how geckos stick (Autumn et al, 2000, 2002), many investigators have shown that all levels of the hierarchy contribute to function, not just the nano-sized, spatular split-ends.

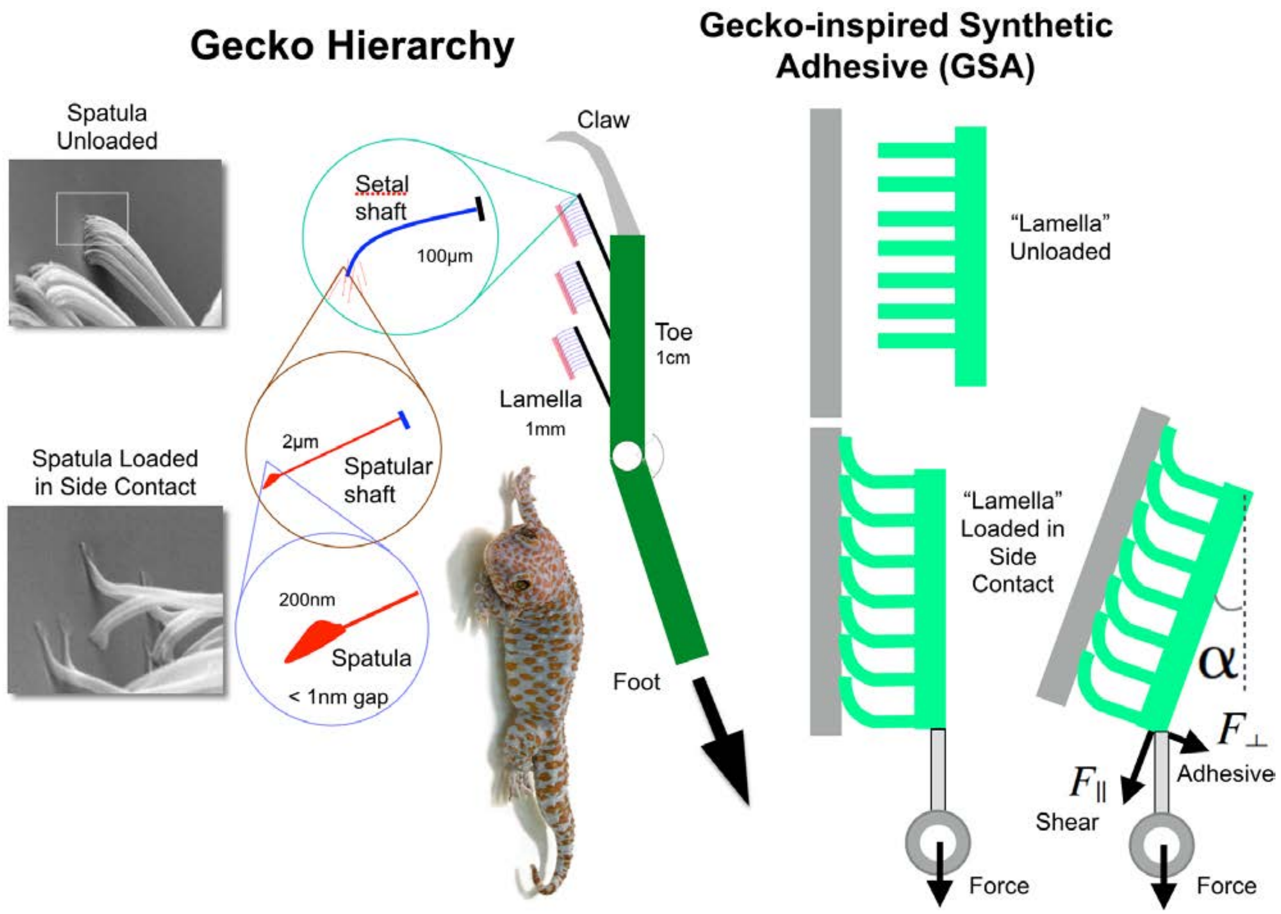

You will manufacture a Gecko-inspired Synthetic Adhesive (GSA) from polydimethyl siloxane (PDMS) that uses features at the level of the lamellae, but which function similarly to spatula with side contact. You will measure the forces resulting from side contact of your synthetic lamellae by hanging weights from the adhesive. Adhesive force ($F_\perp$, perpendicular to the surface) and shear force ($F_\parallel$, parallel to the surface) can be measured by changing the angle of attachment away from vertical (90°) as if the gecko was on the underside of an over-hang.

For more typical materials, friction or shear force ($F_\parallel$) equals μ (the coefficient of friction) times the normal load ($F_\perp$, perpendicular to the surface pushing down onto the surface).

$$F_\parallel = \mu \ F_\perp$$

When setae are dragged along their natural curvature, setae exhibit a response that

violates this law. As shear force is increased ($F_\parallel$), an adhesive force results ($-F_\perp$, perpendicular to the surface pulling away from the surface). It has been shown that the angle of the setal shaft to the surface (α) must be kept below 30° or detachment occurs. The requirement of shear force to maintain adhesion is an advantage because it provides precise control over adhesion via friction or shear force (Autumn et al. 2006), allowing strong attachment and easy removal.

Since we can resolve the force (F) along the setal shaft as follows:

$$F_\perp = mg\cdot\sin\alpha$$

$$F_\parallel = mg\cdot\cos\alpha$$

where m is mass and g is acceleration due to gravity (mg = the weight of the object you will hang from your adhesive, plus the weight of the adhesive itself), then:

$$F_\perp = F_\parallel\cdot\tan\alpha$$

As you add weight to your GSA sample, you will measure the angle at which your sample detaches and then calculate the shear ($F_\parallel$) and adhesive forces ($F_\perp$). Note your angle (α) may vary as you change weights, since you are testing lamellae.

FIRST PERIOD
OBJECTIVE - MANUFACTURING A GECKO INSPIRED ADHESIVE

In this design session you will:

1. Manufacture a gecko-inspired adhesive (GSA)
   - 1.1. Prepare the PDMS material
   - 1.2. Use a mold frame to form "lamellae", cure the adhesive, and then remove it from mold.
2. Estimate adhesive and shear force performance by hanging weights from your GSA and changing the attachment angle.
3. Write a short, 1page, report of your findings that will be included in your final report.

MATERIALS
For manufacturing your gecko-inspired adhesive, you will use:

1. A specially made mold with a frame.
2. A razor blade or exacto knife.

3. PDMS (body double from 'Smooth-on').
4. Weights for testing performance.
5. Apparatus for testing adhesive.

METHODS

To manufacture your gecko-inspired adhesive, you will:

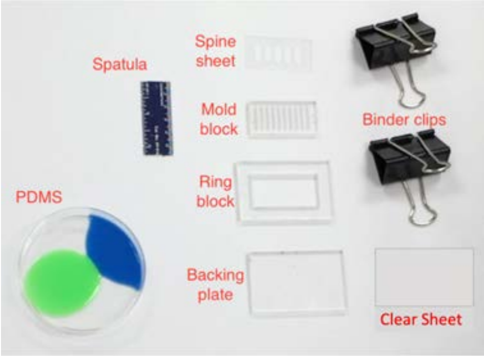

1. Watch the instructional video posted on bCourses
   Gecko_adhesive_instructions_compressed.mp4

2. Locate materials
   a. Get 4 grams of PDMS from a GSI
   b. Backing plate
   c. Clear sheet
   d. Ring block
   e. Binder clips
   f. Spatula
   g. Spine sheet
   h. Mold block

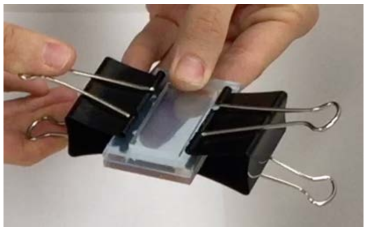

3. Prepare
   a. Stack the backing plate, blue sheet, and ring block (backing plate on bottom, ring block on top).
   b. Clamp the assembly on long sides with binder clips.

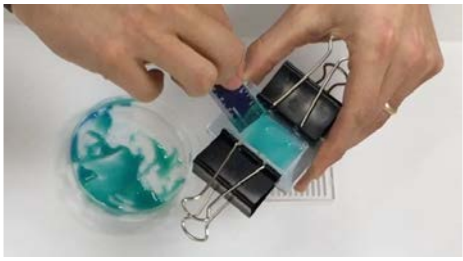

4. Mix and Mold (**FAST: total time < 1 minute 30 seconds**)
   a. Mix one color into the other with spatula until well combined (~ 15 seconds).
   b. **Spread about** ⅓ of the mixture onto the **blue sheet in** a uniform thickness.
   c. Place the **spine sheet** down into the well on top of the mixture.
   d. Spread the remaining mixture onto the spine sheet.
   e. Place the **mold block** into the hole with **offset pieces (little bumps) facing down.**
   f. Push the mold block slowly and firmly down, **pressing at the ends only**.
   g. Continue to press with medium pressure until PDMS oozes out of the slots in the mold block.
   h. Ensure that the mold block is level with the ring block.
   i. Wait 10 minutes for the PDMS to cure.

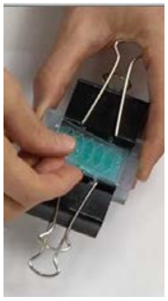

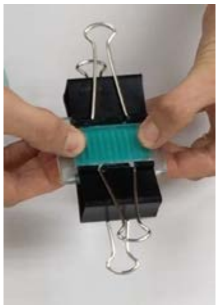

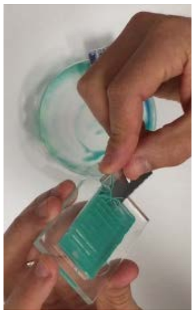

5. Remove the adhesive from the mold
    a. Remove the binder clips and gently peel the backing plate and blue sheet away from the ring block and mold block.
    b. Using a razor blade, trim off extra PDMS along the top and side of the mold. For the top, trim along the slots to remove excess PDMS.
    c. Ensure that **all excess PDMS has been trimmed from the top** (very important!).
    d. Push the adhesive and mold block out of the ring block.
    e. Trim excess PDMS around the edge of the mold block using a razor blade.
    f. Gently separate the mold block from the gecko adhesive by slowly working around the edges.
    g. Once edges are loosened, slowly peel from one end and watch the lamellae pop out of the slots in the mold block. Take care not to bend the spine to steeply or it will break.

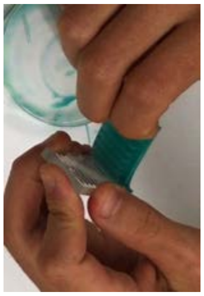

DATA COLLECTION AND ANALYSIS

To collect your gecko-inspired adhesive data, you will:

1. Vary load on your adhesive by hanging weights (W = mg) to the connected string.
2. For each weight, determine the angle of detachment (α).
3. Collect at least 3 samples for each weight.
4. Fill in data table below, and also enter measurements on a Google form: https://tinyurl.com/geckodata2020

Weight (N)	Angle (°) Sample 1	Angle (°) Sample 2	Angle (°) Sample 3
1.			
2.			
3.			
4.			

Before testing, clean the surface of a hanging glass slide

1. Squirt a small amount of isopropyl directly onto a wadded paper towel.
2. Rub the back side of the glass slide to clean it.

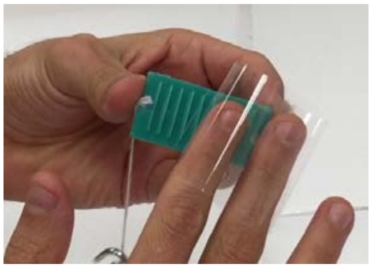

For every test you will do the following:

1. Prep the adhesive
    a. Clean adhesive with packing tape - use a short loop of reversed tape to tap the surface of the tape, bending the lamellae slightly in one direction and then again in the other direction.
    b. After cleaning, do not touch the adhesive or slide surface with your fingers.

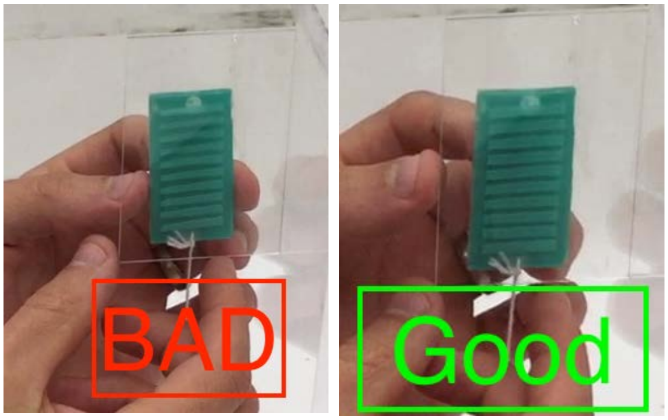

2. Load the adhesive
   a. Keeping the lamellae horizontal, gently place the adhesive strip onto the back side of the slide.
   b. As you place it on gently pull downward while making sure the lamellae all bend in the same direction and are uniformly bent (not sideways, or buckled).
   c. Holding the string, press on the back of the adhesive and pull up and down on the string (this is preloading).
   d. Select the desired weight and place weights onto the hook.

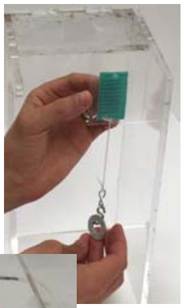

3. Measure the detachment angle
   a. Tilt the bottom of the slide toward you by placing your finger on the back left corner of the slide.
   b. Slowly tilt the slide at around 2.5° per second.
   c. Once the adhesive fails, record the last increment of 2.5° you passed. The angle you want to report is the angle between vertical and the last angle you passed (e.g. if it fell off right away, you would report an angle near 0°, if it didn't fall off until the slide was nearly horizontal, you would report an angle near 90°).
   d. Enter the total weight (adhesive + string + hook + weights) in grams and angle reached in degrees in the Google Form here: https://tinyurl.com/geckodata2020

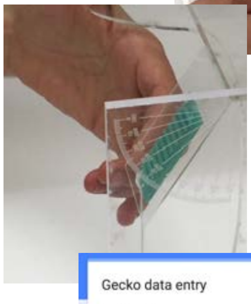

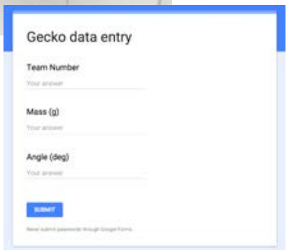

To analyze your gecko-inspired adhesive, you will:
1. Go to the spreadsheet: https://tinyurl.com/geckodatashareresponse2020
2. Locate your section's tab and look at the data columns and graph (called a "Limit Curve") which shows normal adhesive force versus shear force.
3. Verify shear force calculations ($F_{\parallel} = mg \cdot \cos \alpha$).
4. Verify adhesive force calculations ($F_{\perp} = mg \cdot \sin \alpha$).
5. What do you conclude about the nature of the Gecko-inspired Synthetic Adhesive? What features of the graph support the hypothesis that it is a "frictional adhesive"? What is the adhesive's maximum adhesive performance in Newtons? In atmospheres (i.e. force/area)? You'll need to measure the area of the adhesive, and use the fact that 1 $N/cm^2 \approx 0.1$ atm.

REPORT: PART A
As part of your team's final design report, document your testing protocols, results and conclusions. These will be combined with your design exercise in the next period.

SECOND PERIOD
OBJECTIVE – DEVELOP A TRIAL DESIGN USING GECKO INSPIRED ADHESIVE

In this design session you will:
1. Use your gecko-inspired adhesive as a design tool to propose a new product.
2. Describe a novel design that uses the gecko-inspired adhesive.
3. Attempt to communicate your best design in a mock-up of your choice. You should NOT expect it to actually work! This is a trial design, not even a prototype.

MATERIALS
For developing your gecko-inspired design, you will use:
1. Gecko-inspired adhesives made in the previous session.
2. Other patches of gecko-inspired adhesives manufactured by different procedures.
3. Select Choice A Construction Mock-up, Choice B Drawing/CAD/Simulation, Choice C Maker space or use or any combination.

APPROACH
To document your gecko-inspired design, you should:
1. Use low cost materials to make a mock-up for your trial design. Show by using a model or scale model to represent the structure, function and use of your trial design.
2. Create drawings/blue-prints or take photos of your mock-up to assist in explaining your trial design.
3. Consider all the design issues raised in our Analogy Check table. Use the table as a guide.

**Behaviors**<br>(What do you want system to do?)
**Structural Components**<br>(What can the structure be?)
**Operating Environment**<br>(Where?)
**Size**<br>(What size needed?)
**Functional Mechanisms**<br>(How do you want the system to work?)
**Characteristics/Specification**<br>(What are your specifications?)

**Performance Criteria**<br>(How well must the system work?)
**Constraints**<br>(Can compromises be removed?)

REPORT: PART B

Your report should include:

1. A one page description of your trial design that includes a consideration of system behavior, structure, environment, size, functional mechanism, specifications, performance, and constraints.
2. Diagrams/blue-prints, or screen shots from computer and/or photographs of your trial design.

FINAL REPORT

Your team will submit a single, final report (pdf) that includes:

1. A short description (200 word abstract) of your trial design.
2. PART A. One-page characterization of your team's testing protocols, results (2 Tables with your data and a plot which can include class data) and conclusions from your gecko-inspired synthetic adhesive which you manufactured and analyzed in the first session (Feb. 28).
3. PART B. One page description of your trial design that includes a consideration of system behavior, structure, environment, size, functional mechanism, specifications, performance, and constraints (as they relate to the principle involved in the original discovery!).
4. Representation of your trial design. Your team should include diagrams/blue-prints, or screen shots from computer and/or photographs of your trial design.
5. Trial design evaluation. One page description of the next steps if you were to follow up on your design. Who would you collaborate with to do more research and testing? What critical pieces of information would you need? What are the likely major roadblocks?

SELECTED READINGS

1. Autumn, K., & Gravish, N. (2008). Gecko adhesion: evolutionary nanotechnology. *Philosophical Transactions of the Royal Society of London A: Mathematical, Physical and Engineering Sciences*, *366*(1870), 1575-1590.
2. Autumn, K., Dittmore, A., Santos, D., Spenko, M. and Cutkosky, M. (2006a). Frictional adhesion: a new angle on gecko attachment. Journal of Experimental Biology 209, 3569-3579.
3. Autumn, K., Majidi, C., Groff, R., Dittmore, A. and Fearing, R. (2006b). Effective elastic modulus of isolated gecko setal arrays. J. Exp. Biol. 209, 3558-3568.
4. Autumn, K., Liang, Y. A., Hsieh, S. T., Zesch, W., Chan, W.-P., Kenny, W. T., Fearing,

R. and Full, R. J. (2000). Adhesive force of a single gecko foot-hair. Nature 405, 681-685.

5. Autumn, Kellar, Metin Sitti, Yiching A. Liang, Anne M. Peattie, Wendy R. Hansen, Simon Sponberg, Thomas W. Kenny, Ronald Fearing, Jacob N. Israelachvili, and Robert J. Full. "Evidence for van der Waals adhesion in gecko setae." *Proceedings of the National Academy of Sciences* 99, no. 19 (2002): 12252-12256.

SAFETY FIRST

1. Please be careful with this process!
2. Use safety glasses when mixing the PDMS.
3. We will be using razor blades to trim the PDMS adhesive. Proper use of the blade is essential - do not cut towards your hand or fingers! If you are unsure of a safe way to use the blade, ask a GSI. Place the blade safely away when finished.
4. The edges of the glass slides may be sharp, and if dropped, the slide may break into very sharp pieces. Please handle with caution.
5. We will also be cleaning the glass slides with isopropyl alcohol. Keep the isopropyl away from your eyes and face. Do not squirt the isopropyl on anything except a wadded paper towel.

Supplement S4 - Bioinspired Robot Design Project

Designing a Robotic Device

BACKGROUND

In this bioinspired robot design project, you will create a novel trial design based on a hexapedal robot that you will build in the first period. The robot was inspired from many of the principles that we discussed in lectures on biomotion of walking and running, control of locomotion, biosensing, and biopower or actuation. In the first design session, you will build the biologically inspired robot named DASH (**D**ynamic **A**utonomous **S**prawled **H**exapod). DASH Robotics Inc. (Kamigami Robots) is a start-up robotics company founded by Cal PhD students. To see more about these robots go to their YouTube Channel: https://www.youtube.com/channel/UCZyW7P5MlPd3iLgS4UjazCQ and http://kamigamirobots.com.

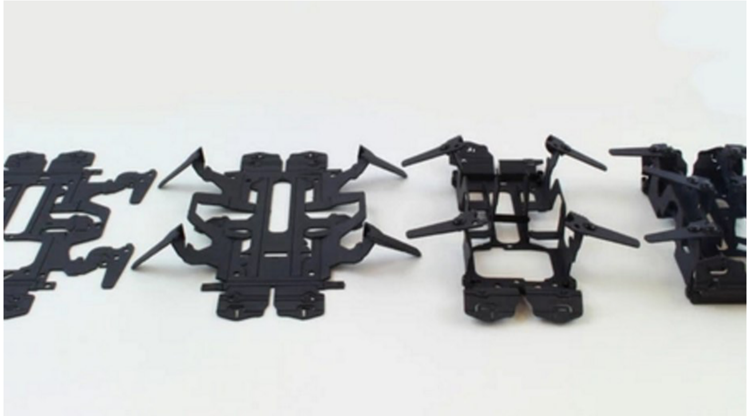

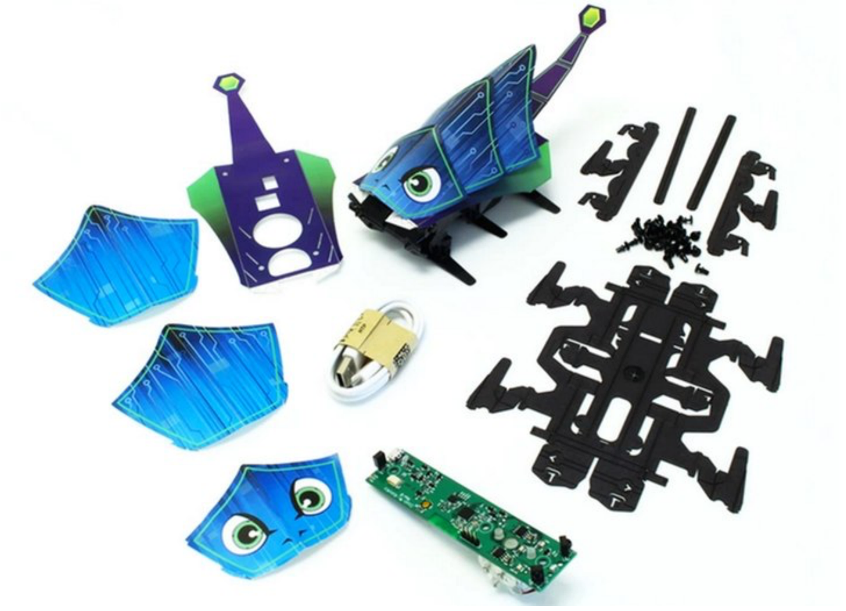

In the first design session, you will begin to characterize the biologically inspired robot with respect to its speed, and its ability to cross gaps. In the second design session you will finish characterizing the robot performance and also will create a novel trial design based on the DASH robot's capabilities. You will consider how you might modify the robot to serve a function that will have societal impact. In addition to modifications of the DASH robot that you have built, your design can include a biologically inspired sensor to collect data about the environment. You will search for one research paper that describes a biological sensor that has not yet been translated into a device. To express your trial design idea, you will select Choice A Construction Mock-up, Choice B Drawing/CAD/Simulation, Choice C Maker space use, or any combination. Essentially, you will be using a Compound Analogy that includes both the bioinspired robot and bioinspired sensor. We plan to allow this option in your Final Project.

FIRST PERIOD
OBJECTIVE – BUILD A LEGGED RUNNING ROBOT
In this design session you will:
1. Construct a legged running robot
2. Begin to characterize the robot's performance
3. Write a description on how the robot is or is not biologically inspired and how these features give the robot capabilities that other robots do not possess.

MATERIALS
For building your running robot, you will use:
1. a DASH kit
2. iOS or Android App and Controller. Download Kamigami 2016 (Black logo) from App Store.

METHODS
Go to Files/Discussion Section Assignments SP 2019/ 8_Legged robot project_18/DASH_Video_Instructions and/or follow the attached handout. You may also follow instructions on http://instructions.dashrobotics.com.
In addition, you could also find instructions by:
1. Finding the power button on the underside of your motor board.
2. Push the button so the lights on the top of the motor turn on.
3. Go to the Kamigami app on your phone and press the "Connect and Play" button.
4. The app will find your motor and say it has detected a new robot.
5. Follow the prompts and name your robot.
6. After naming your robot, the app will give instructions for how to build your robot.

ANALYSIS
To characterize your robot's performance, you will:
1. Estimate its maximum speed in m/sec. Time your robot over a known distance.
2. Test performance on crossing gaps. Use two lab benches to create a gap. Place a piece of tape at a known distance in front of the gap. Adjust the maximum speed to

a chosen value in the Kamigami Robots phone app, and then run the robot at full throttle towards the gap. Make sure to start the robot far enough away that it reaches full speed by the time it gets to the tape. Measure the speed, and the maximum gap size the robot can cross at that speed.

Add your data to the prepared Google Spreadsheet (https://tinyurl.com/robotsdata2019), so you can share with the class.

REPORT: PART A
As part of your team's final design report, document your testing protocols, results and conclusions. Include a description on how the robot is or is not biologically inspired and how these features give the robot capabilities that other robots do not possess. These will be combined with your design exercise in the next period.

SECOND PERIOD
OBJECTIVE – DEVELOP A TRIAL DESIGN USING THE LEGGED ROBOT

In this design session you will:

1. Finish characterizing your robot's performance.
2. Use your robot as a design tool to propose a new product.
3. Describe a novel design that uses the running robot. Consider how modifications and additions could result in novel behaviors and capabilities. Add a biological sensor of your choice. **Include one publication of a biological sensing capability that could be designed.**
4. Attempt to communicate your best design by modifying the present robot. You should NOT expect it to actually work! This is a trial design, not even a prototype.

MATERIALS
For developing your robot design, you will use:

1. Running robot made in the previous session.
2. Any other inexpensive materials (cardboard, fabric, plastic, wood) that assist you in expressing your design idea. Select Choice A Construction Mock-up, Choice B Drawing/CAD/Simulation, Choice C Maker space use, or any combination.

APPROACH
To develop your robot design, you should:

1. Use low cost materials to make a mock-up for your trial design. You may add or modify the robot to show a novel capability.
2. Create a drawing/blue-print and take a photo of your mock-up to assist in explaining your trial design.
3. Consider all the design issues raised in our Analogy Check table. Use the table as a guide.

**Behaviors** (What do you want system to do?)
**Structural Components** (What can the structure be?)
**Operating Environment** (Where?)
**Size** (What size needed?)
**Functional Mechanisms** (How do you want the system to work?)
**Characteristics/Specification** (What are your specifications?)
**Performance Criteria** (How well must the system work?)
**Constraints** (Can compromises be removed?)

REPORT: PART B

Your report should include:

1. A one page description (single spaced) of your trial design that includes a consideration of system behavior, structure, environment, size, functional mechanism, specifications, performance, and constraints.
2. One diagram/blue-print or one photograph of your trial design.
3. A research publication of a biologically inspired sensor.

FINAL REPORT

Your team will submit a single, final report that includes:

1. A short description (200 word abstract) of your trial design.
2. PART A. One page (single spaced) characterization of your team’s testing protocols, results and conclusions from your running robot that you built and analyzed in the first session. Include a description on how the robot is or is not biologically inspired and how these features give the robot capabilities that other robots do not possess.
4. PART B. One page (single spaced) description of your trial design that includes a consideration of system behavior, structure, environment, size, functional mechanism, specifications, performance, and constraints (as they relate to the principle involved in the original discovery!). One diagram/blue-print and one photograph of your trial design. Include a pdf of the biologically inspired sensor you selected.

5. Trial design evaluation. One page (single spaced) description of the next steps if you were to follow up on your design. Who would you collaborate with? What critical pieces of information would you need? What are the likely major roadblocks?

SELECTED READINGS

1. Birkmeyer, Paul, Kevin Peterson, and Ronald S. Fearing. "DASH: A dynamic 16g hexapedal robot." In *Intelligent Robots and Systems, 2009. IROS 2009. IEEE/RSJ International Conference on*, pp. 2683-2689. IEEE, 2009.
2. Birkmeyer, Paul. *The Design and Performance of DASH*. 2010.
3. Birkmeyer, Paul, Andrew G. Gillies, and Ronald S. Fearing. "CLASH: Climbing vertical loose cloth." *Intelligent Robots and Systems (IROS), 2011 IEEE/RSJ International Conference on*. IEEE, 2011.
4. Birkmeyer, Paul, Andrew G. Gillies, and Ronald S. Fearing. "Dynamic climbing of near-vertical smooth surfaces." *Intelligent Robots and Systems (IROS), 2012 IEEE/RSJ International Conference on*. IEEE, 2012.
5. Full, R.J. and Tu, M.S. 1990. The mechanics of six-legged runners. J. exp. Biol. **148**, 129-146.
6. Full, R.J. and Tu, M.S. 1991. Mechanics of rapid running insects: two-, four-, and six-legged locomotion. J. exp Bio. **156**, 215-231.
7. Full, R.J. and Blickhan, R. and Ting, L.H. 1991. Leg design in hexapedal runners. J. exp Bio. **158**, 369-390.
8. Blickhan, R. and Full, R.J. 1993. Similarity in multilegged locomotion: Bouncing like a monopode. J. comp. Physiol. **173**, 509-517.
9. Jindrich, D.L. and Full, R.J. 1999. Many-legged maneuverability: dynamics of turning in hexapods. J. exp Bio. **202**, 1603-1623.
10. Jindrich, D.L. and Full, R.J. 2002. Dynamic stabilization of rapid hexapedal locomotion. J. exp Bio. **205**, 2803-2823.
11. Goldman, D.I., Chen, T.S., Dudek, D.M. and Full, R.J. 2006. Dynamics of rapid vertical climbing in cockroaches reveals a template. J. exp Bio. 209, 2990-3000.
12. Lee, J., S. Sponberg, O. Loh, A. Lamperski, R.J. Full, and N. Cowan. 2008. Templates and anchors for antenna-based wall following in cockroaches and robots. IEEE Transactions on Robotics. 24, 130-143.
13. Qian, F., Zhang, Korff, W., Umbanhowar, P., Full, R. J. and D. Goldman. 2015. Principles of foot design in robots and animals determining terradynamic performance on flowable ground. *Bioinspiration & Biomimetics*. 10 (4), 046003.
