# Supplementary material for: Eyes Toward Tomorrow Program Enhancing Collaboration, Connections, and Community Using Bioinspired Design
Source: Integr Comp Biol. 2021 Aug 30;61(5):1966–80. doi: 10.1093/icb/icab187 (PMC8699102; doi:10.1093/icb/icab187)
Supplement: icab187_Supplemental_Files [file icab187_supplemental_files.zip › icb-2021-0200-File008.pdf]

Supplement S5 – Team Final Video and Poster Project

Biologically Inspired Design - IB 32, L&S 30
Team Final Video and Poster Project

Final Project (30% of Final Grade – 180 points)

Your "final exam" will be a 5 min video and poster of a bioinspired design of your team's choice. The video should be posted by **6PM on May 1st**.
The poster should be submitted by **6pm on May 1st**.

All final projects will be presented at the **Jacobs Spring Design Showcase on May 8th** (310 Jacobs 1:45PM - 3:45PM) (https://www.eventbrite.com/e/jacobs-spring-design-showcase-2019-tickets-59346549126?aff=ebdssbdestsearch). Space and facilities will be available for videos, actual trial designs and posters. Open to the public, investors and companies.
Space available for your videos and posters will be supplied by Adobe Behance – see https://www.behance.net/berkeleybiodesign for example.

Instructions for Assignment

You should name your invention (Conceptual Design) and you may also name your Team or start-up "Company."

We encourage your Team to start now. The most common response from Team's with final design projects in other courses is that they wish they started earlier.

1. Team Box Folder. Access Box in the left navigation bar in bCourses. We will be available to advise you at all stages of your final project. We encourage your Team to use your Box Folder so we can easily view your possible research publication selection (we have made a Folder for this for each Team), your Discovery Decomposition, Analogy Check, Trial Design and your final video production.

2. Preliminary Research Publication Selection & Trial Design Ideas (Due April 11th, 6PM). Your Team should select 3 research publications by April 11th. Submit three possibilities for a Trial design. Place these in your Box Folder.

3. Collaborative Plan v1 (Due April 11th, 6PM). Your Team's Collaborative Plan is due April 11th. Collaborative_Plan_Teamplate.docx

Please watch the teaming videos for assistance. Teaming Video_Introduction_1.mov, Collaborative_Plan_2.mov, Excel_by_Teaming_3.mov.

Have a Team meeting, discuss your Goals (small g and BIG G), Roles, Procedures, and Relationships, using the prompts in the Table. Document your discussion and agreements by filling in the Collaborative Plan Template, removing each prompt as you respond to it, or using the blank template using the one below as a guide. The template expands: use as many pages as you need. Place the Plan in your Box Folder. Name file: CollabPlanTeam#.

**Team Name:** ______________________________ **Date:** ______________	
**Goals**	• What are the **personal goals (small g) of each member on this team**? (Team Member #1,#2, #3, etc: 1 or more goals/ name)<br>• What is the **Project GOAL (big G)** we're all committed to achieve together?<br>• Is our **Project Goal scaled** to our resources (dreams, materials, skills, differences, etc.) and constraints (assignment, time, skills, etc.)<br>• What are the **metrics for success** for what we're producing?
**Roles**	• **Who** is responsible for **which deliverables?**<br>• **Which deliverables** require **collaboration, subgroups & individual work? Who** does each person **depend upon to succeed?**<br>• Do we need a **project manager** to coordinate?<br>• What are the **deliverables each person** is accountable to produce?
**Procedures**	• **Decision Making -** What **process** shall we use: consensus, majority rules, deference to expert, default to the loudest, or?<br>• **Effective Meetings -** Focus on key, timely decisions together vs. status/update (offline);<br>• **Meeting roles:** scribe, facilitator, time keeper<br>• **Communication - FTF:** frequency, time, location**; type of technology:** (Box, Googledocs, Hangout, etc.); **expectations for responsiveness; 'best time to work'** (AM, PM, weekends?)
**Relationships**	• **Team Diversity –** What is the diversity on our team? Disciplines to tap for solutions; individual learning styles for the stages of invention; overall team learning style strengths and places to supplement; cultural backgrounds, work experience, dreams to leverage for scope & impact of goals, new roles, better procedures; languages for more diverse customer set, bigger market;<br>• **Listening –** Notice binary thinking, auto-rankism, and go beyond it.<br>• **Team Name**–What's a **team name** that captures who we are and what we're going to do?

Team Name: ____ Date: __________________	
**Goals**	
**Roles**	
**Procedures**	
**Relationships**	

Design Project Team Contributions. Please let us know if you are having difficulty accessing publications. Please assume that if a teammate isn't as responsive as you hoped that they may be experiencing additional hardships.
Only develop your collaborative plan as much as possible reflecting the particular challenges of all team members. All we ask is that each of you do what you can given your circumstances, no more. Do not worry. We will be understanding of each of your situations. We need to reduce stress, not create more. Please feel free to openly communicate with any of us.

4. Final Research Publication Selection & Trial Design Idea (Due April 18th, 6PM). Your Team should select your final research publication for Decomposition, Analogy Check and translation of the biological principle by April 18th. Submit your final Trial design idea. Place these in your Box Folder. Name FinalPub_Team# and FinalTrialDesignIdeaTeam# Remember you should contact Professor Full or the GSIs if you need advice at any time during your project development.

5. Revised Collaborative Plan v2 (Due April 18th, 6PM). Your Team should submit a revised Collaborative Plan v2 by April 18th. Place these in your Box Folder. Name FinalCollabPLab_Team#.

6. Jacobs Facilities and your Trial Design. Although your Team may again select Choice A Construction Mock-up, Choice B Drawing/CAD/Simulation, Choice C Maker space use, or any combination, we strongly encourage you to sign-up for training in Jacobs (especially laser cutting

and 3D Printing) if you have not done so. You have an incredible opportunity to use these facilities.

Final Video

Your Final Video should include:

1. General statement of the biological discovery.

Select a research paper containing a benchmark biological discovery (**NOT** a review paper or popular article).

Do **NOT** select a study that has already been used to produce a bioinspired design unless your idea is completely different.

Upload possible paper selections into your Team's Box Folder.

Get the research paper approved by Professor Full and the GSIs.

If your bioinspired design requires two bioinspired principles (Compound Analogy), then select two research publications. Remember, if you select two publications, your Team must Decompose both publications.

Candidate journals (many others are acceptable)

Journal of Experimental Biology (http://jeb.biologists.org/)
Journal of the Royal Society London Proceedings B (http://rspb.royalsocietypublishing.org/)
Journal of the Royal Society London Interface (http://rsif.royalsocietypublishing.org/)
Nature (http://www.nature.com/)
Science (http://www.sciencemag.org/)
Proceeding of the National Academy of Sciences (http://www.pnas.org/)

2. Discovery decomposition (explain as if researcher at conference; need not show diagram). Include a breakdown of your research publication using the Discovery Decomposition approach discussed in class and lecture and completed by you in several individual assignments. Your final video project should include the decomposition, but you can show it using a presentation program (Powerpoint/Keynote that you record as a video) that includes figures from the paper, diagrams, photos or relevant videos that best express the discovery of a principle your Team will use as inspiration. **You should not show the decomposition in the Final video, but the information in the decomposition needs to be presented.**

EXAMPLE:

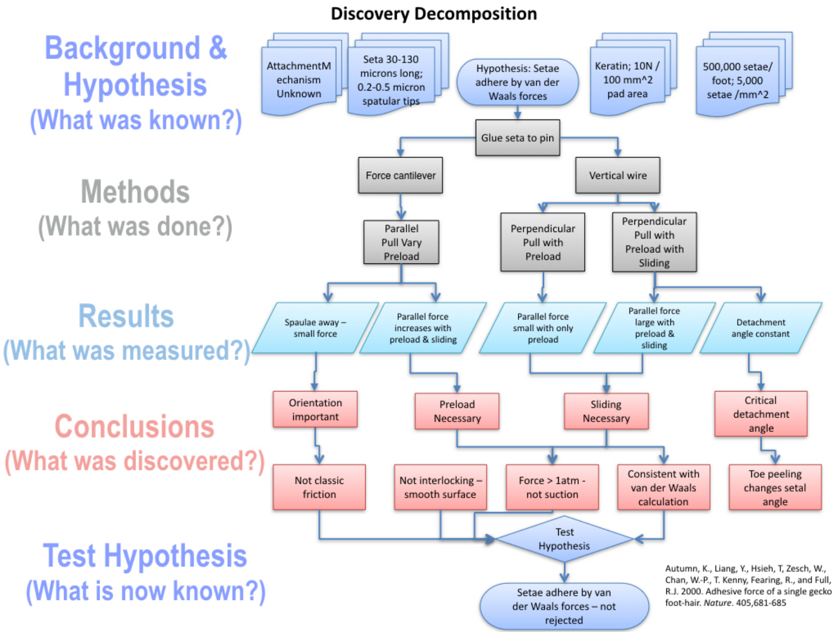

3. Analogy Check. Create an Analogy Check Table using the approach discussed in class and lecture. Show your Analogical Check Table to clearly state the strengths AND weakness of your analogy. Produce your table using the structure below as you did for previous assignments and suggest a novel design and **invention name**. In the first column, fill in as much as possible from what you have learned from the publication Discovery Decomposition. In the second column, Design Problem, make sure you fill out your best guess about your new design. In the middle column indicate whether the analogy Weak (need more experimentation), Valid (translation possible) or Uncertain (need more information) or N/A (not applicable). Remember the invention side MUST discuss the analogy/translation and NOT just any aspect of your design. (For example, if your address size, it should relate to the scaling the bioinspiration, not simply the size of your whole invention.) You may express your Analogy Check Table by using a presentation program (Powerpoint/Keynote that you record as a video) that includes diagrams, photos or relevant videos. **You should not show the Table in the Final video, but the information in the Table needs to be presented.**

Design Solution	Analogy Check	Design Problem
**Bioinspiration**		**Invention Name**
Function<br>(What does system or organism do?)		Function<br>(What do you want system to do?)
**Structural Components**<br>(What is structure or organization of system?)		**Structural Components**<br>(What can the structure be?)
**Operating Environment**<br>(Where?)		**Operating Environment**<br>(Where?)
**Size**<br>(What is size?)		**Size**<br>(What size needed?)
**Mechanisms**<br>(How does system work?)		**Mechanisms**<br>(How do you want the system to work?)
**Characteristics/Specification**<br>(Which are distinguishing?)		**Characteristics/Specification**<br>(What are your specifications?)
**Performance Criteria**<br>(How well does system work?)		**Performance Criteria**<br>(How well must the system work?)
**Constraints**<br>(What compromises system?)		**Constraints**<br>(Can compromises be removed?)

4. Proposed bioinspired trial design. State your proposed bioinspired design. Show your trial design using photographs, diagram/blue-print, CAD drawings, simulations and/or videos. Here your Team should explain the details.

5. Trial design evaluation. Describe the next steps if you were to follow up on your design. Who would you collaborate with? What critical pieces of information would you need? What are the likely major roadblocks? Reference back to your Analogy Check Table.

6. Product Pitch. Sell your trial design as if you were presenting to venture capitalists, an established company or beginning your own start-up.
Include:
The intended use;
The novelty of the design;
Possible societal impact (health, fitness, environment, safety, security, education, connections to others or community, assisting underserved, disabled populations or underdeveloped countries, sports and entertainment);
Your mock-up, prototype, computer simulation/animation in combination with the setting in which your design is to be used. Be creative.

Do NOT make a late-night, humorous commercial selling your design for $19.95!
We will provide several examples from last year to guide you.

7. List the references and resources you used and acknowledge sources of assistance at the end.

Final Poster

Your team will create a poster to display at the Jacobs On-line Showcase. You want the people who see it to understand the nature of your design and the inspiration behind it and to be able to ask informed questions about your proposals. Your poster should include:

1. The name of your design
2. Your team name and Team #
3. A section about the issue that motivated you to create your design. In this section, you might mention societal issues or shortcomings in current technologies or any other issue that your design could address.
4. A section about the biological inspiration behind your design. In this section, you should briefly describe the major findings from the BioDiscovery article that inspired your design. You should explain what your organism does that is extraordinary or exemplary and how it does this.
5. A section about the design itself. This section should relate back to the previous two sections. It should be clear how your design is inspired by the BioDiscovery you selected and how it addresses the issue you raised. This section should also include a visual representation of your design.
6. A section about the next steps you need to take. You might mention testing that needs to be done, areas for improvement, and potential collaborators.

You have the freedom to organize the poster however you would like, as long as you cover the information outlined above. **Try to minimize words when possible and use figures to communicate your ideas.** The poster should be engaging but not overwhelming.

7. Poster examples can be found in the bCourses folder Final Project_21.
8. Poster PDF files are due May 1st at 6PM. Please use the file name: Poster_Team# where # is your team number.

Due Dates

Assignment	Due Date
Preliminary Research Publications and trial design	April 8th, 6pm
Collaborative Plan	April 8th, 6pm
Final research Publication and trial design ideas	April 15th, 6pm
Revised Collaborative Plan	April 15th, 6pm
Final Poster	May 1st, 6pm
Final Video	May 1st, 6pm
