# Supplementary material for: Eyes Toward Tomorrow Program Enhancing Collaboration, Connections, and Community Using Bioinspired Design
Source: Integr Comp Biol. 2021 Aug 30;61(5):1966–80. doi: 10.1093/icb/icab187 (PMC8699102; doi:10.1093/icb/icab187)
Supplement: icab187_Supplemental_Files [file icab187_supplemental_files.zip › icb-2021-0200-File009.pdf]

Supplement S6 - Teaming Survey 2021

The respondent's email (**null**) was recorded on submission of this form.

* Required

Email *

Discussion Section *

Mark only one oval.

- 101
- 102
- 103
- 104
- 105
- 106

Last Name *

First Name *

1. Level *

Mark only one oval.

- Freshman
- Sophomore
- Junior
- Senior
- Graduate student

2. Intended Major *

Mark only one oval.

- INTEGRATIVE BIOLOGY
- MO&CEL BIO
- MOL ENV BIOLOGY
- PSYCHOLOGY
- COGNITIVE SCIENCE
- ARCHITECTURE
- ECONOMICS/BUSINESS
- DATA SCIENCE
- BIOENGINEERING
- MECHANICAL ENGR
- ELEC ENGR COMPUT SCI
- ENGR-MAT SCI & ENGR
- Other: ____________________

3. Have you taken a class in basic biology that includes a survey of diverse animal taxa? *

Mark only one oval.

- ( ) Yes
- ( ) No

If you answered "yes", please list relevant classes

4. Have you taken a class in anatomy, physiology or biomechanics? *

Mark only one oval.

- ( ) Yes
- ( ) No

If you answered "yes", please list relevant classes

5. Do you have experience using “Maker Space” equipment such as laser cutters, 3D printers, CNC routers and mills? *

Mark only one oval.

- Yes
- No

6. Do you have experience with basic electronics, building circuits, soldering, or embedded programing (i.e. Arduino)? *

Mark only one oval.

- Yes
- No

7. Do you have experience with woodworking, sewing, sculpting or other basic fabrication skills? *

Mark only one oval.

- Yes
- No

8. Are you familiar with 2D or 3D CAD tools, such as Solidworks, AutoCAD, or Adobe Illustrator? *

Mark only one oval.

- Yes
- No

9. Are you familiar with 2D or 3D artistic or modeling tools, such as Photoshop, SketchUp, Maya, or 3DS Max? *

Mark only one oval.

- Yes
- No

10. Do you have experience working in a small team on a goal-driven project? *

Mark only one oval.

- Yes
- No

11. Have you taken a class that has a design component or project? *

Mark only one oval.

- Yes
- No

If you answered "yes", please list relevant classes

12. Are you living on-campus or off-campus this semester? *

Mark only one oval.

- On-campus
- Off-campus *Skip to question 20*

Mailing Address

Please enter your complete mailing address so that we can ship course materials to your residence.

This content is neither created nor endorsed by Google.

Google Forms

Supplement S7 – Teaming Activity

Seed Dispersal Activity

This activity was adapted from Tom Wujec's "Marshmallow Challenge" and the "Gone with the Wind" activity from Scientific American. For more info, see:

- TED Talk: Build a tower, build a team | Tom Wujec
- Marshmallow Challenge Guide (PDF)
- Gone with the Wind: Plant Seed Dispersal

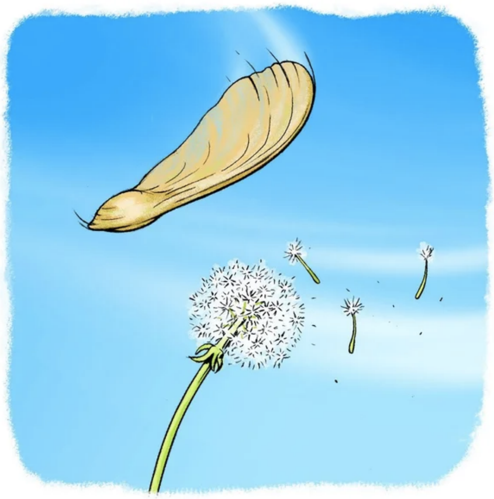

Photo Credit: George Retseck

Background – Seed Dispersal

Dispersal of seeds is crucial for the survival of plant species.

- If plants grow too closely together, they end up competing for the same light, water and nutrients.
- Seed dispersal allows plants to increase their range while minimizing competition for the same resources.
- The better a seed can be dispersed into the air, the farther it can be blown by the wind, helping the plant species widely distribute potential offspring.
- Plants that rely on seed dispersal need to **maximize** their ability to disperse seeds relative to the **constraints** of their anatomy and size…
- Greater height of the seedhead increases the probability of dispersal, but also increases the probability of stem collapse - an example of the trade-off's organisms face.

<u>Activity</u>

In front of you are the anatomical components of a "plant" that uses wind for seed dispersal.

- 20 pieces of spaghetti (dry)
- 1 meter of masking tape
- 1 meter piece of string
- 1 large marshmallow
- Scissors (to adjust tape and string size as needed)

Your team must maximize seed dispersal by building the tallest plant possible. Importantly, the seeds of your plant disperse from the *seedhead*—the marshmallow.

Rules:

1. Build the TALLEST freestanding plant measured from the tabletop surface to the top of the seedhead (marshmallow). The plant cannot be suspended from a higher structure, like a chair, ceiling or chandelier.
2. The ENTIRE seedhead must be on TOP of the plant.
3. Cutting or eating part of the seedhead disqualifies the team.
4. You can use as many or as few of the stem structures (spaghetti sticks) and as much or as little of the string/tape as you prefer. You CANNOT use the paper bag as part of your structure.
5. You are free to break the spaghetti or cut up the string/tape to create novel structures.

The challenge lasts **18 minutes**. You CANNOT hold on to the structure when time runs out. Those touching or supporting the structure at the end of the activity are disqualified.

Debrief – Discussion Questions

Instructions: Turn and talk with your group, then we'll discuss as a class.

1. Which strategies worked well? Which strategies did not? Why?
2. How did different roles emerge during the activity? How was the workload distributed among everyone?
3. What will you take away from this activity? How will this help you be a better member of your team?
4. How can you keep all members of the team even more fully engaged?
5. What were some design constraints that you had to deal with?

Norm Cards

From the Edible Schoolyards Project

Putting Ideas on the Table
Ideas are the heart of group work. In order to be effective, they must be released to the group. "Here is an idea for consideration," or "I am putting this idea on the table." It is equally important to know when to remove an idea from the table. Use signal words such as "I think this idea is blocking our thinking and I want to remove it from the table." When ideas are "owned" by individuals, other group members' responses tend to reflect their feelings for and their relationship to the speaker, and may not be specific to the ideas presented.

Paying Attention to Self and Others
Meaningful dialogue and discussion is facilitated when each group member is conscious of oneself and others. This consciousness includes being aware of your own and others posture, gesture, and other non-verbals. Paying attention to self and others could include the amount of talking, the amount of silence, or responding to others' information delivery or language style.

Presuming Positive Intent
Assuming that others' intentions are positive encourages honest conversations about important matters. Positive presuppositions reduce the possibility of the listener perceiving threats and challenges in a paraphrase or question. Group members can signal this by saying: "Presuming positive intent, I'm thinking that…"

Probing for Specificity
Human brains are not always designed for specificity. We often form quick generalizations from fragments of information. These quick judgments based on assumptions can cause difficulties in communication. Five areas contributing to overuse of generalizations are vague nouns and pronouns, vague action words and comparators, rule words and universal quantifiers. Probing action asks members to remove the generalization and cite the exact data.

Pursuing a Balance Between Advocacy and Inquiry
Try to spend equal amounts of time and energy advocating for one's own ideas and inquiring into the ideas of others. Creating a balance of advocacy and inquiry requires both emotional and cognitive resources. This balance is most necessary at the exact point when many group members are least likely to want to inquire into the ideas of others. It is at the moment of greatest disagreement that this norm makes the biggest difference for productive communication.

Paraphrasing
Paraphrasing is one of the most valuable and least used communication tools in meetings. A paraphrase can be used effectively with a question. First paraphrase, and then ask a question. Practice this skill and notice what happens to the dynamics of the conversation. Paraphrasing aligns the parties and creates a safe environment for thinking. Levels of paraphrasing may include any of the following: clarify speaker statement; summarize what was said; or shifting what was said to include an overarching purpose.

Marshmallow Challenge Results

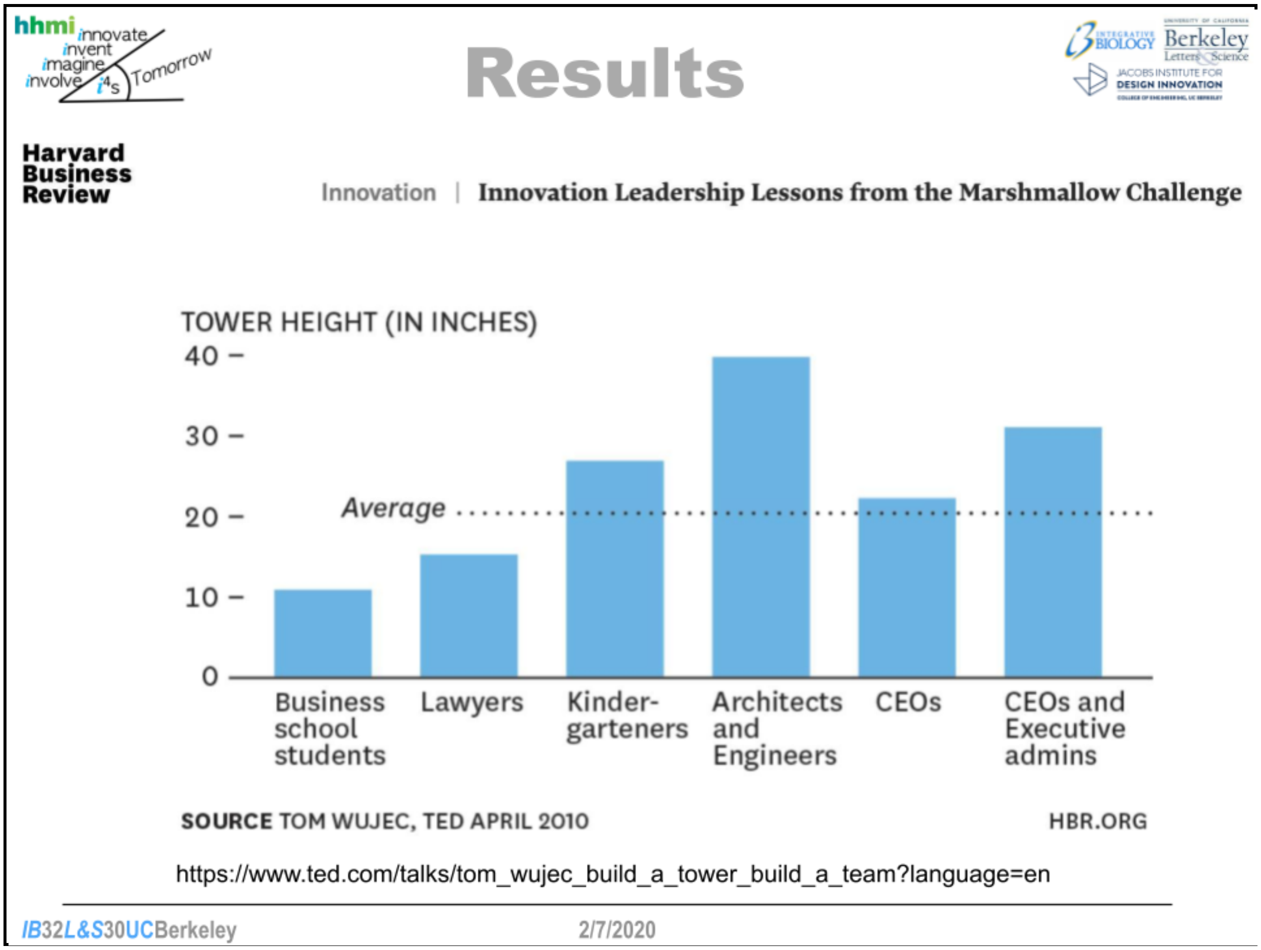

Discussion question: Why do kindergartners perform so well?

Supplement S8 - 3D Printing Makerspace Activities

PROSTHETIC HAND FOR CHILDREN PROJECT
3D Printing Makerspace Activity

PLEASE CHECK FOR HANDOUT UPDATES

To experience the future of design, you and your team will participate in an early activity using your Maker Pass. You will 3D print prepared files of a prosthetic hand for children. See Sophie's Super Hand 2015: https://engineering.berkeley.edu/magazine/fall-2015/sophies-super-hand

New BioDesign Exercise

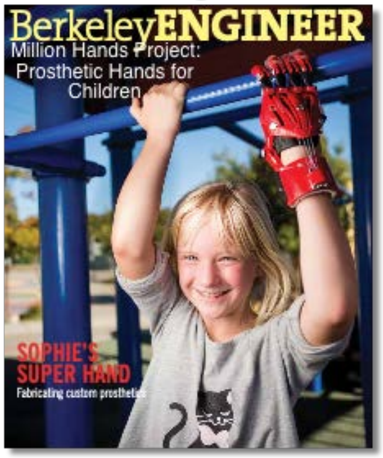

Symbrachydactyly
Finger bones never developed

PRE-ACTIVITY REQUIREMENTS
Please complete these **steps by THURSDAY JANUARY 30th**.
If they are not completed before discussion on Friday January 31st, you may not be able to participate during the in-class print.

1. **Purchase Jacobs Hall Maker Pass**
   - Purchase the Jacobs Hall Maker Pass HERE - The Maker Pass is $100 for the semester and is required for this course. Waivers are available to students with financial need, so if you are unable to pay the fee out of pocket please contact jacobsinstitute@berkeley.edu.
2. **Complete General Safety Training**
   - Regardless of if you have purchased a Maker Pass, enroll in the Maker Pass Equipment and Safety bCourses page.
   - On the Maker Pass Equipment and Safety home page read the General Safety training curriculum, take the online quiz, and complete the three General Safety Agreements (1, 2, 3).
3. **Download Cura 3.5**
   - Visit the Ultimaker 3D Printer Landing Page. This page has all the information you will need to get trained and download the correct software.
   - Under the 'Downloads' subsection you will see two download links for the Cura 3.5 software, one for Windows and one for Mac. Download the correct version for your personal laptop. If you do not have a personal laptop, skip this step and the next.
   - If you are using a Windows computer, it is recommended you download Bonjour for an easy network connection. This download link is also mentioned and available in the training module.
4. **Ultimaker 3D Printer Training Module and Quiz**
   - While staying in the training manual, continue to read through the training curriculum and take the online quiz.
   - The training will guide you through all the steps on how to get Cura 3.5 up and running for your 3D print.
   - Make sure to download the Project File for the Ultimaker printers in Step 2 of the training. This ensures your print is compatible with the Jacobs Ultimakers.
   - You may take the quiz as many times as needed to pass.
5. **Download print file for in-class activity** HERE
   - This is the wrist pin cap file, which is only being used as an in-class example and will not be required to print.

IN-CLASS HANDS ON TRAINING PROCEDURES (FRI JANUARY 31)
During class, we will have a live demo to teach you how to use 3D printer software and the Ultimaker 3D printers.

1. Sit in your team.
2. In class 3D printer software training
3. Jacobs 3D printing training/tour
4. Return to class

ASSIGNMENT (DUE THURSDAY FEBRUARY 13)
Now that you have the skills to 3D print, we want you to make something on your own.
Each student in your team will print a finger of the prosthetic hand by Thursday February 13th.
This is a total of 5 fingers/prints you will be responsible for. We will print the wrist and other components for you.

1. Download the eNABLE hand finger files HERE

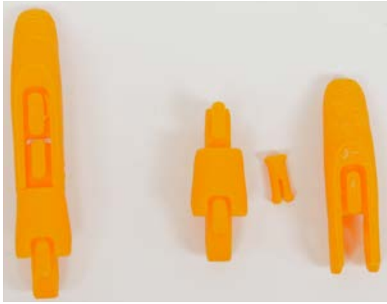

- For each hand there are different types of fingers: 2 short, 2 long, and 1 thumb.
- Each finger consists of 3 parts: the fingertip, the phalanx, and a pin that connects the two at a bendable joint.
- Determine who in your group is printing which finger type and download your respective files. Each team is responsible for printing all 5 fingers.

2. Understand your print settings HERE
- When 3D printing, there are lots of settings you can change to alter the overall strength and quality of your product. Most of the settings you will need for this print will be preset by the opening the Ultimaker Project File as described in the training manual. However, there will be a few changes that it is important you remember!
- Before printing, make sure that under the *Support* tab, **Generate Support is unchecked**. And under the *Build Plate Adhesion* tab, the **Build Plate Adhesion Type is changed from Brim to Skirt.**
- You can also watch how this is done in the linked video above.

3. Print your finger file
- If you need or want help with your print, GSIs will be in Jacobs for office hours to offer more direct assistance. The Jacobs Hall staff is also a great resource.
- Because the printers are used on a queue system, try to start your prints earlier rather than later in the two-week period. This will give you more wiggle room in case something goes wrong.
- There will also be 3D printers available for your use in CiBER, located in 1099 VLSB. These printers are not Ultimakers and will require supervision during use. Thus, they will only be available during certain times which you can find on the Calendar in bCourses.
- Scheduling Printing in CiBER:
    1. Go to the course calendar in bCourses
    2. On the right-hand side, click on "Find Appointment"
    3. Select correct course from drop down menu.
    4. Available appointments will now show up on the calendar.
    5. Click on a time and date that will work and select "Reserve."

**** REMEMBER, SAFETY FIRST**
The extruder and print plate of the 3D printer can get very hot – do not touch them during printing and be cautious if you must touch them at other times.

4. Take a selfie with your 3D printed finger and submit it to bCourses Assignment -

- Please submit a photo that includes you, your 3D printed finger, and the 3D printer **individually**.
- Each student will be responsible for printing one finger, but you can print with your teams. Just make sure you get the personal experience of 3D printing. Please don't have one person print all the fingers.

ASSEMBLY (FRIDAY FEBRUARY 14)
Your team is going to build your prosthetic hand!
Before coming to class…
1. Read the eNABLE hand assembly manual, watch the assembly video, or both!
2. Assemble your finger – remember that the pin is inserted so the rectangular head fits into the rectangular slot of the finger joint pin hole.

We want you to be able to complete your hand during the discussion, SO PLEASE COME TO CLASS PREPARED! Understanding the workflow and being able to identify the parts will allow you and your teammates to work efficiently. These resources are also available on bCourses.

OFFICE HOURS / TUTORING

- GSIs will try to hold their office hours in Jacobs to help people with printing their files.
- The Berkeley Biodesign Community, GSIs, and other lab/course staff members will hold extra tutoring hours in Jacobs and CiBER (1099 VLSB) to help students as well.

SELECTED READINGS

1. Sophie's Super Hand 2015: https://engineering.berkeley.edu/magazine/fall-2015/sophies-super-hand
2. Ultimate Beginner's Guide to 3D Printing: https://www.makeuseof.com/tag/beginners-guide-3d-printing/

Link URLs

- Maker Pass Purchasing:
  http://jacobsinstitute.berkeley.edu/our-space/makerpass/get-maker-pass/

- Jacobs Maker Space Safety bCourse:
  https://bcourses.berkeley.edu/courses/1353091

- Bonjour software:
  https://support.apple.com/downloads/bonjour_for_windows

- In-Class Print File:
  https://bcourses.berkeley.edu/courses/1487597/files/folder/Discussion%20Section%20Assignments_20/2Discussion_1_31_20/In-Class%20Print%20File

- Print Setup Tutorial:
  https://bcourses.berkeley.edu/courses/1487597/files/folder/Discussion%20Section%20Assignments_20/2Discussion_1_31_20?preview=76473874

- Assembly Manual:
  https://bcourses.berkeley.edu/courses/1487597/files/folder/Discussion%20Section%20Assignments_20/2Discussion_1_31_20?preview=76432740

- Assembly Video:
  https://www.youtube.com/watch?v=Z-iI2OOyd4A&t=2s

- eNABLE finger files:
  https://bcourses.berkeley.edu/courses/1487597/files/folder/Discussion%20Section%20Assignments_20/2Discussion_1_31_20/Finger%20Files

PROSTHETIC FINGER SPLINT PROJECT
PLEASE CHECK FOR HANDOUT UPDATES

To experience the future of design, you and your team will participate in an early activity. You will 3D print prepared files of a prosthetic thermoplastic finger splint.

"Skeletal system fractures and accompanying soft tissue injuries are the most common injury type for patients. Splint application are very important for the management of simple fractures, dislocations and soft tissue injuries in upper extremities not requiring surgical treatment (1,2)." From Kadioglu, Emine, et al. "Manufacturing and application of personal hand and finger splint with three dimensional printer technology following hand and finger trauma." Annals of Medical Research 26.8 (2019): 1474-7.

One major advantage of thermoplastic splints is their ability to be remolded and reshaped to accommodate changes to the injured site due to tissue swelling and inflammation. Therapies that call for intermittent periods of immobility and gentle exercises of the injury are ideal for thermoplastic splints because they can be removed for physical therapy. Patients can wear the devices in the shower, or they may remove them for bathing. A thermoplastic splint can also be used to stabilize an injury prior to surgery as well as to provide fast immobilization to areas of trauma in emergency rooms.

Another advantage is that they can be made from low-cost materials and can therefore be made accessible to a wide range of people. Online design communities provide platforms for designers and engineers to share and revise new models. We found the model you will be printing on Thingiverse, where it was made available for free.

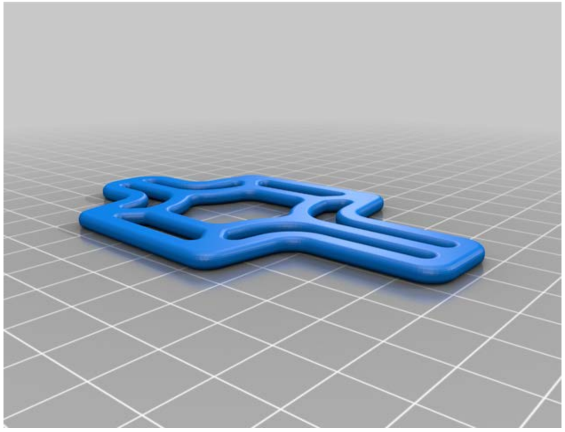

Created by FFFTechnology

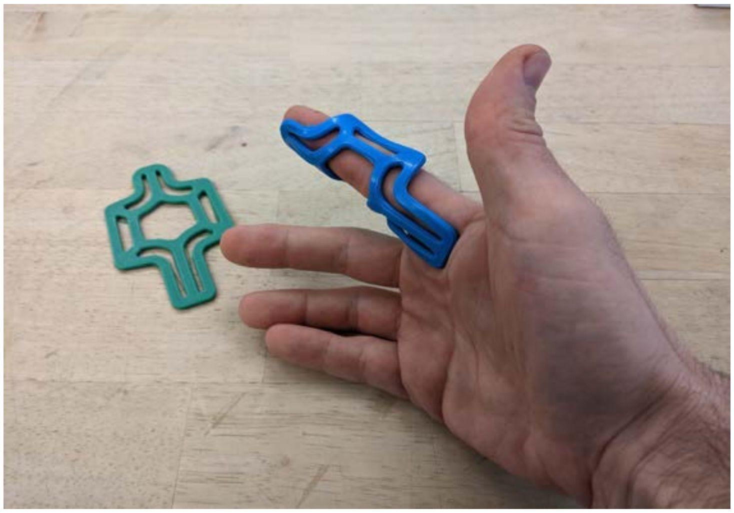

PRE-ACTIVITY REQUIREMENTS
Please complete these **steps by THURSDAY JANUARY 28th**.
If they are not completed before discussion on Friday January 29th, you may find it hard to follow along during the in-class training activity.

1. **Register for Jacobs Project Support**
   - During the Spring 2021 semester, the Jacobs Hall Makerspace will be closed to all non-essential community members, but the design team can fabricate items for the

campus community on request. Bioinspired Design students can secure priority status in the Jacobs Project Support system by registering. There is no fee to register, but there will be cost for materials. Learn more here, and register here.

2. **Complete General Safety Training**
   - Enroll in the Maker Pass Equipment and Safety bCourses page.
   - On the Maker Pass Equipment and Safety home page read the General Safety training curriculum, take the online quiz, and complete the three General Safety Agreements (1, 2, 3).
3. **Download Cura 4.8**
   - Visit the Ultimaker 3D Printer Landing Page. This page has information about preparing and printing files at Jacobs Makerspace. Although you won't be printing in-person this semester, we still think it is valuable to see how 3D printing software works.
   - Scroll down to the 'Download' subsection where you'll find a link to Ultimaker's site. This link will take you to Ultimaker's software page where you'll find a blue 'Download for free' button. Click on the button and choose the Windows, Mac, or Linux option based on your computer's operating software.
     NOTE: There should be a 'Download Now' button to click when you select which version of the software you want. If you don't see it, try opening the page in a different web browser.
   - If you are using a Windows computer, it is recommended you download Bonjour for an easy network connection. This download link is also mentioned and available in the training module.
4. **Ultimaker 3D Printer Training Module and Quiz**
   - While staying in the training manual, continue to read through the training curriculum and take the online quiz.
   - The training will guide you through all the steps on how to get Cura up and running for your 3D print.
     NOTE: You may see some discrepancies between the screenshots included in the tutorial and interface that you download from Ultimaker's website. The screenshots are from an earlier version of Cura, but the tutorial still contains important information.
   - Make sure to download the Project File for the Ultimaker printers in Step 2 of the training. This ensures your print is compatible with the Jacobs Ultimakers.
   - You may take the quiz as many times as needed to pass.

IN-CLASS HANDS ON TRAINING PROCEDURES (FRI JANUARY 29)

During class, your GSI will walk you through the steps of opening Cura, loading the splint file, preparing it for printing, and submitting the prepared job to the Makerfleet system. You may find it easier to just watch the GSI's shared screen, but feel free to follow along on your own computer.
NOTE: You will not actually print the file in or outside of class. Design specialists at Jacobs Hall have printed your finger splint in advance. We are using this as a training opportunity to get some hands-on experience with Cura and the Makerfleet system in case you need to use these programs later in the semester.

1. Download the prosthetic finger splint file here.

2. Open Cura and learn how to prepare files for printing.

- After opening Cura, click on the folder icon. First, load the Project File that you downloaded from the bCourses training.
- Next, load the prosthetic finger splint. You don't need to change any of the settings.

- Click the blue button that says ‘Slice.’
- After Cura finishes the process, click the button that says ‘Save to File’ and save the gcode file somewhere you can easily find it.

3. Go to the Jacobs Project Support page and learn how to use the Makerspace this semester.

- Click the Ultimakers 3D Printers icon and learn more about Google form requests.

ASSIGNMENT (DUE THURSDAY FEBRUARY 11)

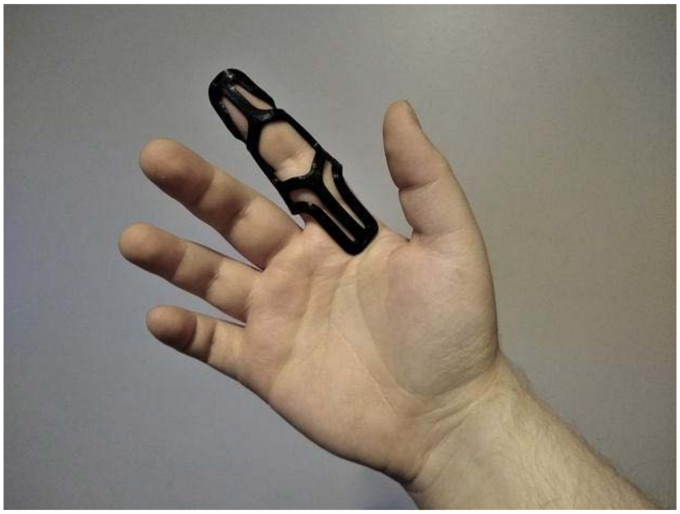

Watch a video of the finger splint being printed in CiBER.

Now that you’ve successfully 3D printed the splint, you can mold it to your own finger. Watch a video of how it is done before reading the instructions and doing it yourself. The instructions for molding the splint are adapted from FFFTechnology.

1. Obtain your 3D printed finger splint and gecko adhesive testing kit.

- If you are away from Berkeley or otherwise unable to visit campus, check your mail for your splint.
- If you are in Berkeley and able to do so, pick up your 3D printed splint and gecko adhesive test kit from CiBER.
- **NOTE:** The gecko adhesive test kit was packaged with the finger splint to consolidate trips, but you will not use it for the finger splint project. You should save it for the first team design project.

2. Coordinate with your team to assign each team member a finger to try their splint on.

- Each member of your team should try to mold their splint to a different finger. After you’ve molded the splint to your finger, you’ll submit a photo of yourself wearing the splint, and for each team we want to see each finger (thumb, index, middle, ring, and pinky) represented in your pictures.

3. Mold the splint to your finger.

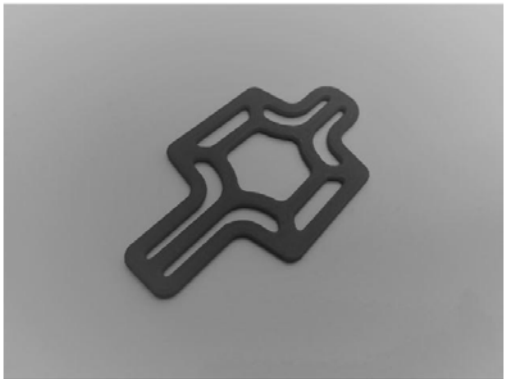

- Before you begin, examine the 3D printed splint. You will place it against the inside of your finger. The long end will extend over your knuckle and the short end will extend to the tip of your finger. The wings will curl around the sides of your finger.
- Obtain a bowl of hot water, a bowl of cold water, a pair of tongs, and your 3D printed splint. If you are sensitive to temperature changes or worried about burning yourself, consider also obtaining a cloth to wrap around the finger you plan to splint.
- Drop the splint into the bowl of hot water and let it soften. The material will soften quickly, but there is no need to rush.
- Once the splint has softened, remove it from the bowl of hot water using your tongs. If the water was especially hot and you don’t have a cloth wrap, place the splint down on the

table for a second or two before placing it against your finger, but don't wait long because the material will cool and harden quickly.

- While the splint is still soft, place it against the inside of the finger you've chosen to immobilize. Quickly bend the wings around the sides of your finger. Tighten the splint so that it doesn't fall off your finger, but remember to leave it loose enough that you can pull the splint off afterwards.
- Place your finger with the splint still on into the bowl of cold water.
- If you move too slowly or the splint doesn't mold properly for some other reason, you can put the splint back in the hot water, carefully remove it when soft, flatten it, and start the process over again.
- You can also watch how this is done in the linked video above.

**** REMEMBER, SAFETY FIRST**
Avoid burning yourself with hot water by using tongs.

4. Take a selfie with your 3D printed finger splint molded to the finger your team has assigned to you and submit it to the bCourses Assignment

- Please submit a photo that includes you and your finger in your molded 3D printed finger splint.

TEAM DISCUSSION (FRIDAY FEBRUARY 12)
Now that you've each been through the 3D printing process and had a chance to mold a splint to your own finger, take some time to discuss the process with your team. To guide you in your discussion, we've provided several questions below. Answer the questions below together as a team, and then we'll discuss them as a class.

For discussion:
1. Was the splint more effective for some team members than others? What did you notice about how the splint fit on different people? On different fingers?

2. Are there any limitations that you can think of for this splint design? Could you improve on this splint design to address specific finger injuries? Try to give an example.

3. If you actually had a finger injury, what are some important steps you might take to get the most out of a 3D printed splint? Can you think of other maker tools that might help?

4. Could this finger splint design be modified for use in other injuries? Could you think of more effective designs for immobilizing a thumb? As a group, provide a sketch of a splint that could effectively immobilize a thumb.

OFFICE HOURS
GSIs will be available during office hours to help you if you run into any issues with your splint or if you want to discuss more about 3D printing.

SELECTED READINGS
Ultimate Beginner's Guide to 3D Printing: https://www.makeuseof.com/tag/beginners-guide-3d-printing/

Link URLs

- Maker Pass:
  https://jacobsaccess.ist.berkeley.edu/jps/signup

- Jacobs Maker Space Safety bCourse:
  https://bcourses.berkeley.edu/courses/1353091

-Bonjour software:
  https://support.apple.com/downloads/bonjour_for_windows

- In-Class Print File:
  https://bcourses.berkeley.edu/files/79511478/download?download_frd=1

Supplement S9 – Team Collaborative Plan

Build Your Collaborative Plan

At a team meeting, discuss your Goals (small g and BIG G), Roles, Procedures & Relationships, using the prompts you see below. Document your discussion and agreements by filling in the Collaborative Plan Template, removing each prompt as you respond to it, or using the blank template using the one below as a guide. The template expands: use as many pages as you need.

**Team Name: _______________________________________ Date: __________________**	
**Goals**	• What are the **personal goals (small g) of each member on this team**? (Team Member #1,#2, #3, etc: 1 or more goals/ name)<br>• What is the **Project GOAL (big G)** we're all committed to achieve together?<br>• Is our **Project Goal scaled** to our resources (dreams, materials, skills, differences, etc.) and constraints (assignment, time, skills, etc.)<br>• What are the **metrics for success** for what we're producing?
**Roles**	• **Who** is responsible for **which deliverables?**<br>• **Which deliverables** that require **collaboration, subgroups & individual work? Who** does each person **depend upon to succeed?**<br>• Do we need a **project manager** to coordinate?<br>• What are the **deliverables each person** is accountable to produce?
**Procedures**	• **Decision Making -** What **process** shall we use: consensus, majority rules, deference to expert, default to the loudest, or?<br>• **Effective Meetings -** Focus on key, timely decisions together vs. status/update (offline);<br>• **Meeting roles:** scribe, facilitator, time keeper<br>• **Communication - FTF:** frequency, time, location**; type of technology:** (Googledocs, Hangout, etc.); **expectations for responsiveness; 'best time to work'** (AM, PM, weekends?)
**Relationships**	• **Team Diversity** – What is the diversity on our team? Disciplines to tap for solutions; individual earning styles for the stages of invention; overall team learning style strengths and places to supplement; cultural backgrounds , work experience, dreams to leverage for scope & impact of goals, new roles, better procedures; languages for more diverse customer set, bigger market;<br>• **Listening** – Notice my binary thinking, auto-rankism, and go beyond it.<br>• **Team Name**–What's a **team name** that captures who we are and what we're going to do?

Team Name: ____ Date: __________________	
**Goals**	
**Roles**	
**Procedures**	
**Relationships**	

Supplement 10 - Connections – Sharing Opportunities and Interests with a Community

Individual students or Teams will add a ***Connection link*** *via* a weblink provided in the relevant bCourses Assignment to share their findings of interests as a connection. These URLs can include relevant courses on campus; links to campus organizations, clubs, institutes and competitions interested in the subject of the course; biological discoveries and bioinspired designs from news and journals; global research, centers, and institutes; and internships, public service, and research opportunities. Students submit a brief description and upload 1-3 images when possible (.jpg; <5MB). Students view the prior week's submissions and LIKE any and all of the Connections that they find interesting or helpful. They will not be graded based on the number of Connections they LIKE, but please have them choose at least one each week.

Connection Title Formatting Help

Please follow the below structure when forming your Connection Titles. *Please click on any of the examples below to view full Connection examples.*

Biological Discoveries: Type ("News" or "Paper"), Title

ex: Paper, Adhesive force of a single gecko foot-hair.

Competition: Competition Name, Sponsor, Location ("Global" is a valid option)

ex: Biodesign Challenge, Mult. Sponsors, Global.

Course: Course Name, Course Number, Institution

ex: BioInspired Design, Integrative Biology, INTEGBI 32, UC Berkeley.

Internship: Sponsor Name, Location ("Global" is a valid option)

ex: Maker Studio SF, Local

Organization: Name, Location ("Global" is a valid option)

ex: Center for Nature Inspired Engineering, Global.

Outreach Opportunity: Name, Location ("Global" is a valid option)

ex: Bay Area Scientists in Schools (BASIS), Local.

Researcher: Name, Primary Focus, Institution

ex: Robert J. Full, Int. Biol, UC Berkeley

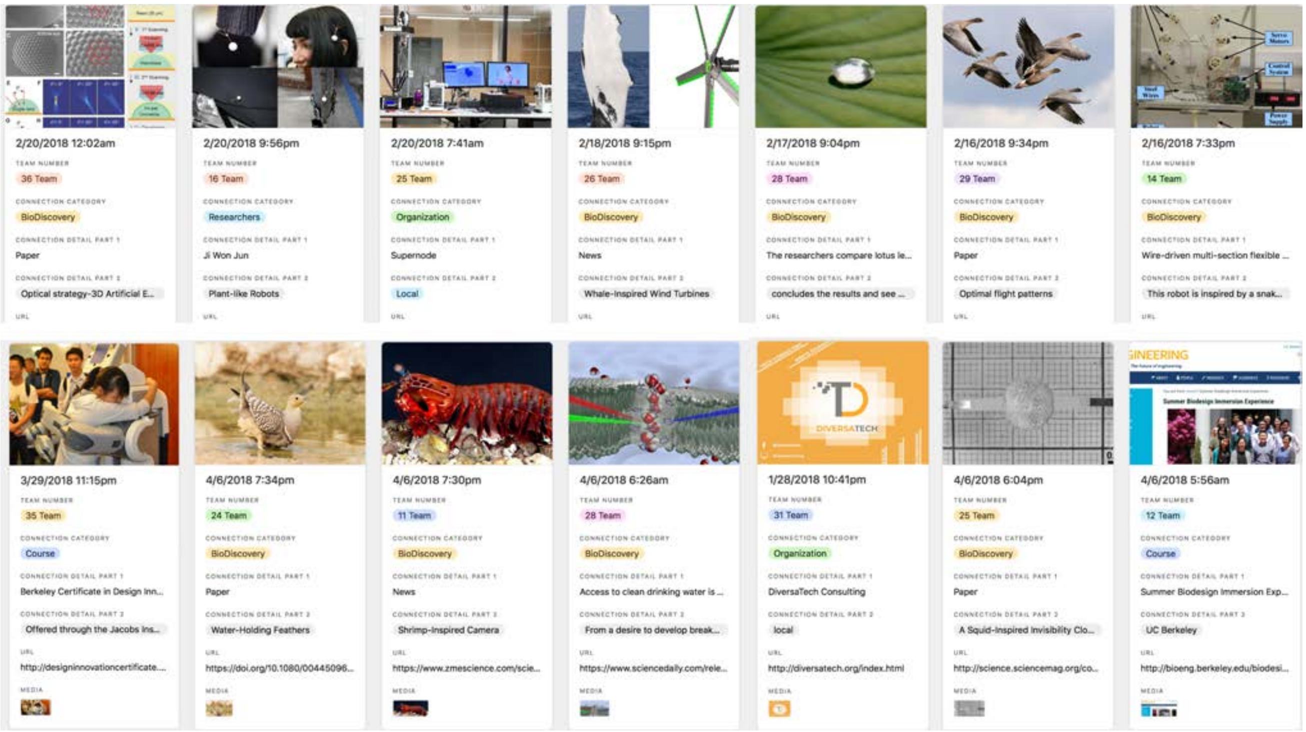

2/20/2018 12:02am
TEAM NUMBER
36 Team
CONNECTION CATEGORY
BioDiscovery
CONNECTION DETAIL PART 1
Paper
CONNECTION DETAIL PART 2
Optical strategy-3D Artificial E...
URL
2/20/2018 9:56pm
TEAM NUMBER
16 Team
CONNECTION CATEGORY
Researchers
CONNECTION DETAIL PART 1
Ji Won Jun
CONNECTION DETAIL PART 2
Plant-like Robots
URL
2/20/2018 7:41am
TEAM NUMBER
25 Team
CONNECTION CATEGORY
Organization
CONNECTION DETAIL PART 1
Supernode
CONNECTION DETAIL PART 2
Local
URL
2/18/2018 9:15pm
TEAM NUMBER
26 Team
CONNECTION CATEGORY
BioDiscovery
CONNECTION DETAIL PART 1
News
CONNECTION DETAIL PART 2
Whale-Inspired Wind Turbines
URL
2/17/2018 9:04pm
TEAM NUMBER
28 Team
CONNECTION CATEGORY
BioDiscovery
CONNECTION DETAIL PART 1
The researchers compare lotus le...
CONNECTION DETAIL PART 2
concludes the results and see ...
URL
2/16/2018 9:34pm
TEAM NUMBER
29 Team
CONNECTION CATEGORY
BioDiscovery
CONNECTION DETAIL PART 1
Paper
CONNECTION DETAIL PART 2
Optimal flight patterns
URL
2/16/2018 7:33pm
TEAM NUMBER
14 Team
CONNECTION CATEGORY
BioDiscovery
CONNECTION DETAIL PART 1
Wire-driven multi-section flexible ...
CONNECTION DETAIL PART 2
This robot is inspired by a snak...
URL
3/29/2018 11:15pm
TEAM NUMBER
35 Team
CONNECTION CATEGORY
Course
CONNECTION DETAIL PART 1
Berkeley Certificate in Design Inn...
CONNECTION DETAIL PART 2
Offered through the Jacobs Ins...
URL
http://designinnovationcertificate....
MEDIA
4/6/2018 7:34pm
TEAM NUMBER
24 Team
CONNECTION CATEGORY
BioDiscovery
CONNECTION DETAIL PART 1
Paper
CONNECTION DETAIL PART 2
Water-Holding Feathers
URL
https://doi.org/10.1080/00445096...
MEDIA
4/6/2018 7:30pm
TEAM NUMBER
11 Team
CONNECTION CATEGORY
BioDiscovery
CONNECTION DETAIL PART 1
News
CONNECTION DETAIL PART 2
Shrimp-Inspired Camera
URL
https://www.zmescience.com/scie...
MEDIA
4/6/2018 6:26am
TEAM NUMBER
28 Team
CONNECTION CATEGORY
BioDiscovery
CONNECTION DETAIL PART 1
Access to clean drinking water is ...
CONNECTION DETAIL PART 2
From a desire to develop break...
URL
https://www.sciencedaily.com/rele...
MEDIA
DIVERSATECH
1/28/2018 10:41pm
TEAM NUMBER
31 Team
CONNECTION CATEGORY
Organization
CONNECTION DETAIL PART 1
DiversaTech Consulting
CONNECTION DETAIL PART 2
local
URL
http://diversatech.org/index.html
MEDIA
4/6/2018 6:04pm
TEAM NUMBER
25 Team
CONNECTION CATEGORY
BioDiscovery
CONNECTION DETAIL PART 1
Paper
CONNECTION DETAIL PART 2
A Squid-Inspired Invisibility Clo...
URL
http://science.sciencemag.org/co...
MEDIA
GINEERING
Summer Biodesign Immersion Experience
4/6/2018 5:56am
TEAM NUMBER
12 Team
CONNECTION CATEGORY
Course
CONNECTION DETAIL PART 1
Summer Biodesign Immersion Exp...
CONNECTION DETAIL PART 2
UC Berkeley
URL
http://bioeng.berkeley.edu/biodesi...
MEDIA
