# Supplementary material for: Eyes Toward Tomorrow Program Enhancing Collaboration, Connections, and Community Using Bioinspired Design
Source: Integr Comp Biol. 2021 Aug 30;61(5):1966–80. doi: 10.1093/icb/icab187 (PMC8699102; doi:10.1093/icb/icab187)
Supplement: icab187_Supplemental_Files [file icab187_supplemental_files.zip › icb-2021-0200-File010.pdf]

Supplement S11 – Midterm Exam 2021 Spring

Select Your Own Paper, Do a Discovery Decomposition and Analogy Check, and Link to Lecture Concepts/ There are 135 points on this exam

Search for a biology research publication that can inspire a design which interests you. Each student will hand-in:

A research publication with a biological discovery not yet translated to a device. As a reminder from our first discussion meeting, Google Scholar and the Web of Science are all great places to start looking for research publications along with targeted journals (see Search_strategies.pdf). A good place to start checking if the discovery has been translated are the papers citing the original publication.

Question 1. 5 pts. I selected the following research publication (Upload .pdf):

Nirody JA, Jinn J, Libby T, Lee TJ, Jusufi A, Hu DL, Full RJ. Geckos race across the water's surface using multiple mechanisms. Current Biology. 2018 Dec 17;28(24):4046-51.

Question 2. 15 pts. **Discovery Decomposition**. Breakdown your publication using the Discovery Decomposition approach discussed in class and lecture (example below). Produce your own flow diagram using the structure below as we did for the gecko publications (use Discovery_decomp_v1.pptx template).

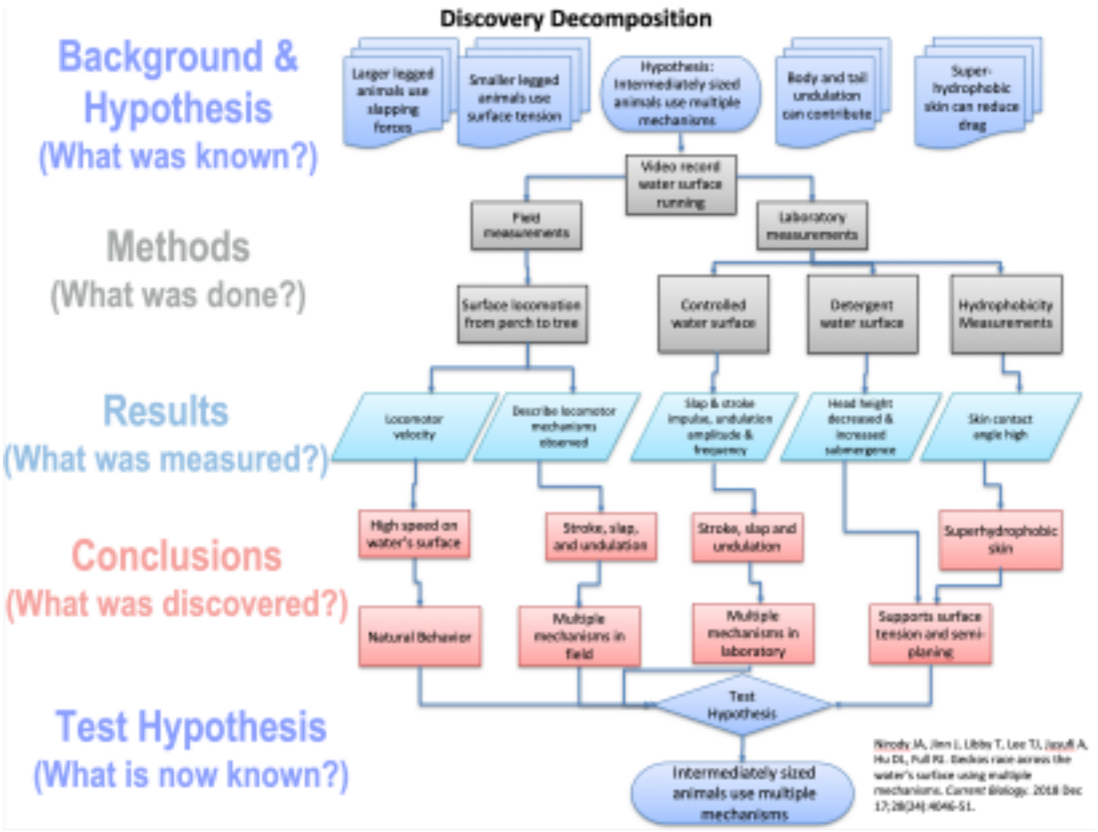

Question 3. 15 pts. **Analogy Check**. Create an Analogy Check Table as we did for both gecko publications using the approach discussed in class and lecture. Produce your table using the structure below and suggest a novel design and invention name (use the Analogy_check_v1.pptx template). In the first column, fill in as much as possible from what you have learned from your Discovery Decomposition. In the third column, Design Problem, name your Design! Make sure you fill out your best guesses about your new design. In the middle column indicate whether the parameters are Similar (translation possible, green for go), Different (need more experimentation, red for stop), Uncertain (need more information, gray) or N/A (not applicable). Use the following file as a template to fill in. Analogy_check_v1.xlsx

Design Solution	Analogy Check	Design Problem
**House gecko**		**ROWbot (RunOnWater)**
Function (What does system or organism do?)		Function (What do you want system to do?)
Runs on water with four legs and tail using multiple mechanisms	Similar	Run on water with four legs and tail using multiple mechanisms for rapid hazard detection and search-and-rescue.
**Structural Components** (What is structure or organization of system?)		**Structural Components** (What can the structure be?)
Bone, muscle, tendon and ligament units	Different	Flexible feet from variable stiffness polymers Carbon fiber body and tail
Superhyrdophobic skin	Uncertain	Superhyrdophobic skin
**Operating Environment** (Where?)		**Operating Environment** (Where?)
Water's surface	Similar	Water's surface
**Size** (What is size?)		**Size** (What size needed?)
Body mass 6 grams and 100 cm long	Different	Body mass 20 grams
100 cm long	Similar	100 cm long
**Mechanisms** (How does system work?)		**Mechanisms** (How do you want the system to work?)
Feet for slapping, stroking, and padding.	Similar	Slapping, stroking, and padding feet
Spine and tail for undulatory propulsion.	Different	Only tail for undulatory propulsion.
Superhyrdophobic skin for semi-planing	Uncertain	Superhyrdophobic skin for semi-planing
**Characteristics/Specification** (Which are distinguishing?)		**Characteristics/Specification** (What are your specifications?)
Leg stroke impulses to lift front end	Similar	Leg stroke impulses to lift front end
Undulatory spine and tail.	Different	Undulatory tail.
Superhyrdophobic skin for lift	Similar	Superhyrdophobic skin for lift
**Performance Criteria** (How well does system work?)		**Performance Criteria** (How well must the system work?)
High velocity (>50 cm/s)	Similar	High velocity (>50 cm/s)
Robust to sinking failure	Similar	Robust to sinking failure
**Constraints** (What compromises system?)		**Constraints** (Can compromises be removed?)
History. Four legged ancestors	Uncertain	Can remove hind legs
Multifunctionality. All parts have many functions (e.g. terrestrial locomotion and climbing)	Uncertain	Can simplify structures for water running.

Linking your paper to lecture concepts.

You are the biologist in a design team that includes an engineer, an applied mathematician, and a start-up company with business managers and entrepreneurs. Please take the role of the author of the biology paper. Answer the team's questions based on your publication, proposed design invention, and Lectures 1-7.

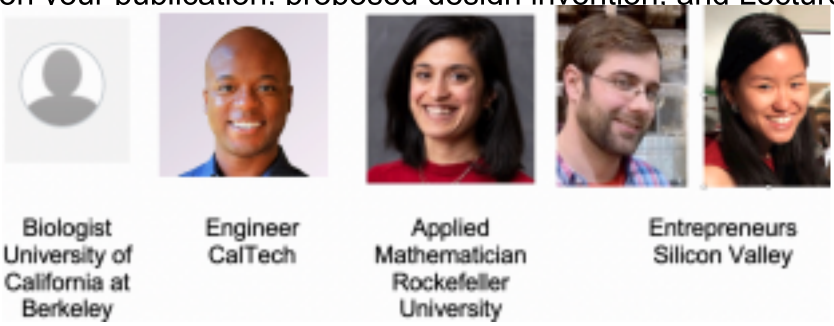

Question 4. 10 pts. The name of your start-up company is _Robofeats_______ .

Question 5. 10 pts. **BioDiscovery.** Your team asks:
What hypotheses are you testing in your publication?

We tested the hypothesis that geckos race along the water's surface by using a combination of strategies arising from slapping and stroking, undulation, and surface tension forces.

Question 6. 10 pts. **BioDiscovery.** What was your paper's main principle or discovery that you will translate to your novel device?

To move rapidly along the air-water interface, intermediate sized animals can employ both the slapping/stroke forces used by large animals and the surface tension forces used by small animals.

Question 7. 10 pts. **Bioinspired Design Process.** Your team asks you to:
1. Express your design analogy in a simple logic statement (e.g. Premise…, Therefore…, Conclusion).

Premise
An intermediate sized gecko and robot are similar because they can both run rapidly on surfaces.
Premise
An intermediate sized gecko can run rapidly over the water's using both slap/strokes forces and surface tension forces.
Therefore,
Conclusion
An intermediate sized robot might be designed to run rapidly over the water's using both slap/strokes forces and surface tension forces.

Question 8. 10 pts. **Bioinspired Design Process.** Provide advice to the team on the bioinspired design process? Select one concept and/or principle discussed in the BioDesign lecture. Explain the concept and/or principle. State how it is important with respect to your discovery and the translation process.

Checking Solution Driven Analogies. You tell your team that it is critical to check your analogy carefully with respect to all major parameters, because one may invalidate the analogy and hinder translation. In particular, our team must be very careful given the major differences in

the materials that comprise the foot, leg, and tail. However, just because an analogy is uncertain or different, does not mean the process is failed. In fact, less certain analogies can lead to new research areas for the team.

Question 9. 10 pts. **BioConstraints.** Your team asks whether the organism or system that you selected has been compromised or constrained?
Select one concept and/or principle discussed in the BioConstraint lecture. Explain the concept and/or principle. State how it is important with respect to your discovery and the translation process.

Functional and Evolutionary Constraints. Yes, gecko feet, body, and tail serve multiple functions in addition to running on water. These include running on the ground, climbing, feeding, mating, and reproduction. Geckos have evolved in terrestrial and arboreal environments. Therefore, their history has shaped foot, body, and tail.

Question 10. 10 pts. **BioSelection.** Your team asks why you selected this organism or system?
Select one concept and/or principle discussed in the BioSelection lecture. Explain the concept and/or principle. State how it is important with respect to your discovery and the translation process.

Unique Solutions. Quadrupedal water running geckos offer a unique solution to traversing the air-water interface. It is potential advantageous because the robot design could benefit from carrying a greater payload that a biped.

Question 11. 10 pts. **BioScaling.** Your team asks you how easily that the team might increase or decrease the size of the design.
Select one concept and/or principle discussed in the BioScaling lecture. Explain the concept and/or principle. State how it is important with respect to your discovery and the translation process.

Properties of Fluids Scale with Size. Considering size is absolutely critical for interfacial locomotion. The relative importance of gravitational and inertial forces (Bond #) versus surface forces (Weber #) depends on size. Making the robot larger will decrease surface tension forces, but increase inertial forces (slap/stroke). Making the robot smaller will increase surface tension forces, but reduce inertial forces.

Question 12. 10 pts. **BioComplexity.** Your business partners are fearful that your organism or system is just too complex to copy.
Select one concept and/or principle discussed in the BioComplexity lecture. Explain the concept and/or principle. State how it is important with respect to your discovery and the translation process.

Develop Simple Models. You tell your business team that the last thing you want to do is copy the gecko. It is too complex with far too many dimensions. Instead, you will work with your applied mathematician to create a simple model or template. The template will provide the

simplest model with the fewest parameters that can serve to translate the principle to your engineer to realize your robot design idea.

Question 13. 10 pts. **Marketing.** Write a short (<300 words) sales pitch for your final product that can be the beginning of an ad. Please include the potential societal benefit.

ROWbot **(R**un**O**n**W**ater**)** is the only robot of its type that can rapidly enter flooded disaster areas to find individuals trapped in rubble resulting from an earthquake, explosion, tornado, or hurricane. ROWbot can rapidly enter areas of potential danger to humans through sewer or water delivery systems to collect data for assessing a chemical, biological, or nuclear hazard. FEMA approved. If you need Morebot, then you need ROWbot from Robofeats Inc. Contact us for a demonstration.
