# Supplementary material for: Eyes Toward Tomorrow Program Enhancing Collaboration, Connections, and Community Using Bioinspired Design
Source: Integr Comp Biol. 2021 Aug 30;61(5):1966–80. doi: 10.1093/icb/icab187 (PMC8699102; doi:10.1093/icb/icab187)
Supplement: icab187_Supplemental_Files [file icab187_supplemental_files.zip › icb-2021-0200-File011.pdf]

Supplement S12 - 3D Printing Makerspace Activities Survey

Makerspace Prosthetic Hand Activity Survey

Please fill out the course feedback survey to the best of your ability by Monday Feb. 24th. The questionnaire is anonymous and will be used to improve the course for the future.
Respond to the statements by indicating (1) strongly disagree, (2) disagree, (3) undecided, (4) agree, or (5) strongly agree and fill in the Boxes when asked.

1. I was unfamiliar with Makerspace activities before this assignment.

	1	2	3	4	5	
Strongly Disagree						Strongly Agree

2. I was unfamiliar with 3D printing before this assignment.

	1	2	3	4	5	
Strongly Disagree						Strongly Agree

3. I feel more comfortable about using the Makerspace equipment after this assignment.

	1	2	3	4	5	
Strongly Disagree						Strongly Agree

4. I am more interested in learning about other Makerspace equipment (e.g. laser cutting) after this assignment.

	1	2	3	4	5	
Strongly Disagree						Strongly Agree

5. I feel more confident in learning about other Makerspace equipment with the help of staff after this assignment.

	1	2	3	4	5	
Strongly Disagree	⬭	⬭	⬭	⬭	⬭	Strongly Agree

6. The Discussion activity of downloading and explaining the 3D printing software was useful.

	1	2	3	4	5	
Strongly Disagree	⬭	⬭	⬭	⬭	⬭	Strongly Agree

7. How would you improve learning how to use the 3D printing software?

8. The 3D printing presentation by the Jacob's staff at the printer was useful.

	1	2	3	4	5	
Strongly Disagree	⬭	⬭	⬭	⬭	⬭	Strongly Agree

9. How would you improve learning how to do 3D printing?

10. The handout was useful in explaining the whole 3D printing Makerspace activity.

	1	2	3	4	5	
Strongly Disagree	⬭	⬭	⬭	⬭	⬭	Strongly Agree

11. The submission of a selfie was a sufficient method to show that you 3D printed a finger.

	1	2	3	4	5	
Strongly Disagree	⬭	⬭	⬭	⬭	⬭	Strongly Agree

12. I 3D printed my finger for this project in (selectone)

Mark only one oval.

- ⬭ CiBER
- ⬭ Jacobs Hall

13. The queue system used for 3D printing was effective at facilitating the use of the Makerspace equipment.

	1	2	3	4	5	
Strongly Disagree	⬭	⬭	⬭	⬭	⬭	Strongly Agree

14. How would you improve the queue system for facilitating 3D printing?

____

____

____

____

____

15. The 3D printing of a finger was not a technically difficult exercise.

	1	2	3	4	5	
Strongly Disagree	⬭	⬭	⬭	⬭	⬭	Strongly Agree

16. How would you improve the activity of 3D printing your finger?

____

____

____

____

____

17. The assembly of the prosthetic hand during Discussion was useful and fun.

	1	2	3	4	5	
Strongly Disagree	⬭	⬭	⬭	⬭	⬭	Strongly Agree

18. Discussion questions for the prosthetic hand assembly helped me think about various aspects of bio-inspired design that I haven't thought about.

	1	2	3	4	5	
Strongly Disagree	◯	◯	◯	◯	◯	Strongly Agree

19. How would you improve the assembly experience of your team's prosthetic hand?

____

____

____

____

____

20. Your team was interested in completing the assembly of your hand if you did not finish.

	1	2	3	4	5	
Strongly Disagree	◯	◯	◯	◯	◯	Strongly Agree

21. The early Makerspace activity should be continued in the future to learn about 3D printing.

	1	2	3	4	5	
Strongly Disagree	◯	◯	◯	◯	◯	Strongly Agree

22. Please add any other comments, questions, or concerns.

____

____

____

____

____

Makerspace Prosthetic Finger Splint Activity Survey

Please fill out the course feedback survey to the best of your ability by Monday Feb. 24th. The questionnaire is anonymous and will be used to improve the course for the future.
Respond to the statements by indicating (1) strongly disagree, (2) disagree, (3) undecided, (4) agree, or (5) strongly agree and fill in the Boxes when asked.

1. I was unfamiliar with Makerspace activities before this assignment.

Mark only one oval.

	1	2	3	4	5	
Strongly Disagree	◯	◯	◯	◯	◯	Strongly Agree

2. I was unfamiliar with 3D printing before this assignment.

Mark only one oval.

	1	2	3	4	5	
Strongly Disagree	◯	◯	◯	◯	◯	Strongly Agree

3. I am more familiar with Makerspace equipment after this assignment.

Mark only one oval.

	1	2	3	4	5	
Strongly Disagree	◯	◯	◯	◯	◯	Strongly Agree

4. I am more interested in learning about other Makerspace equipment (e.g. laser cutting) after this assignment.

Mark only one oval.

	1	2	3	4	5	
Strongly Disagree	◯	◯	◯	◯	◯	Strongly Agree

5. I feel more confident in learning about other Makerspace equipment with the help of staff after this assignment.

Mark only one oval.

	1	2	3	4	5	
Strongly Disagree	◯	◯	◯	◯	◯	Strongly Agree

6. The information in the handout about downloading 3D printing software was useful.

Mark only one oval.

	1	2	3	4	5	
Strongly Disagree	◯	◯	◯	◯	◯	Strongly Agree

7. The Discussion activity of downloading and explaining the 3D printing software was useful.

Mark only one oval.

	1	2	3	4	5	
Strongly Disagree	◯	◯	◯	◯	◯	Strongly Agree

8. How would you improve learning how to use the 3D printing SOFTWARE?

9. The video on bCourses that showed the 3D printing process was useful.

Mark only one oval.

	1	2	3	4	5	
Strongly Disagree	◯	◯	◯	◯	◯	Strongly Agree

10. How would you improve learning the process of how to USE a 3D printer?

11. The handout was useful in explaining how to mold the finger splint.

Mark only one oval.

	1	2	3	4	5	
Strongly Disagree	◯	◯	◯	◯	◯	Strongly Agree

12. Molding the finger splint was a fun and useful activity.

Mark only one oval.

	1	2	3	4	5	
Strongly Disagree	⬭	⬭	⬭	⬭	⬭	Strongly Agree

13. Coordinating which finger each teammate would mold was a good way to interact with teammates for the first time.

Mark only one oval.

	1	2	3	4	5	
Strongly Disagree	⬭	⬭	⬭	⬭	⬭	Strongly Agree

14. The submission of a selfie was a sufficient method to show that you received and molded your finger splint.

Mark only one oval.

	1	2	3	4	5	
Strongly Disagree	⬭	⬭	⬭	⬭	⬭	Strongly Agree

15. How would you improve the activity of MOLDING the finger splint?

16. The in-class discussion of the finger splint (advantages, disadvantages, modifications, etc.) was fun and useful.

Mark only one oval.

	1	2	3	4	5	
Strongly Disagree	⬭	⬭	⬭	⬭	⬭	Strongly Agree

17. Discussion questions for the finger splint activity helped me think about various aspects of bio-inspired design that I haven't thought about.

Mark only one oval.

	1	2	3	4	5	
Strongly Disagree	⬭	⬭	⬭	⬭	⬭	Strongly Agree

18. How would you improve the in-class DISCUSSION of the finger splint activity?

____

____

____

____

____

19. You attempted to re-mold the finger splint even after successfully completing the activity.

Mark only one oval.

	1	2	3	4	5	
Strongly Disagree	⬭	⬭	⬭	⬭	⬭	Strongly Agree

20. This early Makerspace activity should be continued in the future to learn about 3D printing.

Mark only one oval.

	1	2	3	4	5	
Strongly Disagree	◯	◯	◯	◯	◯	Strongly Agree

21. Please add any other comments, questions, or concerns.

____

____

____

____

____

Please indicate how true each statement is for you.

22. I think what we are learning in this course is important.

Mark only one oval.

	1	2	3	4	5	6	7	
Not at all true	◯	◯	◯	◯	◯	◯	◯	Very true

23. I want to study technology and belong to a creative community because I want to make a contribution to society.

Mark only one oval.

	1	2	3	4	5	6	7	
Not at all true	◯	◯	◯	◯	◯	◯	◯	Very true

24. Learning about science and technology will help me become the person I want to be.

Mark only one oval.

	1	2	3	4	5	6	7	
Not at all true	◯	◯	◯	◯	◯	◯	◯	Very true

This content is neither created nor endorsed by Google.

Google Forms

Supplement S13 - Anonymous Design Project Survey

Please fill out the course feedback survey to the best of your ability. The questionnaire is anonymous and will be used to improve the course for the future.

Respond to the statements by indicating (1) strongly disagree, (2) disagree, (3) undecided, (4) agree, or (5) strongly agree and fill in the Boxes when asked.

NOTE: This survey is anonymous.

* Required

1. The Discovery Decomposition Approach helped to breakdown and understand the scientific papers. *

Mark only one oval.

	1	2	3	4	5	
Strongly Disagree	◯	◯	◯	◯	◯	Strongly Agree

2. The Analogy Check Approach helped to ask questions about how well your bioinspiration translates to your trial design. *

Mark only one oval.

	1	2	3	4	5	
Strongly Disagree	◯	◯	◯	◯	◯	Strongly Agree

3. There were the right number of team trial design projects (GSA, Robot, and Final). *

Mark only one oval.

	1	2	3	4	5	
Strongly Disagree	⬭	⬭	⬭	⬭	⬭	Strongly Agree

Question: If you could add a design project what would it include?

____

____

____

____

____

4. Uploading assignments to bCourses was effective. *

Mark only one oval.

	1	2	3	4	5	
Strongly Disagree	⬭	⬭	⬭	⬭	⬭	Strongly Agree

Question: How might we improve the use of bCourses?

____

____

____

____

____

5. The Gecko-inspired Synthetic Adhesive Design Project was a valuable exercise. *

Mark only one oval.

	1	2	3	4	5	
Strongly Disagree	◯	◯	◯	◯	◯	Strongly Agree

Question: How might we improve the Gecko-inspired Synthetic Adhesive Design Project?

6. The DASH Robot Design Project was a valuable exercise. *

Mark only one oval.

	1	2	3	4	5	
Strongly Disagree	◯	◯	◯	◯	◯	Strongly Agree

Question: How might we improve the DASH Robot Design Project?

7. Using the Box Folder facilitated team interaction. *

Mark only one oval.

	1	2	3	4	5	
Strongly Disagree	◯	◯	◯	◯	◯	Strongly Agree

8. The Collaborative Plan helped your team organize to develop your trial designs. *

Mark only one oval.

	1	2	3	4	5	
Strongly Disagree	◯	◯	◯	◯	◯	Strongly Agree

Question: How might we better facilitate team interactions?

9. Having each team member evaluate other team members encourages participation. *

Mark only one oval.

	1	2	3	4	5	
Strongly Disagree	◯	◯	◯	◯	◯	Strongly Agree

Question: What other approaches might encourage participation?

10. The Final Design Project would benefit from more directed workshops on the Design Process and Design Thinking. *

Mark only one oval.

	1	2	3	4	5	
Strongly Disagree	◯	◯	◯	◯	◯	Strongly Agree

11. The Final Design Project would benefit from more directed workshops on the use of software important for maker equipment in Jacobs. *

Mark only one oval.

	1	2	3	4	5	
Strongly Disagree	◯	◯	◯	◯	◯	Strongly Agree

12. What would be the one change you would make to improve the Final Design Project? *

This content is neither created nor endorsed by Google.

Google Forms

Supplement S14 - Anonymous Lecture Survey

Please fill out the course feedback survey to the best of your ability. The questionnaire is anonymous and will be used to improve our course for the future.

Respond to the statements by indicating (1) strongly disagree, (2) disagree, (3) undecided, (4) agree, or (5) strongly agree and fill in the Boxes when asked.

NOTE: This survey is anonymous.

* Required

1. The lectures introduced me to new areas and ideas *

Mark only one oval.

	1	2	3	4	5	
Strongly Disagree	⬭	⬭	⬭	⬭	⬭	Strongly Agree

2. The lectures inspired me to consider an aspect of bioinspired design in my future education or career. *

Mark only one oval.

	1	2	3	4	5	
Strongly Disagree	⬭	⬭	⬭	⬭	⬭	Strongly Agree

3. I found the Connections useful. *

Mark only one oval.

	1	2	3	4	5	
Strongly Disagree	⬭	⬭	⬭	⬭	⬭	Strongly Agree 1

Question: How might we improve the way we do Connections?

4. Connections pointed me to new courses, organizations, and/or volunteer opportunities. *

Mark only one oval.

	1	2	3	4	5	
Strongly Disagree	◯	◯	◯	◯	◯	Strongly Agree

5. Connections pointed me to new discoveries that our team used for designs. *

Mark only one oval.

	1	2	3	4	5	
Strongly Disagree	◯	◯	◯	◯	◯	Strongly Agree

6. Liking (voting for) Connections individually further expanded my interests. *

Mark only one oval.

	1	2	3	4	5	
Strongly Disagree	◯	◯	◯	◯	◯	Strongly Agree

7. Question: How might we improve the way we do Connections? *

8. The midterm was representative of the lectures on the process of bioinspired design. *

Mark only one oval.

	1	2	3	4	5	
Strongly Disagree	⬭	⬭	⬭	⬭	⬭	Strongly Agree

9. The class should have a comprehensive final exam. *

Mark only one oval.

	1	2	3	4	5	
Strongly Disagree	⬭	⬭	⬭	⬭	⬭	Strongly Agree

10. bCourses delivered announcements, assignments and files effectively. *

Mark only one oval.

	1	2	3	4	5	
Strongly Disagree	⬭	⬭	⬭	⬭	⬭	Strongly Agree

11. Piazza was an effective tool for communication. *

Mark only one oval.

	1	2	3	4	5	
Strongly Disagree	◯	◯	◯	◯	◯	Strongly Agree

12. Question: How might we improve communication? Is there a better alternative? *

____

____

____

____

____

13. The integration of movies within the lectures was valuable. *

Mark only one oval.

	1	2	3	4	5	
Strongly Disagree	◯	◯	◯	◯	◯	Strongly Agree

14. The Introductory Lecture explained the course objectives and assignments. *

Mark only one oval.

	1	2	3	4	5	
Strongly Disagree	◯	◯	◯	◯	◯	Strongly Agree

15. I found the lectures (Bio-Discovery, -Design, -Constraints, -Scaling, -Selection and -Complexity) explaining the bioinspired design process valuable. *

Mark only one oval.

	1	2	3	4	5	
Strongly Disagree	⬭	⬭	⬭	⬭	⬭	Strongly Agree

Question: How should we improve these lectures? *

16. I found the lectures on BioDesign Case Studies (BioMotion, -Power, -Materials, -Prosthetics, and -Animation) valuable. *

Mark only one oval.

	1	2	3	4	5	
Strongly Disagree	⬭	⬭	⬭	⬭	⬭	Strongly Agree

17. I found the lectures on adhesion helpful for the first design assignment (individual assignment) and the first design project (Gecko-inspired Synthetic Adhesive). *

Mark only one oval.

	1	2	3	4	5	
Strongly Disagree	⬭	⬭	⬭	⬭	⬭	Strongly Agree

18. I found the lectures on BioMotion-Walking and Running and Bio-Control and -Sensing helpful for the second design project (Robot Design). *

Mark only one oval.

	1	2	3	4	5	
Strongly Disagree	⬭	⬭	⬭	⬭	⬭	Strongly Agree

19. I found the guest lectures (BioGreenChem, Biomaterials. BioArchitecture and BioEntrepreneurship) valuable. *

Mark only one oval.

	1	2	3	4	5	
Strongly Disagree	⬭	⬭	⬭	⬭	⬭	Strongly Agree

20. What other lectures topics might we considering adding? *

____

____

____

____

____

21. What was your favorite lecture and why? *

____

____

____

____

____

22. If you could improve one thing about the lectures, what would it be? *

This content is neither created nor endorsed by Google.

Google Forms

Supplement S15 – Team Final Video and Poster Project

Please evaluate yourself and each teammate individually. You will need to Submit another response for each individual.

Responses to these questions will not impact your grade or those of the person named andwill remain confidential.

Responses are part of a research study focusing on how to make the most effective teams.

The Names of your Team members can be found in Files Folder / Team (Groups) SP 2020 / Teams_1.5.pdf

* Required

1. Email address *

2. Evaluator. Your Name: *

3. Name of Team member being evaluated. Your name or teammate's name: *

4. The person named above defined and agreed to a substantive role in our team's collaborative plan. *

Mark only one oval.

	1	2	3	4	5	
Strongly Disagree	◯	◯	◯	◯	◯	Strongly Agree

5. The person named above fulfilled the expectations stated in our team's collaborative plan. *

Mark only one oval.

	1	2	3	4	5	
Strongly Disagree	⬭	⬭	⬭	⬭	⬭	Strongly Agree

6. The person named above surpassed the expectations stated in the team's collaborative plan in helpful ways. *

Mark only one oval.

	1	2	3	4	5	
Strongly Disagree	⬭	⬭	⬭	⬭	⬭	Strongly Agree

7. The person named above likely experienced additional hardships and disruptions limiting their participation. *

Mark only one oval.

	1	2	3	4	5	
Strongly Disagree	⬭	⬭	⬭	⬭	⬭	Strongly Agree

8. The person named above is someone I would definitely want on my team again. *

Mark only one oval.

	1	2	3	4	5	
Strongly Disagree	⬭	⬭	⬭	⬭	⬭	Strongly Agree

9. Please share any additional information you'd like to add about this team member. Provide a suggestion as to something you would like to see them do differently in the future. Offer your comment in the spirit of helping them grow as a team member.

10. Please share a positive comment or characteristic about this team member (something you would like to see them continue doing in the future).

This content is neither created nor endorsed by Google.

Google Forms
