# Supplementary material for: Eyes Toward Tomorrow Program Enhancing Collaboration, Connections, and Community Using Bioinspired Design
Source: Integr Comp Biol. 2021 Aug 30;61(5):1966–80. doi: 10.1093/icb/icab187 (PMC8699102; doi:10.1093/icb/icab187)
Supplement: icab187_Supplemental_Files [file icab187_supplemental_files.zip › icb-2021-0200-File012.pdf]

Supplement S16 Assessment: Bioinspired Design Course, Survey

I. The first part of our assessment involves the psychosocial variables focusing on Science Identity, Science Self-Efficacy, Scientific Community Values, Stereotype Threat, Perceived Stress Scale, Satisfaction with Life Scale, Creativity & Innovation, STEM Enrichment

II. The second part of the assessment focuses on 21st Century Skills. We assess: Scientific Discovery and Translation Process, Interdisciplinary Thinking, and Interdisciplinary Collaboration. The foundation for our formal assessment tools is the BEAR Assessment System (BAS) created by Wilson (2005). BAS is assessment development that guides and supports the design and validation of assessment tools through four building blocks (Fig. 1A). The first building block (a multidimensional Construct Map) concretely identifies variables, described as capabilities, approaches, attitudes and skills that can be observed to assess whether students are meeting goals. We assign five levels of development or success to a given Construct - from Novice to Expert (Fig. 1B). We specify the data necessary to demonstrate each level of success for three main areas.

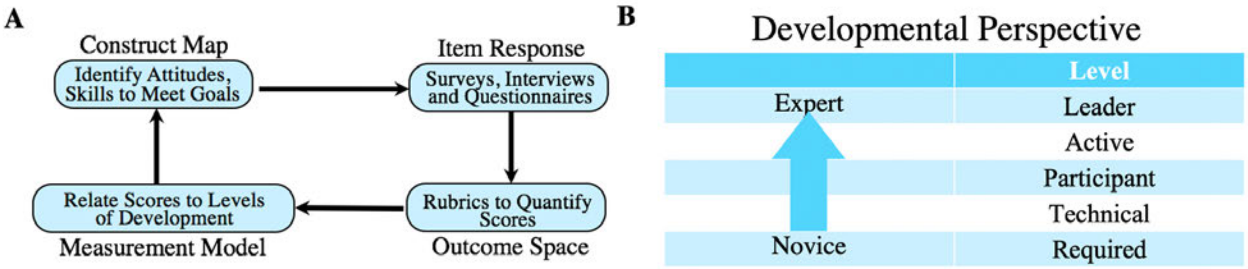

Fig. 1S. Assessment. A. BEAR Assessment System cycle. B. Levels of development for a construct.

We designed our multidimensional construct map and created the second building block (Item Response) in the form of a survey (Fig. 1A) directly aligned to the Construct Map areas. For the third building block, we generated a scoring guide (Outcome Space). These guides are rubrics translating responses from our surveys (Items) into quantitative data or scores. Our fourth building block relates the survey scores to our development levels (Measurement or interpretational Model). We analyzed the responses to the survey items using item response theory (IRT) guided by the Construct Map (Hambleton, et al., 1991). An IRT partial credit model was applied to the survey data to give statistical evidence that the assessment was reliable and the steps in the response scale (e.g., agree strongly to agree) were ordered (Wright and Masters, 1982). We want to emphasize that this process of assessment is an iterative one. Each time we give a survey, we will go back and revise our Construct. We approach our Constructs as hypotheses that reflect progress. Each round of assessment tests these hypotheses. This scientific approach to assessment resulted in an effective final assessment instrument that we suggest can be used more generally for assessment.

References

1. Wilson, M. 2005. Constructing measures: An item response modeling approach. Erlbaum.
2. Hambleton, RK, et al., 1991. Fundamentals of item response theory. Sage Publications.
3. Wright, BD, and Masters, GN. 1982. Rating Scale Analysis. Rasch Measurement. MESA Press.

Consent Form

Please consider taking part in a research study being done by Dr. Mica Estrada at the University of California, San Francisco. Being in this study, which is funded by the Howard Hughes Medical Institute, is optional.

The survey will take less than 15 minutes to complete. You can skip questions that you do not wish to answer or stop the survey at any time. However, we hope that you will answer as many questions as you can. There are NO correct answers. We hope to assess your science, technology, engineering, and mathematics (STEM) identity and interest at present. We realize many of you are just beginning your career and may see STEM as enhancing a career in a variety of fields.

Your views are very important in helping us improve the educational experience of future students at UC Berkeley.

ASSURANCE OF CONFIDENTIALITY
All responses that relate to or describe identifiable characteristics about you will be used only for the purpose of maintaining contact with you and for statistical analyses. They will never be disclosed or used, in identifiable forms. We will keep your answers confidential and will not identify your personal responses with anyone outside the UCSF research team. This means the staff, GSIs, and faculty will never see your individual responses. Your responses will be combined with others when results are reported.

Questions? Please contact Mica Estrada, Ph.D. at mica.estrada@ucsf.edu. If you have questions or concerns about your rights as a research participant, you can call the UCSF Committee on Human Research.

By clicking agree you are agreeing to participate in this study, which includes consent to participate in this survey and future surveys, as well as permission to use your course assignments to add to our understanding of the course. If you are younger than 18 years of age please click "I do not agree".
If you want to participate in this study, click the ***Agree*** button to start the survey.

○ I Agree/I am 18+ (1)

○ I Do Not Agree/I am not 18+ (2)

Skip To: End of Survey If Thank you for taking the time to complete this survey which will help inform educational program... = I Do Not Agree/I am not 18+

End of Block: Consent

Start of Block: Current Enrollment Status

The following questions ask about your enrollment and interests.

Current Educational Status (select one)

What is your class standing at UC Berkeley in this academic term?

- 1st year in college (1)
- 2nd year in college (2)
- 3rd year in college (3)
- 4th year in college (4)
- 5th year in college (5)
- 6th year or more in college (6)
- Masters degree candidate (7)
- Doctoral degree candidate (8)
- Other (9) ____________________

Major field of study or interest (select one)

- Science (1)
- Technology (2)
- Engineering (3)
- Mathematics (4)
- Computer Science (5)
- Design (8)
- Uncertain/not sure (6)
- Other (7) ____________________

Is your current major (or intended major) biology?

- Yes (1)
- No (2)

What was your most recent GPA (i.e., grade point average in high school or college)?
____

Have you ever been part of the Biology Scholars Program (BSP) at UC Berkeley?

Yes (1)

No (2)

Faculty Mentor

MENTORING: A mentor is someone who provides guidance, assistance, and encouragement on professional and academic issues.

Is there a faculty member that you would consider a mentor?

- Yes (1)
- No (2)

Career Building Academic Experiences, Financial Support, Research Training, Intentions

In your last academic semester/term, were you involved in any research activities?

- No (1)
- Yes (2)

If Yes, please check all research activities that you participated in last academic semester/term:

- Hands-on research activities with laboratory equipment in a class (1)
- Worked in a laboratory in a college/university (2)
- Worked on research in non academic location (3)
- Designed your own research experiment (4)
- Conducted research that had an unknown outcome (5)
- Contributed to research that advanced knowledge (even if it was a very small advancement) (6)
- None of the above (7)

On a scale from 0 to 10 (0 = definitely not; 10 = definitely will)

- To what extent do you intend to pursue a science related career? (1)
- How likely is it that you will pursue a graduate degree in a science field? (2)
- To what extent do you intend to pursue a career in which you conduct research? (3)
- How likely is it that you will pursue a career that is enriched by science (i.e., informed by science but not the central work you do)? (4)

Science Identity

The following questions ask how you think about yourself and your science identity.

Please select the best answer on the scale from 1 (strongly disagree) to 7 (strongly agree).

- I have a strong sense of belonging to the community of Scientists. (1)
- I derive great personal satisfaction from working on a Science team that is doing important work. (2)
- I have come to think of myself as a Scientist. (3)
- I feel like I belong in the field of Science. (4)
- The daily work of a Scientist is appealing to me. (5)

Science Self-Efficacy

This section assesses your confidence in your abilities to function as a scientist based on your previous experiences.

Indicate the extent to which you are confident you can successfully complete the following tasks. Please select the best answer on the scale from 1 (not at all confident) to 5 (absolutely confident).

I am confident that I can...

- Use technical skills (use of tools, instruments, and/or techniques of your field of study). (1)
- Generate a research question to answer. (2)
- Determine what data/observations to collect and how to collect them. (3)
- Create explanations for the results of the study. (4)
- Use academic literature and/or reports to guide your research. (5)
- Develop theories (integrate and coordinate results from multiple studies and/or theories). (6)
- Designs experiments to test hypothesis. (7)
- Develop novel technologies. (8)

Scientific Community Values

Please read each description and think about how much each person is or is not like you.
Check the answer that best reflects how much the person in the description is like you.

- A person who thinks discussing new theories and ideas between scientists is important. (1)
- A person who believes writing up research results to be published in a leading scientific journal is a good use of time. (2)
- A person who thinks it is valuable to conduct research that builds the world's scientific knowledge. (3)
- A person who thinks that scientific research can solve many of today's world challenges. (4)
- A person who feels discovering something new in the sciences is thrilling. (5)
- A person who thinks it is important work to identify truths using the scientific method. (6)

Stereotype Threat

The next questions relate to your experiences at college. Please select the best answer on the scale from 1 (never) to 5 (almost always).

How often do you feel that because of your ethnicity...

- Some people believe that you have lower ability than other students. (1)
- People assume that you are not good enough, even if you are similar to average students. (2)
- If you do poorly on a test, people act like that is normal. (3)
- Your intelligence is not fairly evaluated. (4)

Perceived Stress Scale

The questions in this scale ask you about your feelings and thoughts during the last month. In each case, please indicate how often you felt or thought in these ways (1 = never, 5 = almost always).

In the last month, how often have you...

- Felt that you were unable to control the important things in your life? (1)
- Felt difficulties were piling up so high that you could not overcome them? (2)
- Felt confident about your ability to handle your personal problems? (3)
- Felt that things were going your way? (4)

Satisfaction with Life Scale

Below are five statements with which you may agree or disagree. Using the 1-7 scale below, indicate your agreement with each item. Please be open and honest in your responding. (1= strongly disagree, 7 = strongly agree)

- In most ways my life is close to my ideal. (1)
- The conditions of my life are excellent. (2)
- I am satisfied with my life. (3)
- So far I have gotten the important things I want in life. (4)
- If I could live my life over, I would change almost nothing. (5)

Creativity & Innovation

How much do you enjoy the following on a scale of 0 (Never Enjoy) to 10 (Always Enjoy)

- Innovation of new products (1)
- Increasing new knowledge about organisms (2)
- Being creative (3)
- Discovering new ways to do things (4)

STEM Enriched

On a scale from 1(Completely Disagree) - 7 (Completely Agree) to what extent do you agree with the following statements:

- My knowledge of science informs my work in my field (1)
- I will use the science I learned in this class in my future professional life in my field (2)
- When I see or read the news, I will use my knowledge of science to determine if it is factual. (3)
- My knowledge of science impacts how I come up with new ideas in my area of interest. (4)
- My knowledge of science impacts how I test new ideas (e.g., to see if it is useful or not). (5)
- When I hear about new scientific findings in popular media, I use my knowledge of science to determine if findings are factual. (6)

Life Choice & Finances Variables

What is your current age? Please select from the drop down menu below.

What is your gender?

- Male (1)
- Female (2)
- Non-Binary (5)
- Other (3)

How concerned are you about your ability to finance your college education?

- Not concerned (I am confident I will have enough funds) (1)
- Somewhat concerned (I will probably have enough funds) (2)
- Very concerned (I am not sure that I will have enough funds to complete my degree) (3)

- Extremely concerned (I might be leaving college due to insufficient funds) (4)

Are you Spanish/Hispanic/Latino?

- No, not Spanish/Hispanic/Latino (1)
- Yes, Mexican, Mexican American, Chicano (2)
- Yes, Puerto Rican (3)
- Yes, Cuban (4)
- Yes, other Spanish/Hispanic/Latino (5)
- Other (Please specify) (6) ____

Please check one or more races to indicate what you consider yourself to be:

- White (1)
- Black/African American (2)
- American Indian or Alaska Native (3)
- Asian Indian (4)
- Japanese (5)
- Native Hawaiian (6)
- Chinese (7)
- Korean (8)
- Guamanian or Chumorro (9)
- Filipino (10)
- Vietnamese (11)
- Samoan (12)
- Other Asian (13) ____
- Other Pacific Islander (14) ____
- Some other race (15) ____

We appreciate your feedback and ask for your Cal student ID to verify your enrollment in the course. Before this data are reported, your student ID will be removed and kept in a unique data file.

Language

Do you speak any other language besides english?

- No (1)
- Yes (2)

If yes, what other language do you speak?

____

At what level do you speak the above language?

- Beginner (1)
- Intermediate (2)
- Advanced (3)
- Proficient (4)

Do you read any other language besides english?

- No (1)
- Yes (2)

If yes, what other language do you read?

____

At what level do you read the above language?

- Beginner (1)
- Intermediate (2)
- Advanced (3)
- Proficient (4)

Covid

We understand the recent COVID-19 pandemic has brought many changes to the the world and the way people work and interact. The following questions ask you to reflect on how the pandemic has impacted you and your academic work.

Changes to my home life due to the COVID-19 pandemic have greatly impacted my ability to work.

- Strongly Disagree (1)
- Disagree (2)
- Neutral (3)
- Agree (4)
- Strongly Agree (5)

Since the COVID-19 pandemic began, what has changed for you? [check all that apply]

- Increased workload or work responsibilities. (1)
- Decreased workload or work responsibilities (4)
- Disruptions to work (e.g. childcare needs in home, helping child with schoolwork, caring for family members) (5)
- Lack of equipment or resources (e.g., computers or wifi) to work efficiently and effectively (6)
- Difficulties concentrating (7)
- Disruption of communication and connection with colleagues ("Zoomed out") (8)
- Increased free time (9)
- Increased productivity (10)
- Increased financial stress (11)
- Increased overall stress (12)
- Increased racial discrimination (13)
- Strengthened relationships with others (14)
- More flexibility in schedule (15)
- Separated from family and friends (Quarantining alone) (16)
- Other (please specify) (17) ____

Is there anything else you want us to know about how the COVID-19 pandemic has impacted your professional/academic life in the past 6 months?

____

____

____

____

21st Century Skills

Respond to the following statements by indicating (1) strongly disagree, (2) disagree, (3) undecided, (4) agree, or (5) strongly agree.

- I read scientific publications to extract specific facts. (1)
- I use interdisciplinary concepts and methodologies to advance my own discipline. (2)
- I take a leadership role in interdisciplinary teams. (3)
- I read scientific publications when they are required. (4)
- I take an active role in interdisciplinary collaborations. (5)
- I can understand the logic of scientific publications when discussed by experts. (6)
- I seek interdisciplinary collaborations to attain technical or factual knowledge outside discipline. (7)
- I can translate fundamental principles from scientific publications to create novel designs. (8)
- I am aware of concepts and methodologies across disciplines. (9)
- I prefer collaboration among members of my discipline. (10)
- I seek concepts and methodologies from other fields. (11)
- I recognize the benefits from interacting with other disciplines. (12)

Respond to the following statements by indicating (1) strongly disagree, (2) disagree, (3) undecided (4) a gree, or (5) strongly agree.

- I can understand how experts have translated principles from scientific publications into novel designs. (1)
- I contribute to and benefit from interdisciplinary collaborations. (2)
- I seek information from other disciplines when they are required. (3)
- I can apply concepts and methodologies across disciplines. (4)
- I seek information from my own discipline to solve problems. (5)
- I seek opportunities outside the discipline. (6)

- I can extract fundamental principles from scientific publications. (7)
- I attempt to apply concepts and methodologies across disciplines. (8)
- I primarily use technical knowledge in my own discipline. (9)
- I can suggest fundamental principles from scientific publications that could translate into novel designs. (10)
- I participate in interdisciplinary teams when required. (11)
- I contribute to advancements in other fields with knowledge from my discipline. (12)

Respond to the following statements by indicating (1) strongly disagree, (2) disagree, (3) undecided, (4) a gree, or (5) strongly agree.

- I can critically evaluate scientific publications. (1)
- I participate in diverse teams because I recognize the benefits of interdisciplinary knowledge. (2)

Supplement S17 Results from 21st Century Skill Assessment

Our 26 item Likert-type pre/post course survey (see Supplement 16) is a self-reported measure of students' 21st century skills with 514 pre- and 432 post-survey responses. Our preliminary analysis of pre/post changes in raw Likert scores showed increases in "agreeability" for all items, including those mapped to the highest levels of the construct map (i.e., items that we considered "hardest" to agree with). Thus, the mean score for each item on the survey increased from pre to post, resulting in a positive delta (Δ ± one standard deviation (SD)) value for each item. Students showed growth in all skills and subdimensions of our 21st century skills construct every year after completing the course.

The reliability of our 21st Century Skills assessment is supported by a high Weighted Likelihood Estimates (WLE) person separation reliability (0.93), a high expected a posteriori/plausible value (EAP/PV) reliability (0.90), and a high coefficient (Cronbach's) alpha (0.92). We plan to evaluate the validity of this assessment in a future publication.

Scientific Discovery & Translation Process

(1) strongly disagree, (2) disagree, (3) neutral, (4) agree, or (5) strongly agree

**Level**	***Skill***	***Mean Before***	***Post – Pre Δ***	***± Δ SD***
Leader	Can extract fundamental principles from scientific publications.	3.67	0.46	1.13
	Can translate fundamental principles from scientific publications to create novel designs.	3.11	0.88	1.26
Active	Can critically evaluate scientific publications.	3.36	0.58	1.30
	Can suggest fundamental principles from scientific publications that could translate into novel designs.	3.21	0.79	1.23
Participant	Can understand the logic of scientific publications when discussed by experts.	3.59	0.30	1.23
	Can understand how experts have translated principles from scientific publications into novel designs.	3.65	0.54	1.13
Technical	Reads scientific publications to extract specific facts.	3.30	0.61	1.39
Required	Reads scientific publications because required.	4.17	0.15	1.01

Pre: $N = 514$, Post: $N = 432$

Interdisciplinary Thinking

(1) strongly disagree, (2) disagree, (3) neutral, (4) agree, or (5) strongly agree

Level	*Skill*	*Mean Before*	*Post – Pre Δ*	*± Δ SD*
Leader	Apply concepts and methodologies across disciplines.	3.82	0.31	1.05
	Uses interdisciplinary concepts and methodologies to advance own discipline.	3.88	0.29	1.14
	Contributes to advancement in other fields with knowledge from my discipline.	3.54	0.53	1.22
Active	Attempt to apply concepts and methodologies across disciplines.	3.86	0.29	1.05
	Seeks concepts and methodologies from other fields.	3.90	0.25	1.09
Participant	Recognizes the benefits from interacting with other disciplines.	4.57	0.03	0.93
	Aware of concepts and methodologies across disciplines.	3.46	0.56	1.19
Technical	Primarily uses technical knowledge in own discipline.	3.53	0.27	1.39
Required	Seeks information from own discipline to solve problems.	4.12	0.18	1.00
	Seeks information from disciplines other than their own when they are required.	4.23	0.15	0.94

Pre: $N = 514$, Post: $N = 432$

Interdisciplinary Collaboration

(1) strongly disagree, (2) disagree, (3) neutral, (4) agree, or (5) strongly agree

Level	*Skill*	*Mean Before*	*Post – Pre Δ*	*± Δ SD*
Leader	Leadership role in interdisciplinary teams.	3.69	0.44	1.22
	Contributes to and benefits from interdisciplinary collaborations.	3.99	0.31	1.08
Active	Active role in interdisciplinary collaborations.	3.96	0.32	1.11
	Seeks opportunities outside the discipline.	3.87	0.29	1.16
Participant	Participates in diverse teams because recognizes the benefits of interdisciplinary knowledge.	4.30	0.17	0.99
Technical	Seek interdisciplinary collaborations to attain technical or factual knowledge outside discipline.	3.77	0.38	1.19
Required	Prefer collaboration among members of my discipline.	3.41	0.07	1.42
	Participates in interdisciplinary teams when required.	4.26	0.16	0.97

Pre: $N = 514$, Post: $N = 432$
